# CircRNA Microarray Profiling Reveals hsa_circ_0058493 as a Novel Biomarker for Imatinib-Resistant CML

**DOI:** 10.3389/fphar.2021.728916

**Published:** 2021-09-13

**Authors:** An-Ni Zhong, Yi Yin, Bing-Jie Tang, Lei Chen, Hong-Wei Shen, Zhi-Ping Tan, Wen-Qun Li, Qun He, Bao Sun, Yan Zhu, Jie Xiao, Zhi-Ping Jiang, Ping Xu

**Affiliations:** ^1^ Department of Pharmacy, The Second Xiangya Hospital, Central South University, Changsha, China; ^2^ Institute of Clinical Pharmacy, Central South University, Changsha, China; ^3^ Department of Pharmacy, First Affiliated Hospital of Guangxi Medical University, Nanning, China; ^4^ Medical Experiment Research Centre, The Second Xiangya Hospital, Central South University, Changsha, China; ^5^ Department of Cardiovascular Surgery, The Second Xiangya Hospital, Central South University, Changsha, China; ^6^ Department of Hematology, Xiangya Hospital, Central South University, Changsha, China; ^7^ Department of Pharmacy, Affiliated Hospital of Zunyi Medical University, Zunyi, China

**Keywords:** imatinib, chronic myelogenous leukemia, hsa_circ_0058493, exosome, drug resistance

## Abstract

**Background:** CircRNA has appeared as a critical molecular in the development of various cancers. However, the cellular function of circRNAs and exosomal circRNAs has not been well explored in Chronic myeloid leukemia (CML).

**Methods:** Differentially expressed circRNAs were identified by a human circRNA microarray analysis. The expression of hsa_circ_0058493 in peripheral blood mononuclear cells (PBMCs) and exosomes was verified using quantitative real-time PCR. Short hairpin RNAs against hsa_circ_0058493 were constructed to silence the expression of circ_0058493. CCK8, flow cytometry and EdU assay were performed to investigate the biological functions of circ_0058493.

**Results:** Hsa_circ_0058493 was significantly overexpressed in the PBMCs of CML patients and high level of circ_0058493 was associated with the poor clinical efficacy of imatinib. Silencing the expression of circ_0058493 significantly inhibited the development of imatinib-resistant CML cells. miR-548b-3p was overexpressed in circ_0058493-downregulated CML cells. Bioinformatic analysis revealed that circ_0058493 might exert its regulatory function acting as a “sponge” of miR-548b-3p. Moreover, hsa_circ_0058493 was significantly enriched in the exosomes derived from imatinib-resistant CML cells.

**Conclusion:** Hsa_circ_0058493 in PBMCs could be a promising prognostic biomarker and might provide a therapeutic target for CML treatment.

## Introduction

Chronic myeloid leukemia (CML) is a myeloproliferative malignancy arising from BCR-ABL1 oncoprotein (Apperley, 2015). Imatinib mesylate, one of the tyrosine kinase inhibitors (TKIs), was approved for the front-line treatment of CML and largely improved the prognosis of CML patients (Jabbour, 2016). However, the clinical response of imatinib in CML patients remained unsatisfied due to the occurrence of treatment failure or intolerance (Geelen et al., 2017). The secondary mutation in BCR-ABL1 has illuminated the resistance of imatinib and can be treated with second or third-generation TKIs (Apperley, 2015). However, there is no mutation in BCR-ABL1 in 50% or more cases of treatment failure, and the underlying mechanisms of BCR-ABL1-independent resistance to imatinib remain poorly understood (Ma et al., 2014). Therefore, it is essential to elucidate the molecule mechanism of imatinib resistance and explore a novel prescient biomarker or treatment target to improve the prognosis of CML patients.

Circular RNAs (circRNAs) are a substantial category of endogenous non-coding RNA molecules characterized by covalently closed loop structures. CircRNA has appeared as a critical molecular in the emerging, progression, and resistance of cancer due to its wide existence, high stability, and diversity of regulatory functions (Qu et al., 2015). The major function of circRNAs acts as “sponge” combining with miRNAs, thus regulating the translation of target mRNAs, and further participating in tumor progression and drug resistance (Qu et al., 2015; Huang et al., 2019; Jian et al., 2020). More importantly, it has been demonstrated that intercellular transmission via exosomes is an important way in which circRNAs mediate tumor progression and drug resistance (Huang et al., 2020; Wang et al., 2020). Exosomes are the subtype of extracellular vesicles (EVs) and are typically 30–150 nm in diameter (Doyle and Wang, 2019). Exosomes can be secreted by all cell types and have been found in nearly all body fluids including plasma and serum (Doyle and Wang, 2019). Studies have shown that serum exosomal circRNAs are relevant to the clinical efficacy of patients with cancer, serving as promising biomarkers for liquid biopsy (Xu X. et al., 2020; Xie et al., 2020). However, the cellular functions of circRNAs and exosomal circRNAs in CML have not been well explored.

In this study, we showed that high level of hsa_circ_0058493 in PBMCs was correlated with poor clinical efficacy of CML. Silencing circ_0058493 expression can observably restore the drug sensitivity of imatinib-resistant cells. Besides, hsa_circ_0058493 was significantly enriched in the exosomes derived from imatinib-resistant cells. This evidence suggested that hsa_circ_0058493 in PBMCs might serve as a novel prognostic biomarker in CML treatment.

## Materials and Methods

### Patient Samples

The peripheral blood samples used in this study (*n* = 90) from CML patients were collected at the hematology department of Xiangya Hospital and Second Xiangya Hospital of Central South University from July 2016 to December 2018. All patients were diagnosed as Philadelphia chromosome positive CML chronic phase (Ph^+^ CML-CP) without BCR-ABL kinase mutation or other tumor diseases. These patients received an oral dose of 400 mg imatinib (IM) once daily. The clinical efficacy of imatinib in CML patients was assessed based on “The guidelines for diagnosis and treatment of chronic myelogenous leukemia in China (2016 edition) (Chinese Society of Hematology and Chinese Medical Association, 2016)”. Plasma of six patients with optimal clinical response (IM optimal responders) and six patients with no response (IM nonresponders, consisting of warning and treatment failure) were collected for microarray analysis. The PBMCs of 90 plasma samples were extracted for further verification. The clinicopathological features including sex, age, BMI, and clinical efficacy, were shown in Table 1 and Supplementary Table S1. We collected 5 ml peripheral blood sample for research when the CML patients underwent routine blood test or plasma drug concentration detection. The blood samples were temporarily stored at 4°C. PBMCs were extracted by density gradient centrifugation within 1 week of peripheral blood sample collection. The plasma and PBMCs samples were stored at −80°C for long-term storage.

**TABLE 1:** Relationship between hsa_circ_0058493 expression and clinicopathologic factors of patients with CML.

Parameter	circ_0058493 expression		*p*-value (*p < 0.05)
	Low	High	
	*n* = 42	*n* = 48	
Sex			0.8312
Femal	18	19	
Male	24	29	
Age, Year			0.3678
< 40	20	20	
40∼ < 60	19	27	
≥60	3	1	
BMI			0.509
< 18.5 kg/m^2^	3	1	
18.5∼ < 25 kg/m^2^	24	29	
≥25 kg/m^2^	15	18	
clinical efficacy			0.0014^*^
optimal response (*n* = 50)	31	19	
warning and no response (*n* = 40)	11	29	

The protocol was in accordance with the Declaration of Helsinki and written informed consents were obtained from all the patients participating in the trial. This study was approved by the Medical Ethics Committee of the Second Xiangya Hospital of Central South University (Approval No.: XY2-PK-TKI-2016A01).

### CircRNA Microarray Analysis

The sample preparation and microarray hybridization were performed based on Arraystar’s standard protocols. Briefly, total RNAs were digested with Rnase R (Epicentre, Inc.) to remove linear RNAs and enrich circular RNAs. Then, the enriched circRNAs were amplified and transcribed into fluorescent cRNA labeled with an Arraystar Super RNA Labeling Kit. The labeled cRNAs were hybridized onto the Arraystar Human circRNA Array V2 (8x15K, Arraystar) and scanned by the Agilent Scanner G2505C. Microarray scanning and data analyses were conducted by Kang Chen Biotech (China).

When comparing two groups of profile differences, the “fold change” (i.e. the ratio of the group averages) between the groups for each circRNA is computed. The statistical significance of the difference may be conveniently estimated by *t*-test. circRNAs having fold changes ≥2 and *p*-values ≤0.05 are selected as significantly differentially expressed.

### Cell Culture and Lentivirus Transfection

Imatinib-sensitive K562 cells were kindly given by Institute of Clinical Pharmacology (Central South University), imatinib-resistant K562/G01 cells were kindly given by Tianjin Institute of Hematology. K562 and K562/G01 cells were cultured in RPMI 1640 cell medium (Gibco, United States) supplemented with 10% fetal bovine serum (FBS, Gibco, South America) and 1% penicillin-streptomycin (Gibco, United States) mixture maintained at 37°C in a cell incubator with a humidified atmosphere containing 5% CO2. K562/G01 was cultured with 4 μM imatinib to keep its resistance, and removed imatinib treatment 1 week before experiment.

For hsa_circ_0058493 downregulation, K562/G01 cells were infected with lentivirus vector (GV493) carrying short hairpin RNA (shRNA) against hsa_circ_0058493 (sh-circ_0058493) and its negative control (sh-NC) (Genechem, Shanghai, China) following the manufacturer’s instructions. Puromycin (biosharp, China) were added into cells after transfection to screen successfully transfected cells. The sequence of sh-circ_0058493: sh-circ_0058493-1, CTC​ACC​AGG​GTT​CAG​CCG​T; sh-circ_0058493-2, TCA​CCA​GGG​TTC​AGC​CGT​C; sh-circ_0058493-3, TTT​ATT​CTC​ACC​AGG​GTT​C, and the one with the best knockdown efficiency would be chosen to conduct the subsequent experiments.

### RNA Preparation and Quantitative Real-Time PCR

Total RNA was extracted from cultured cells, exosomes derived from cell lines and PBMC of CML patients respectively using TRIzol reagent (Takara, Japan). Total RNA was reverse transcribed using Evo M-MLV RT Kit with gDNA Clean for q-PCR Ⅱ (Accurate Biology, China). Quantitative real-time polymerase chain reaction (RT-qPCR) was performed using TB Green™ Premix DimerEraser™ (Perfect Real Time) kit (Takara, Japan) on the Applied Biosystems™ 7500 Fast Dx Real-Time PCR system (Thermo, MA, United States). Relative expression levels of genes were normalized to GAPDH expression and calculated using the 2 −ΔΔC t method. All steps were conducted according to the manufacturer’s instructions. The sequence of circ_0058493: forward primer, TGG​GCT​TTC​CTG​TAC​CGA​AC; reverse primer, CTC​CGT​TGC​ATG​GCC​AGA​TA. The sequence of GAPDH: forward primer, CTG​AGA​ACG​GGA​AGC​TTG​TCA; reverse primer, CCA​GTG​GAC​TCC​ACG​ACG​TA.

### Cell Counting Kit-8 Assay

Cell proliferation was determined by Cell Counting Kit-8(CCK-8) (Meilunbio, China), 2 × 10^4^ cells were seeded in the 96-well plate and were treated with different concentration of imatinib (10, 5, 2.5, 1, 0.5, 0.25, 0.1 μM, 0). The treated cells were incubated at 37°C, 5%CO2 for 48h, then 10 μl CCK-8 solution was added to each well. After 1 h of incubation at 37°C, the absorbance at 450 nm was measured using a Thermo Scientific Microplate Reader (Thermo Fisher, MA, United States).

### 5-Ethynyl-2′-Deoxyuridine Incorporation Assay

EdU assays were performed with a BeyoClickTM EdU-555 Cell Proliferation Kit (Beyotime, China). The transfected cells were seeded into 6-well plates (50 × 10^4^ cells/well) and treated with 4 μM imatinib for 48 h. After incubation with 220 μl 10 × EdU for 2 h, the cells were fixed in 4% paraformaldehyde and penetrated with 0.3% Triton X-100 solution (BIOFROXX, Germany). Hoechst 33342 was used to stain the nucleic acids within the cells. Images were obtained with a fluorescence microscope (Carl Zeiss Microscopy GmbH, Germany), and the proportion of EdU-positive cells was counted.

### Cell Apoptosis Analysis

With the detection of apoptosis by flow cytometry, an AnnexinV-PE/7-AAD Cell Apoptosis Kit (Meilunbio, China) was used to analyze cellular apoptosis. Cells were seeded in 12-well plates (10×10^4^ cells/well) and treated with 10 μM imatinib for 48 h, washed with phosphate-buffered saline (PBS) two times, suspended in 100 μl 1×Binding Buffer, and then incubated with 5 μl Annexin V-PE and 7 μl 7-AAD for 15 min at room temperature in the dark. The stained cells were diluted with 200 μl 1 × Binding Buffer and detected using the BD FACSCanto II flow cytometer (BD Biosciences, NJ, United States) within 1 h.

### Bioinformatic Analysis

The circRNA/microRNA interaction was predicted with Circbank (http://www.circbank.cn/) and Circinteractome (https://circinteractome.nia.nih.gov/) online database. The miRNAs predicted by both databases were chosen for the further study. The target mRNAs of microRNA were predicted with TargetScan and MiRDB online database.

### Cell Exosome Isolation and Identification

Exosomes were extracted from cell culture medium using ultracentrifugation. K562 and K562/G01 cells were cultured for 48h, then supernatants were collected respectively. FBS was centrifuged at 130000 g for 16 h at 4°C to remove exosomes, then filtered through 220 nm filter to remove bacterium. The cell supernatants were centrifuged at 300 g for 15 min, 2000 g for 20 min, 10000 g for 60 min at 4°C to remove cell debris. Then the supernatants were filtered through 220 nm filter to remove larger EVs. Then the exosomes were collected after ultracentrifugation at 110,000 g, 70 min at 4°C (SW41 rotor, Beckman Coulter, CA, United States). The pellets were resuspended in PBS and collected by ultracentrifugation at 110,000 g, 70 min at 4°C. The exosomes were ready for protein or RNA extraction. The exosome pellet can be stored at −80°C (preferably within a week) until ready for further analysis (Andaluz Aguilar et al., 2020).

Exosomes were then identified by Transmission Electron Microscope (TEM) for particle size and form. Exosome protein markers were identified by western blot assay. The total amount of exosomes and particle diameter were detected by nanoparticle tracking analysis (NTA) (Malvern, Britain). Western blot analysis was performed to identify exosomal markers CD63 and TSG101.

### Statistical Analysis

The statistical results were exhibited as mean ± standard deviation (SD) of three independent experiments. Student’s *t*-test and Chi-square test were used to determine the statistical significance for comparisons of two or more groups. All statistical analyses were performed using IBM SPSS Statistics 22 (SPSS Inc., United States) and GraphPad Prism 8 software (GraphPad Software, United States). The graphs were generated by GraphPad Prism 8 software. The *p*-values < 0.05 were considered statistically significant.

## Results

### Differentially Expressed circRNAs Are Associated With the Clinical Efficacy of CML Patients

**FIGURE 1:**
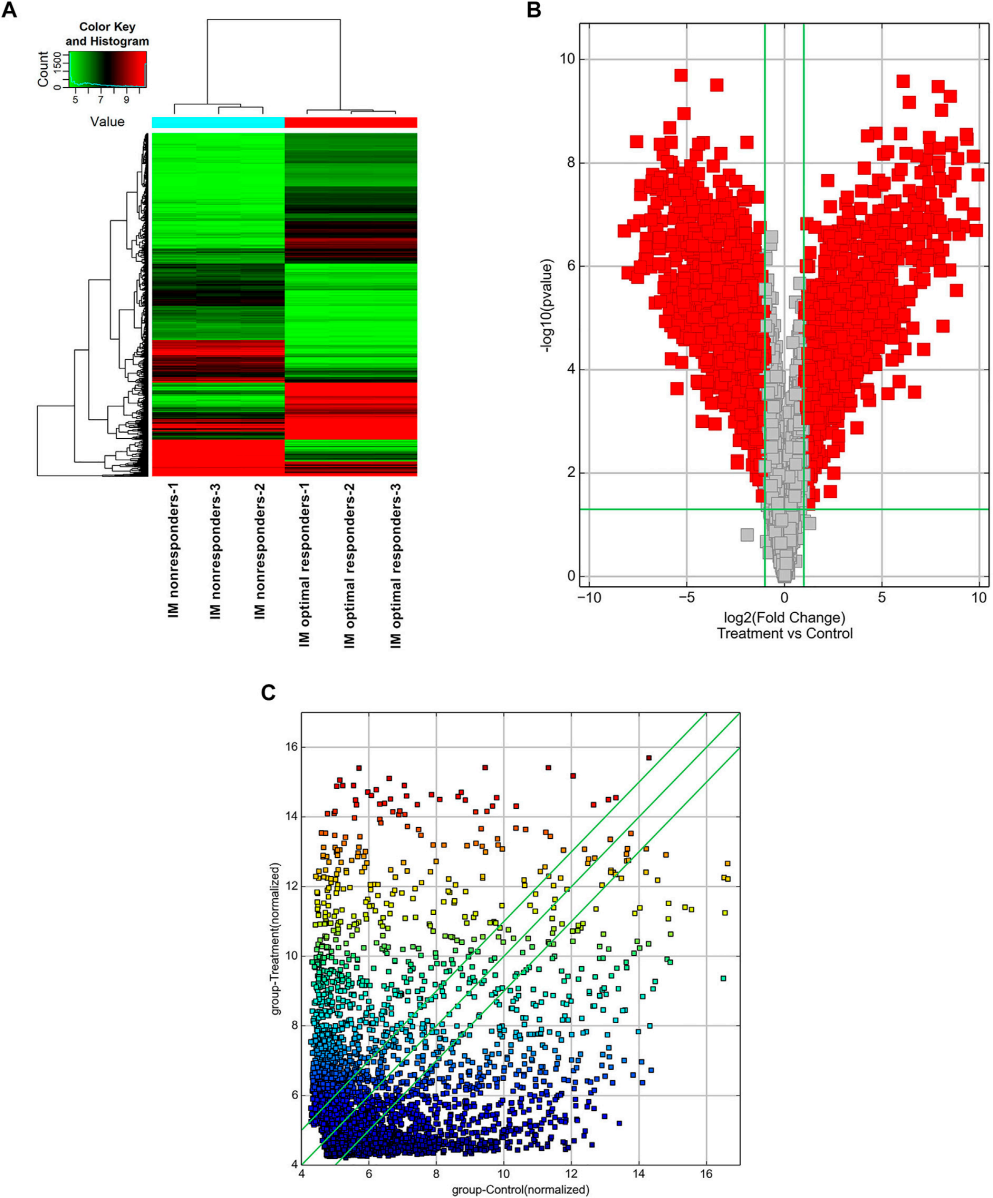
Profiling of circular RNAs in the plasma from CML patients with different clinical responses to imatinib. **(A)** Heat map shows the upregulated and downregulated circRNAs in IM optimal responders vs IM nonresponders. Each column represents the expression profile of a plasma sample group, and each line corresponds to a circRNA. High expression level is indicated by “red” and lower levels by “green”. **(B)** Volcano plot shows the up-regulated and down-regulated circRNAs in IM optimal responders (control) vs IM nonresponders (treatment). The abscissa is the log2 (Fold Change) of the circRNA level difference between samples, the ordinate is -log10 (*p*-value). circRNAs having fold changes ≥2 and *p*-values ≤ 0.05 are indicated by “red”. **(C)** Scatter plot shows the up-regulated and down-regulated circRNAs in IM optimal responders (abscissa, control) vs IM nonresponders (ordinate, treatment). circRNAs having fold changes ≥2 and *p*-values ≤ 0.05 are distinguished by line 1 and line 3.

High-throughput human circRNA microarray was conducted using plasma samples from 12 CML patients to assess circRNA expression profiles among CML patients with different clinical efficacy. The plasma samples from six IM optimal responders were mixed and divided into three groups (labeled IM optimal responders -1/2/3), the plasma samples from IM nonresponders were treated the same way (labeled IM nonresponders -1/2/3). Compared with the IM optimal responders, 1045 significantly upregulated and 1292 downregulated circRNAs were detected in the plasma samples of IM nonresponders (Figures 1A,B,C). Then we further analyzed the expression of top 15 statistically upregulated (Table 2) and downregulated (Table 3) circRNAs in imatinib-resistant cell line K562/G01 and imatinib sensitive cell line K562 by RT-qPCR and found that eight circRNAs were significantly upregulated in K562/G01 compared with K562, which were in accordance with the microarray results. Three significantly up-regulated circRNAs with fold change >5 were chosen for further verification, they were hsa_circ_0058493, hsa_circ_0001402, and hsa_circ_0008274.

**TABLE 2:** Differential expression of top 15 up-regulated circRNAs in imatinib-resistant cell lines K562/G01 vs imatinib sensitive cell lines K562

circRNA	Regulation	FC(Fold change)	*p*-value
**hsa_circ_0058493**	**Up**	**8.52**	<0.001***
**hsa_circ_0008274**	**Up**	**6.89**	<0.001***
**hsa_circ_0001402**	**Up**	**8.68**	0.002**
hsa_circ_0083054	Up	3.97	0.027*
hsa_circ_0004885	Up	3.21	0.016*
hsa_circ_0002191	Up	2.93	0.007**
hsa_circ_0030062	up	2.34	0.005**
hsa_circ_0049613	up	1.47	0.009**
hsa_circ_0006614	up	1.47	0.151
hsa_circ_0042819	up	1.48	0.284
hsa_circ_0000595	up	1.26	0.815
hsa_circ_0001788	up	1.17	0.330
hsa_circ_0038737	up	1.08	0.771
hsa_circ_0009117	down	1.48	0.044*
hsa_circ_0077398	Undetected	/	/

*p < 0.05, **p < 0.01, ***p < 0.001 (Student’s t-test). Bold: fold change > 5.

**TABLE 3:** Differential expression of top 15 down-regulated circRNAs in imatinib-resistant cell lines K562/G01 vs imatinib sensitive cell lines K562

circRNA	Regulation	FC(Fold change)	*p*-value
hsa_circ_0020934	Down	1.11	0.788
hsa_circ_0026686	Down	1.03	0.897
hsa_circ_0024517	Down	1.02	0.979
hsa_circ_0044097	Down	1.00	0.887
hsa_circ_0010921	Up	1.23	0.971
hsa_circ_0000367	Up	1.29	0.254
hsa_circ_404,457	up	1.82	0.112
hsa_circ_0068367	up	1.85	0.241
hsa_circ_0020929	up	2.12	0.013*
hsa_circ_0056754	up	2.38	0.029*
hsa_circ_0045881	up	3.18	0.020*
hsa_circ_0074660	Undetected	/	/
hsa_circ_0008784	Undetected	/	/
hsa_circ_0010553	Undetected	/	/
hsa_circ_0062142	Undetected	/	/

*p < 0.05 (Student’s t-test).

### Characterization of hsa_circ_0058493 and Its Expression in CML

**FIGURE 2:**
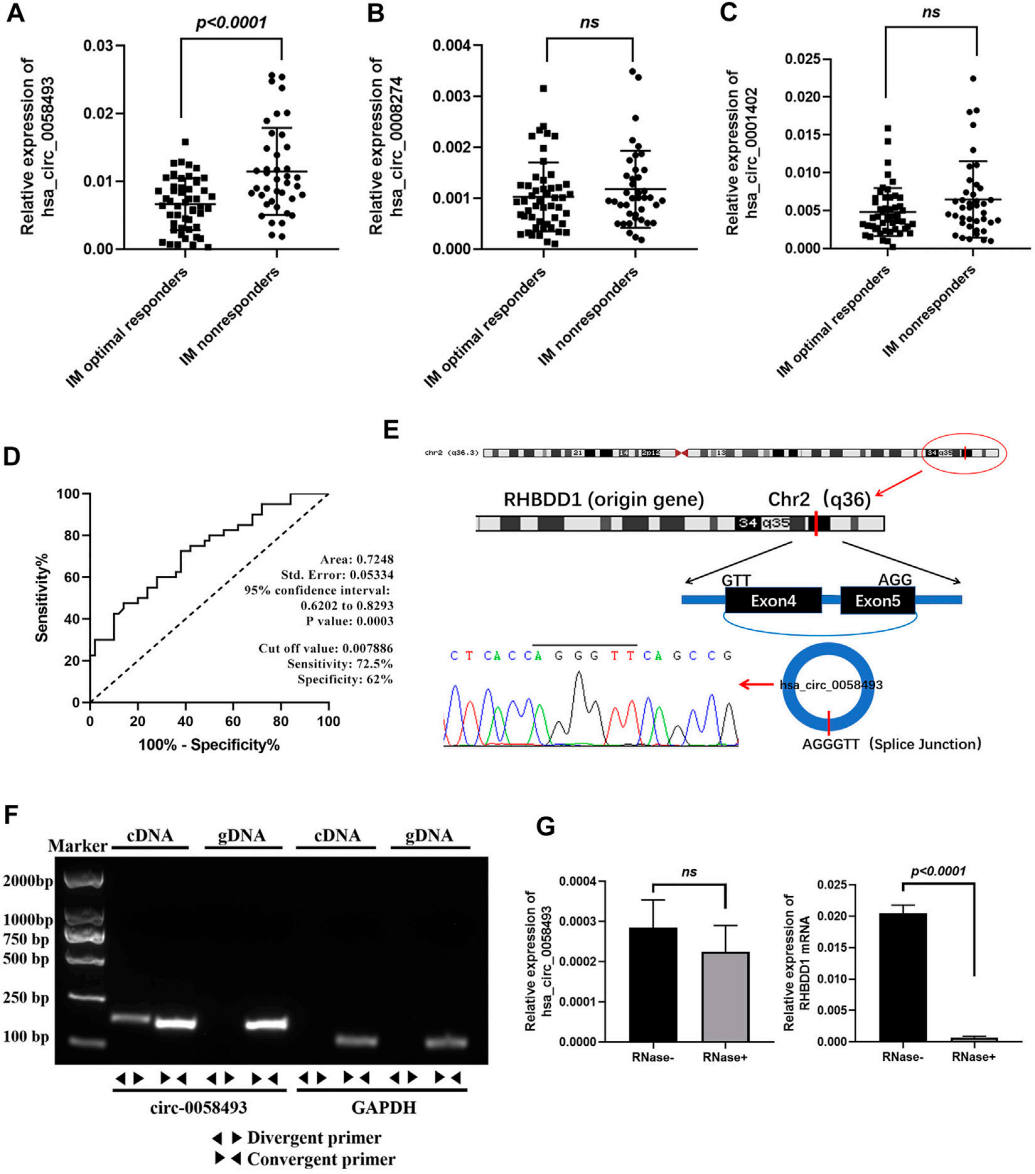
Identification of hsa_circ_0058493 as a biomarker for CML. **(A)** hsa_circ_0058493 expression was significantly upregulated in PBMCs of IM nonresponders compared to IM optimal responders (*p* < 0.0001). **(B)** hsa_circ_0008274 expression showed no statistical difference between the two groups. **(C)** hsa_circ_0001402 expression showed no statistical difference between the two groups. **(D)** The value of hsa_circ_0058493 as a prognostic biomarker in CML was evaluated by ROC curve analysis. The AUC of hsa_circ_0058493 was 0.7248, the cut-off value was 0.007886 (sensitivity: 72.5%; specificity: 62%). **(E)** The origin of hsa_circ_0058493 was obtained from circBase online database. The splice junction sequence of hsa_circ_0058493 was verified by Sanger sequencing. **(F)** Divergent primers amplified hsa_circ_0058493 in cDNA but not in genomic DNA (gDNA). GAPDH served as a negative control. **(G)** RNA from K562/G01 cells was treated with or without RNase R for RT-qPCR. The relative levels of circ_0058493 and RHBDD1 mRNA were normalized to the values of GAPDH measured in the RNase R-group. Quantitative data from three independent experiments are shown as the mean ± SD (error bars). ns: not statistically significant (Student’s *t*-test).

We further expanded the sample size to 90 (including 50 IM optimal responders and 40 IM nonresponders), to measure the expression of these three differentially expressed circRNAs in patients’ PBMC. The RT-qPCR results showed that hsa_circ_0058493 expression was significantly upregulated and was 1.75-fold higher in the PBMCs of IM nonresponders compared with IM optimal responders (*p* < 0.0001) (Figures 2A,B,C).

The value of hsa_circ_0058493 as a prognostic biomarker in CML was evaluated by ROC curve analysis. The AUC of hsa_circ_0058493 was 0.7248, the cut-off value was 0.007886 (sensitivity: 72.5%; specificity: 62%), the results indicated that hsa_circ_0058493 has a medium ability to predict the efficacy of patients with CML (Figure 2D). Besides, we divided CML patients into high-circ_0058493 and low-circ_0058493 groups according to the cut-off value mentioned above, high level of circ_0058493 was correlated with poor clinical efficacy of CML patients (Table 1).

The data from circBase showed that hsa_circ_0058493 was derived from RHBDD1 gene exon 4-5. The splice junction of circ_0058493 was verified by Sanger sequencing (Figure 2E). To excluded possibilities of genomic rearrangements, we designed convergent primers to amplify RHBDD1 mRNA and divergent primers to amplify circ_0058493 via agarose gel electrophoresis. Using cDNA (complementary DNA) and genomic DNA (gDNA) from K562/G01 cell lines as templates, the circ_0058493 amplification product was only observed in cDNA by divergent primers but not in gDNA, RHBDD1 amplification product was only observed in cDNA and gDNA by convergent primers but not by divergent primers (Figure 2F). Resistance to digestion by Ribonuclease R (RNase R) compared with RHBDD1 mRNA verified the stability of circ_0058493 and excluded possibilities of *trans*-splicing (Figure 2G).

These consequences revealed that high level of hsa_circ_0058493 in PBMCs was associated with the poor prognosis of CML, making it a potential prognostic biomarker for CML treatment.

### Hsa_circ_0058493 Promotes Imatinib Resistance in CML Cell Lines

**FIGURE 3:**
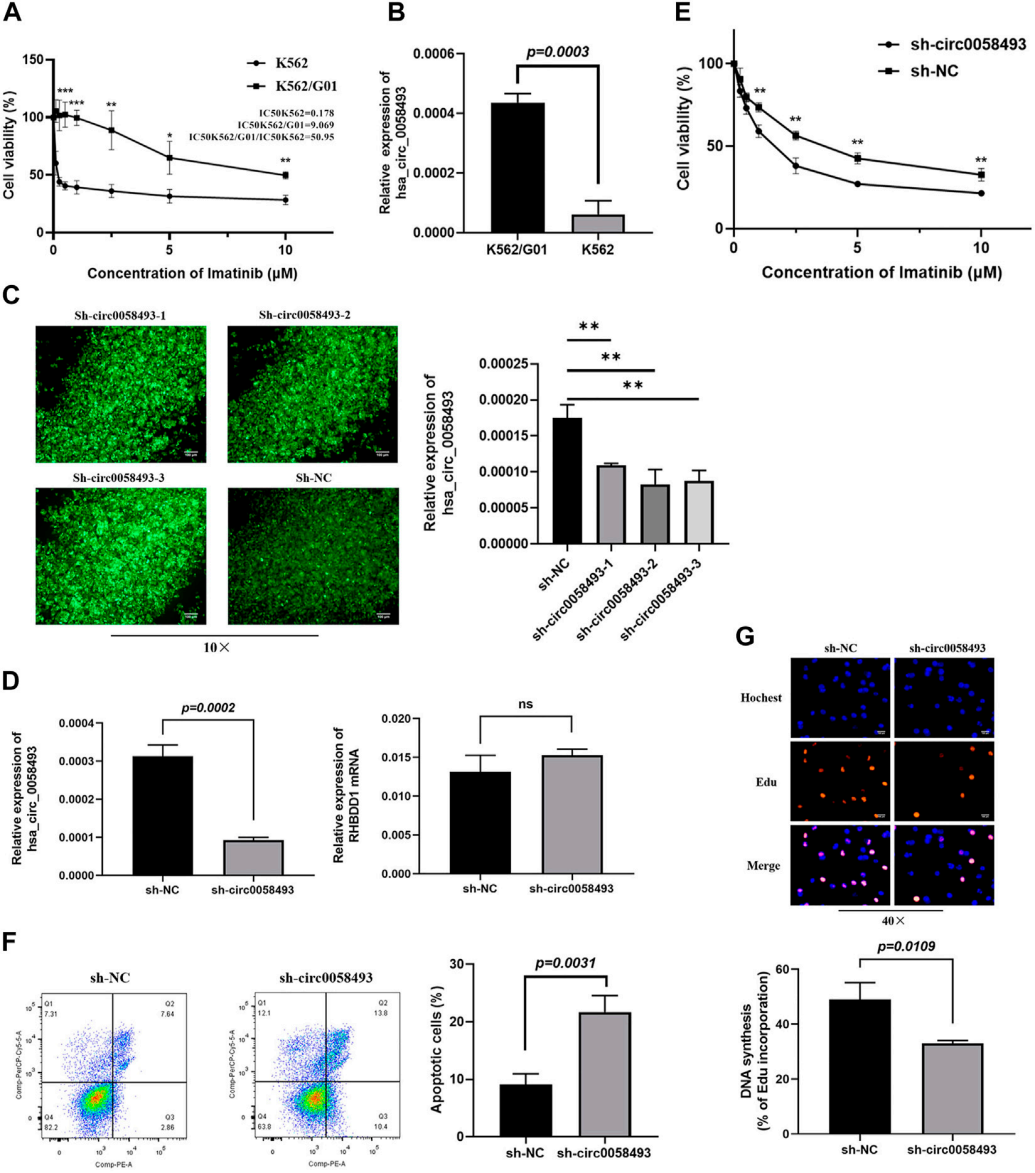
Hsa_circ_0058493 promotes imatinib resistance *in vitro*. **(A)** The sensitivity of K562 and K562/G01 to imatinib. **(B)** circ_0058493 was significantly upregulated in K562/G01 compared with K562 (*p* = 0.0003). **(C)** Fluorescence images of K562/G01 cells after transfected with sh-circ0058493-1, sh-circ0058493-2, sh-circ0058493-3, and sh-NC; RT-qPCR showed that all the three sh-circ0058493 had the significant interference efficiency against circ0058493. **(D)** sh-circ_0058493 was transfected into K562/G01 by lentivirus and obviously downregulated the level of circ_0058493 (*p* = 0.0002), while the expression level of RHBDD1 wasn’t affected by sh-circ_0058493. **(E)** CCK-8 experiment showed the cell viability of K562/G01 was obviously inhibited at different IM concentrations after circ_0058493 downregulation. **(F)** Flow cytometric assay showed the apoptosis rate of K562/G01 cells was obviously increased after circ_0058493 downregulation (*p* = 0.0031). **(G)** EdU experiment showed the proliferation ability of K562/G01 cells was obviously inhibited after circ_0058493 downregulation (*p* = 0.0109). Quantitative data from three independent experiments are shown as the mean ± SD (error bars). *p < 0.05, **p < 0.01, ***p < 0.001, ns: not statistically significant (Student’s *t*-test).

Imatinib-resistant K562/G01 cells showed obvious imatinib resistance, as indicated by elevated IC50 value and cell viability compared to imatinib-sensitive K562 cells (Figure 3A). RT-qPCR analysis revealed that the expression of circ_0058493 was 8.52-fold higher in K562/G01 on average than in K562 (*p* < 0.001) (Figure 3B). Therefore, we used K562 and K562/G01 cells in the following analyses.

To investigate the role of circ_0058493 in the drug resistance of CML, we constructed lentivirus carrying short hairpin RNA (shRNA) against hsa_circ_0058493 (sh-circ_0058493) and its negative control (sh-NC) and transfected them into K562/G01 respectively. The RT-qPCR assay showed that all the three sh-circ0058493 had the significant knockdown efficiency against circ_0058493, and we chose sh-circ0058493-2 to conduct the subsequent experiments (Figure 3C). The results showed that the expression of circ_0058493 was significantly inhibited in K562/G01, while the expression of RHBDD1 mRNA wasn’t affected, excluding the off-target effect of sh-circ_0058493 (Figure 3D). We found that silencing circ_0058493 significantly increased the sensitivity of K562/G01 to imatinib, promoted apoptosis, and inhibited the proliferation of K562/G01 (Figures 3E,F,G). These results indicate that hsa_circ_0058493 promotes imatinib resistance in CML cell lines while silencing hsa_circ_0058493 inhibits imatinib-resistant cells development.

**FIGURE 4:**
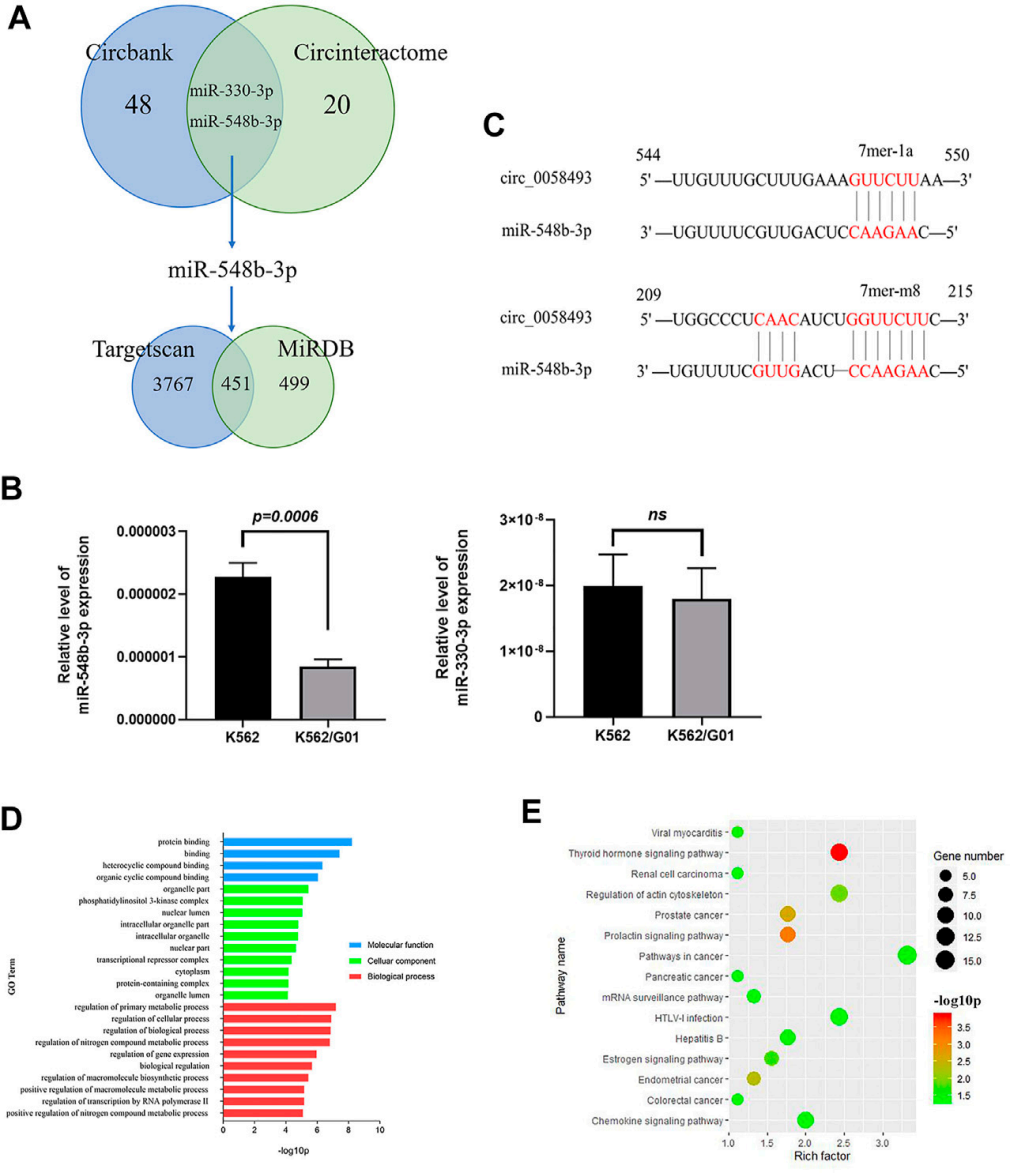
Hsa_circ_0058493 may involve in several physiological processes by interacting with miR-548b-3p. **(A)** Bioinformatics prediction of miRNA and target mRNA associated with hsa_circ_0058493. **(B)** The expression of two predicted miRNAs (miR-548b-3p and miR-330-3p) were detected in K562/G01 and K562 via RT-qPCR. The expression level of miR-548b-3p showed a significant difference (*p* = 0.0006). **(C)** There were two binding sites between miR-548b-3p and circ_0058493, which were located at 209-215 bp and 544-550 bp. **(D)** GO analysis of mRNA associated with hsa_circ_0058493/miR-548b-3p. **(E)** KEGG pathway analysis of mRNA associated with hsa_circ_0058493/miR-548b-3p. Quantitative data from three independent experiments are shown as the mean ± SD (error bars). ns: not statistically significant (Student’s *t*-test).

To investigate the mechanism by which circ_0058493 functions in CML cells, we conducted bioinformatics analysis. A total of 48 and 20 miRNAs bound to hsa_circ_0058493 were predicted by Circbank and Circinteractome online database respectively. Two potential miRNAs (miR-548b-3p and miR-330-3p) were predicted by the both databases (Figure 4A). We detected the expression of the two miRNAs in circ_0058493-upregulated K562/G01 and circ_0058493-downregulated K562 cells and found that miR-548b-3p significantly overexpressed in K562 cells compared with K562/G01 cells (*p* < 0.001) (Figure 4B). Further bioinformatics analysis revealed that there were two binding sites (209-215 bp and 544-550 bp) between miR-548b-3p and circ_0058493 (Figure 4C).

Furthermore, there were 3,767 and 499 target mRNAs bound to miR-548b-3p predicted by TargetScan and miRDB online database respectively, and 451 mRNAs were obtained after intersection (Figure 4A). Then we analyzed the relevant signal pathways of the 451 mRNAs. Gene Oncology (GO) and Kyoto Encyclopedia of Genes and Genomes (KEGG) Pathway analysis indicated that these differentially expressed mRNAs associated with circ_0058493/miR-548b-3p were relevant to several physiological processes, cellular components, molecular functions, and critical signaling pathways. (Figures 4D,E).

### Hsa_circ_0058493 in Exosome Derived From CML Cells Is Associated With Imatinib Resistance

**FIGURE 5:**
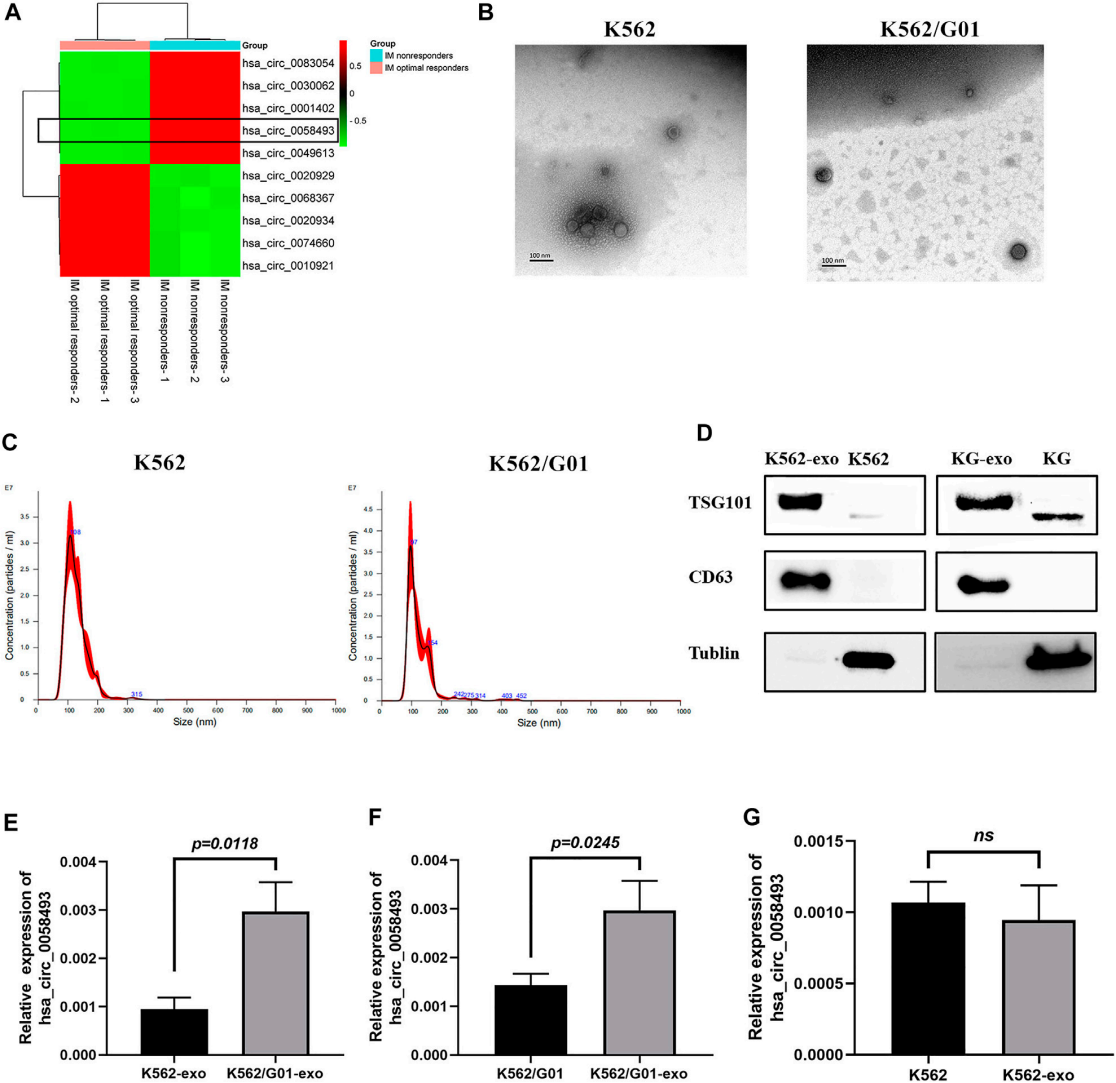
The expression level of hsa_circ_0058493 in exosomes is associated with imatinib resistance. **(A)**We selected top five upregulated and downregulated circRNAs (including circ58493) from the results of microarray analysis, normalized intensities of each selected circRNA were used to make the heat map. Hsa_circ_0058493 was framed to show that it was intensely overexpressed in plasma of IM nonresponders compared with IM optimal responders. **(B)** The exosomes isolated from K562/G01 and K562 culture medium by ultracentrifugation showed a typical cup shape under TEM. **(C)** The exosomes isolated from K562/G01 and K562 culture medium by ultracentrifugation showed diameters of most of the particles are 30-150 nm through NTA. **(D)** The exosomes isolated from K562/G01 (KG) and K562 culture medium expressed exosome marker CD63 and TSG101 analyzed by western blot. **(E)** Circ_0058493 in K562/G01 exosomes was significantly overexpressed by 3.14-fold compared with K562 exosomes (*p* = 0.0118). **(F)** The expression level of circ_0058493 in the exosomes derived from K562/G01 was increased by 2.08-fold compared with K562/G01 (*p* = 0.0245). **(G)** Circ_0058493 expression in exosomes derived from K562 showed no difference with K562. Quantitative data from three independent experiments are shown as the mean ± SD (error bars). ns: not statistically significant (Student’s *t*-test).

Recent studies have recommended that circRNAs in exosomes may serve as potential diagnosis or prognosis biomarkers (Li et al., 2020; Wu et al., 2020). In our present study, the results of the microarray analysis showed that the circ_0058493 in plasma of IM nonresponders was intensely overexpressed compared with IM optimal responders (Figure 5A). Then, we detected the expression level of circ_0058493 in exosomes derived from CML cell lines through RT-qPCR.

The exosomes isolated from K562/G01 and K562 supernatants using ultracentrifugation showed a typical cup shape determined by transmission electron microscope (TEM) (Figure 5B). The nanoparticle tracking analysis (NTA) results revealed that the diameters of most particles are in the range of 30–150 nm (Figure 5C). The presence of the exosome markers CD63 and TSG101 was confirmed by western blot (Figure 5D).

We found that circ_0058493 in K562/G01 exosomes was significantly overexpressed by 3.14-fold compared with K562 exosomes (*p* < 0.05) (Figure 5E). Besides, circ_0058493 was significantly enriched in the exosomes of K562/G01 compared with K562/G01 (Figure 5F), while the exosomes from K562 didn’t have this enrichment (Figure 5G). These data indicated that the expression level of circ_0058493 in exosomes derived from CML cell lines were associated with imatinib resistance.

## Discussion

As the frontline treatment of CML, imatinib has revolutionary changed the treatment outcome of CML patients (Jabbour, 2016). And considering the factors such as price and adverse reaction, imatinib is still the first choice for about 80% of newly diagnosed CML patients (Geelen et al., 2017). However, there are about 20% patients with CML discontinuing imatinib or changing to another drug due to treatment failure (Geelen et al., 2017). It’s urgent to clarify the mechanisms involved in the difference of CML clinical efficacy.

CircRNAs are a special novel type of endogenous noncoding RNA with covalently closed-loop structures, which makes them much more stable than liner RNA (Qu et al., 2015). Recent studies revealed that circRNAs were frequently deregulated in human cancers, and implicated in tumor initiation, progression, drug resistance (Wang et al., 2019; Jian et al., 2020; Wei et al., 2020), which had been appeared as promising biomarkers. However, the biological functions of circRNAs in CML are not well known. Using microarray analysis, we identified a novel oncogenic circRNA, hsa_circ_0058493, which was intensely overexpressed in PBMCs of IM nonresponders. Overexpression of circ_0058493 was associated with poor clinical efficacy of imatinib in CML patients. These results suggest that hsa_circ_0058493 in PBMCs is a stable biomarker for CML prognosis.

Studies have demonstrated the regulatory functions of circRNAs in cancer development by sponging miRNAs and affecting translation (Wei et al., 2020; Xie et al., 2020). In our study, downregulating the expression of circ_0058493 significantly enhance the sensitivity of K562/G01 to imatinib. Furthermore, we found that miR-548b-3p had two binding sites with circ-0058493 through bioinformatics analysis, and was significantly downregulated in CML resistant cells through RT-qPCR, which accorded with the regulation trend of circRNA and miRNA, so it deserved further study. Besides, the relationships among circ-0058493, miR-548b-3p and the mRNAs analyzed by GO and KEGG also need further investigation and verification.

Multiple studies had depicted that circRNAs in exosomes served as novel important biomarkers since the circRNAs related to tumor progression or drug resistance could be horizontally transmitted, thus interfering with the biological functions of adjacent or distant recipient cells, and detected in body fluids of patients (Tang et al., 2018; Fan et al., 2019; Xu X. et al., 2020; Wu et al., 2020; Xie et al., 2020). However, whether circRNAs encapsulated and transmitted by exosomes could change the biological functions of CML cells has not been explored. In our study, circ_0058493 was also found enriched in the exosomes derived from imatinib-resistant K562/G01 cells, indicating exosomal circ_0058493 was associated with imatinib resistance in CML. However, further studies are needed to clarify the role of exosomal circ_0058493 in CML resistance, and to figure out the relationship between circ_0058493 level in exosome and the clinical efficacy of CML patients.

Many studies have shown that circRNAs are significantly enriched in exosomes compared with cells (Xu J. et al., 2020; Huang et al., 2020). Xu et al. found that circRNA-SORE was substantially more enriched in exosomes from the culture media of sorafenib-resistant cells than in the cell cytosol (Xu J. et al., 2020). Therefore, when it comes to detecting the level of circRNAs in clinical samples for prediction or prognosis, by increasing the detection sensitivity, it would be an advantage to use exosomes rather than patients’ plasma, or cells. But at the same time, it is an additional extra time-consuming step since exosomes extraction is a complex process. In comparison, the levels of circRNAs in plasma or plasma cells are much easier to obtain. As the result, the levels of circRNAs in exosomes, plasma, or cells may play different roles in clinical applications for different purposes.

Although we have identified hsa_circ_0058493 as a promoter in CML resistance, there are several aspects to be improved. Firstly, it would be better to conduct high-throughput human circRNA microarray individually using plasma samples from each patient (6 plasma samples from IM optimal responders used as non-resistant groups, six plasma samples from IM nonresponders used as resistant groups), because of the individual differences among patients. In our study, we mixed the plasma samples due to the limited plasma volume obtained from patients. Some studies also conducted circRNAs sequencing using mixed samples (Wan et al., 2019; Li et al., 2020; Xie et al., 2020). For example, Xie et al. identified circSHKBP1 through RNA-sequencing using 8 GC tissues and one mixed sample of eight matched adjacent normal tissues (Xie et al., 2020). Wan et al. mixed the serum of 10 healthy individuals and extracted the exosomes, which were used as a control, to figure out the functional significance of K562-derived exosomes in adipose tissue (Wan et al., 2019). Secondly, we just revealed the potential regulatory mechanisms of circ_0058493 by bioinformatic analysis and found the overexpressed miR-548b-3p. We need to further identify whether circ_0058493 could interact with miR-548b-3p and found the exact mRNAs regulated by circ_0058493. Besides, we only detected the expression level of hsa_circ_0058493 in exosomes derived from CML cell lines. To investigate the relevance between the clinical efficacy of CML patients and exosomal hsa_circ_0058493, we need to further test the exact expression level of hsa_circ_0058493 in the plasma exosomes of CML patients. The results from microarray analysis showed that hsa_circ_0058493 in plasma of IM nonresponders was intensively overexpressed compared with IM optimal responders. It has been demonstrated that serum circRNAs could be stably encapsulated in exosomes and may serve as a potential biomarker for cancer detection (Li et al., 2015). Therefore, we assumed that the potential association between the expression level of circ_0058493 in plasma exosomes and the clinical efficacy of CML patients was worthy of further exploring.

In conclusion, we demonstrated that high level of hsa_circ_0058493 in the peripheral blood mononuclear cells of CML patients was associated with poor imatinib clinical efficacy. Downregulating the expression of circ_0058493 could intensively enhanced the sensitivity of imatinib-resistant cells. More importantly, hsa_circ_0058493 was significantly enriched in the exosomes of imatinib-resistant CML cell lines. Consequently, hsa_circ_0058493 in PBMCs was considered as a potential prognostic biomarker and might provide a therapeutic target for CML treatment.

## Data Availability

The original contributions presented in the study are included in the article/Supplementary Material, further inquiries can be directed to the corresponding authors.

## Abbreviations

ABL, Abelson murine leukemia viral oncogene homolog; 7-AAD, 7-aminoactinomycin D; BCR, breakpoint cluster region; BMI, body mass index; CCK-8, cell counting kit-8; CML, chronic myeloid leukemia; circRNAs, circular RNAs; cRNAs, complementary DNA; EdU, 5-ethynyl-2′-deoxyuridine; EVs, extracellular vesicles; FBS, fetal bovine serum; GO, gene oncology; GC, gastric cancer; gDNA, genomic DNA; IM, imatinib; KEGG, kyoto encyclopedia of genes and genomes; miRNAs, microRNAs; NTA, nanoparticle tracking analysis; PE, Phycoerythrin; PBS, Phosphate buffer solution; Ph + CML-CP, Philadelphia chromosome positive CML chronic phase; PBMCs, Peripheral Blood Mononuclear Cells; Rnase R, Ribonuclease R; RHBDD1, Rhomboid domain containing 1; RT-qPCR: RNA preparation and quantitative real-time PCR; shRNA, short hairpin RNA; sh-NC, short hairpin-negative control; TEM, Transmission Electron Microscope; TKIs, Tyrosine Kinase Inhibitors; TSG101, Tumor Susceptibility 101.
