# Supplementary figures and images for: CircRNA Microarray Profiling Reveals hsa_circ_0058493 as a Novel Biomarker for Imatinib-Resistant CML
Source: Front Pharmacol. 2021 Sep 13;12:728916. doi: 10.3389/fphar.2021.728916 (PMC8473700; doi:10.3389/fphar.2021.728916)

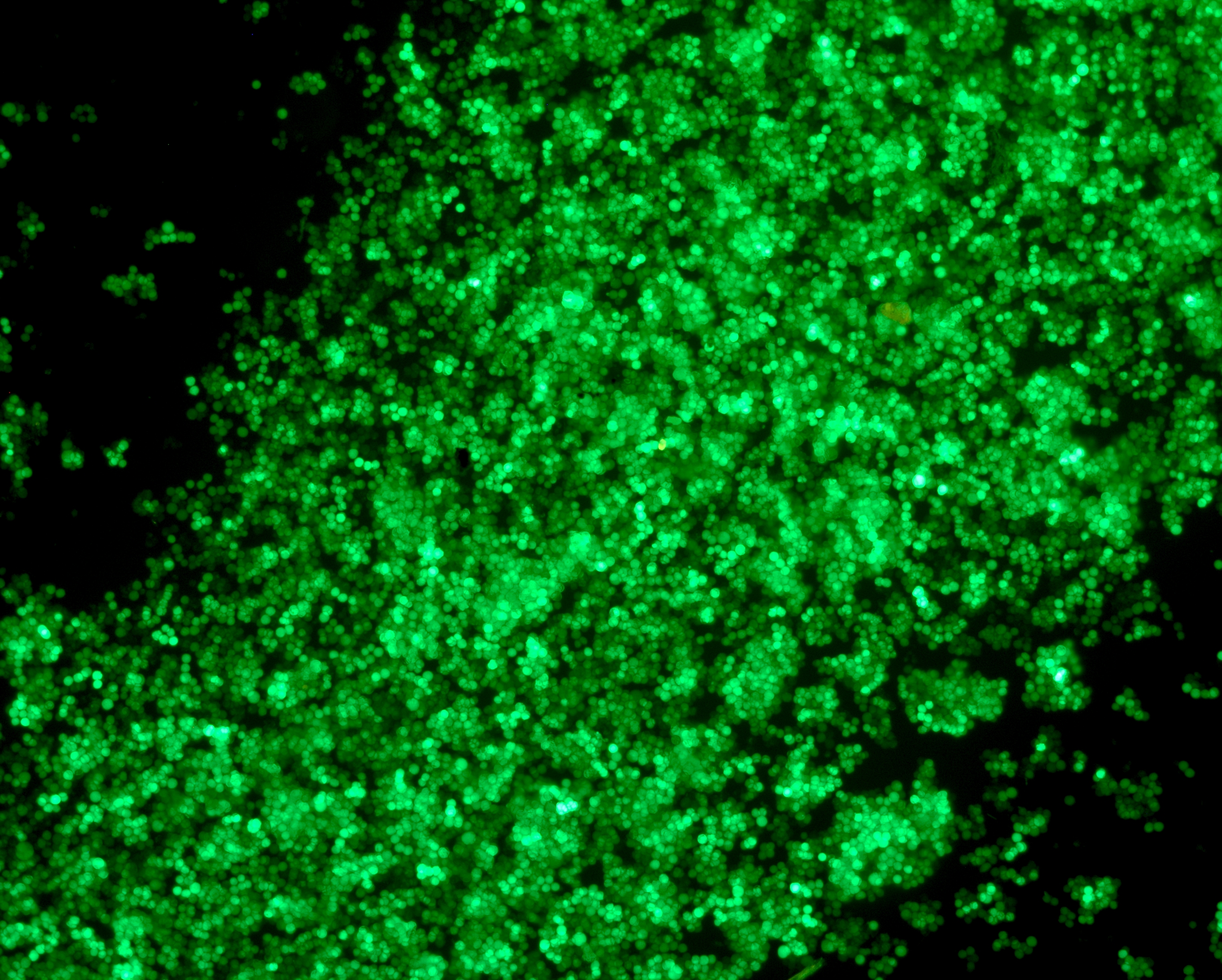

Supplement: Supplementary file 1 [file DataSheet3.ZIP › Figure 3/Figure3C sh-circ58493-1.jpg]

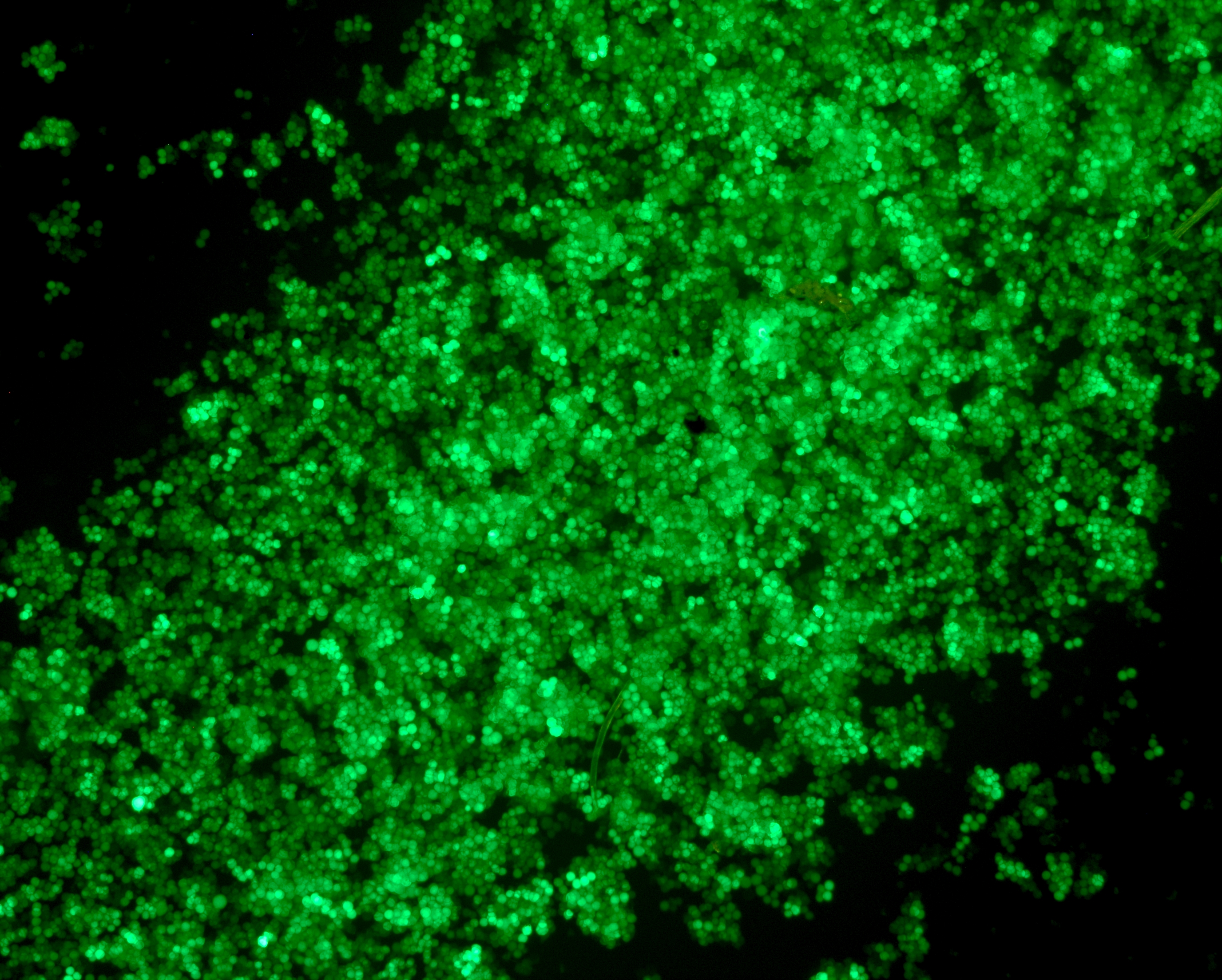

Supplement: Supplementary file 1 [file DataSheet3.ZIP › Figure 3/Figure3C sh-circ58493-2.jpg]

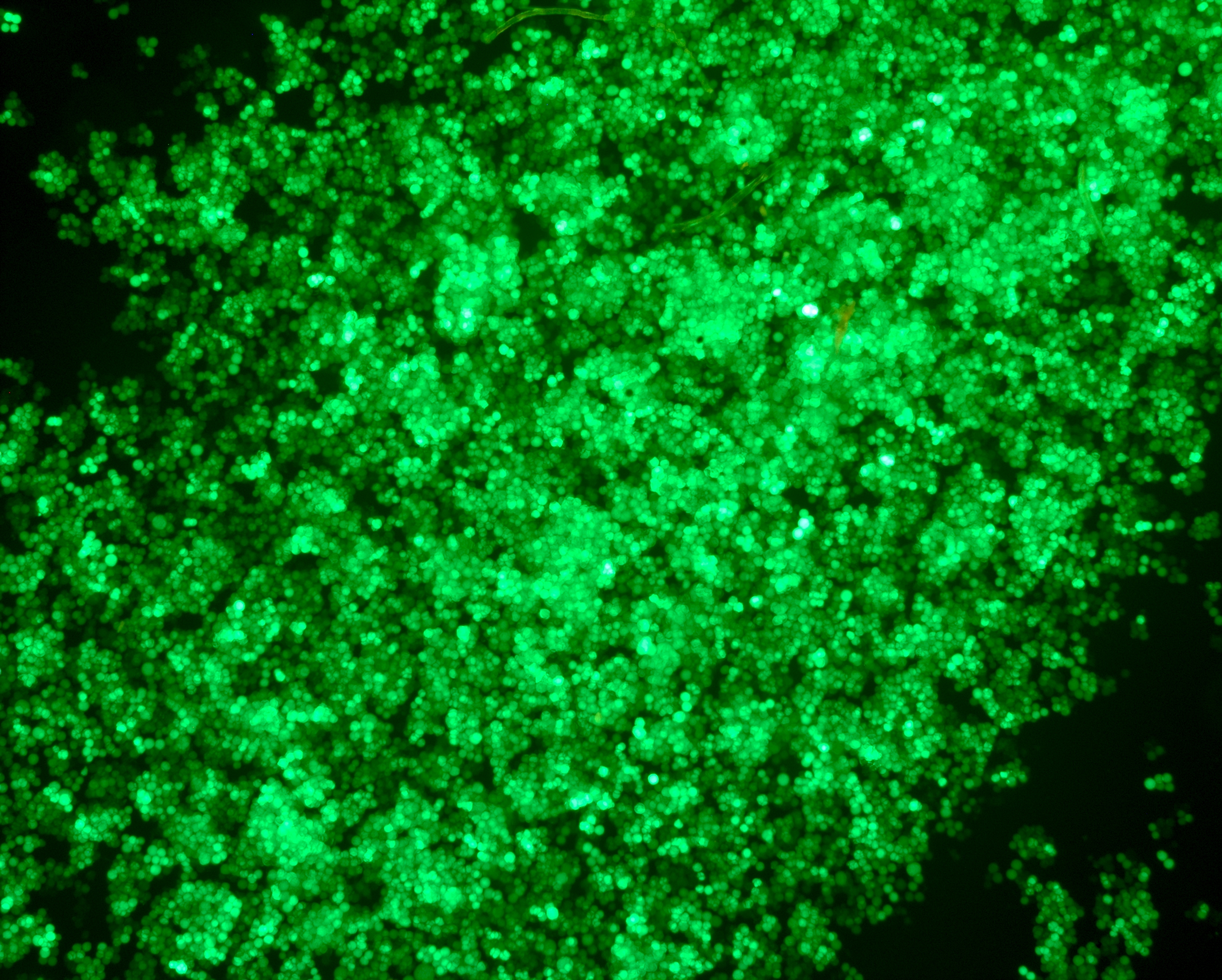

Supplement: Supplementary file 1 [file DataSheet3.ZIP › Figure 3/Figure3C sh-circ58493-3.jpg]

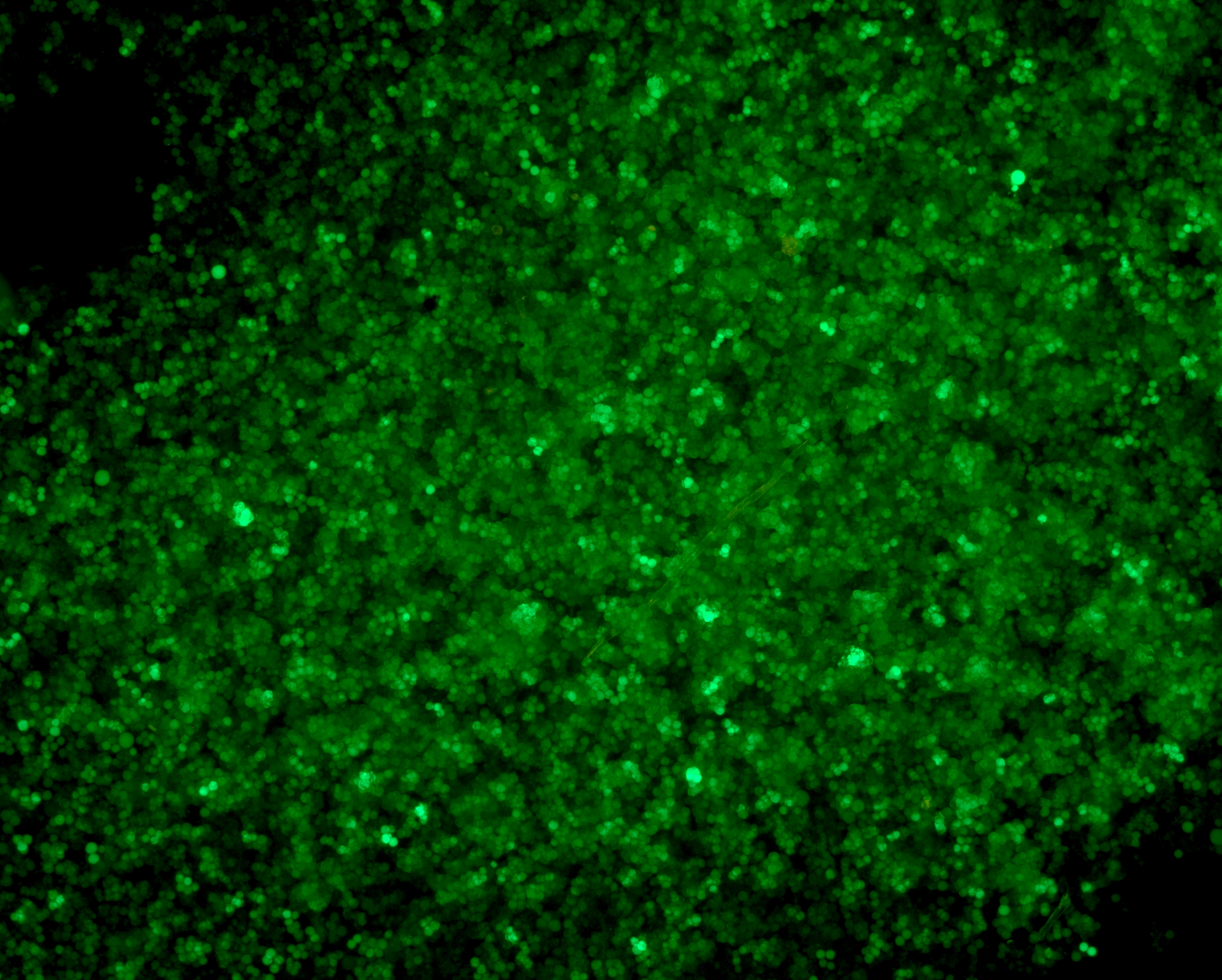

Supplement: Supplementary file 1 [file DataSheet3.ZIP › Figure 3/Figure3C sh-NC.jpg]

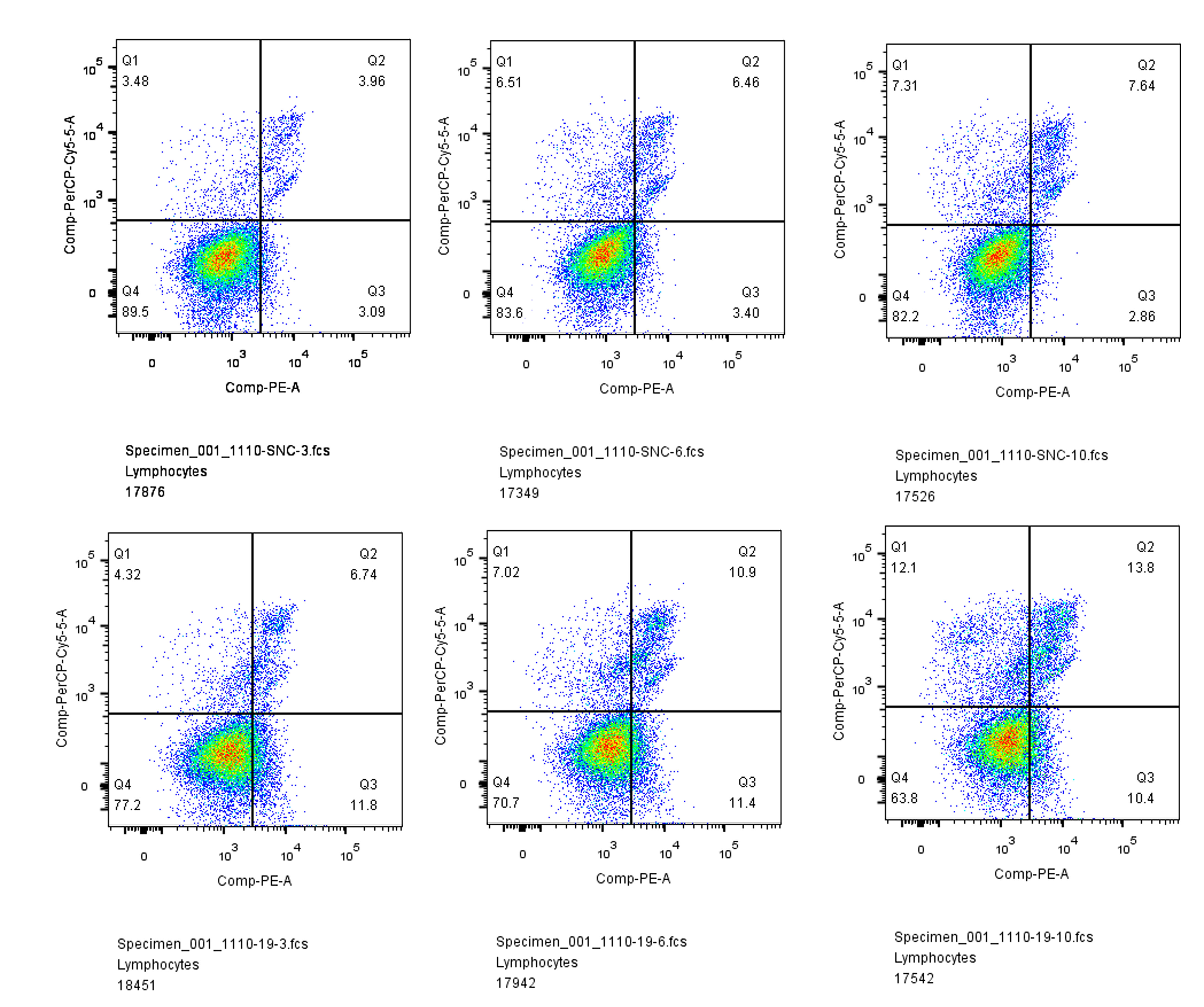

Supplement: Supplementary file 1 [file DataSheet3.ZIP › Figure 3/Figure3F.jpg]

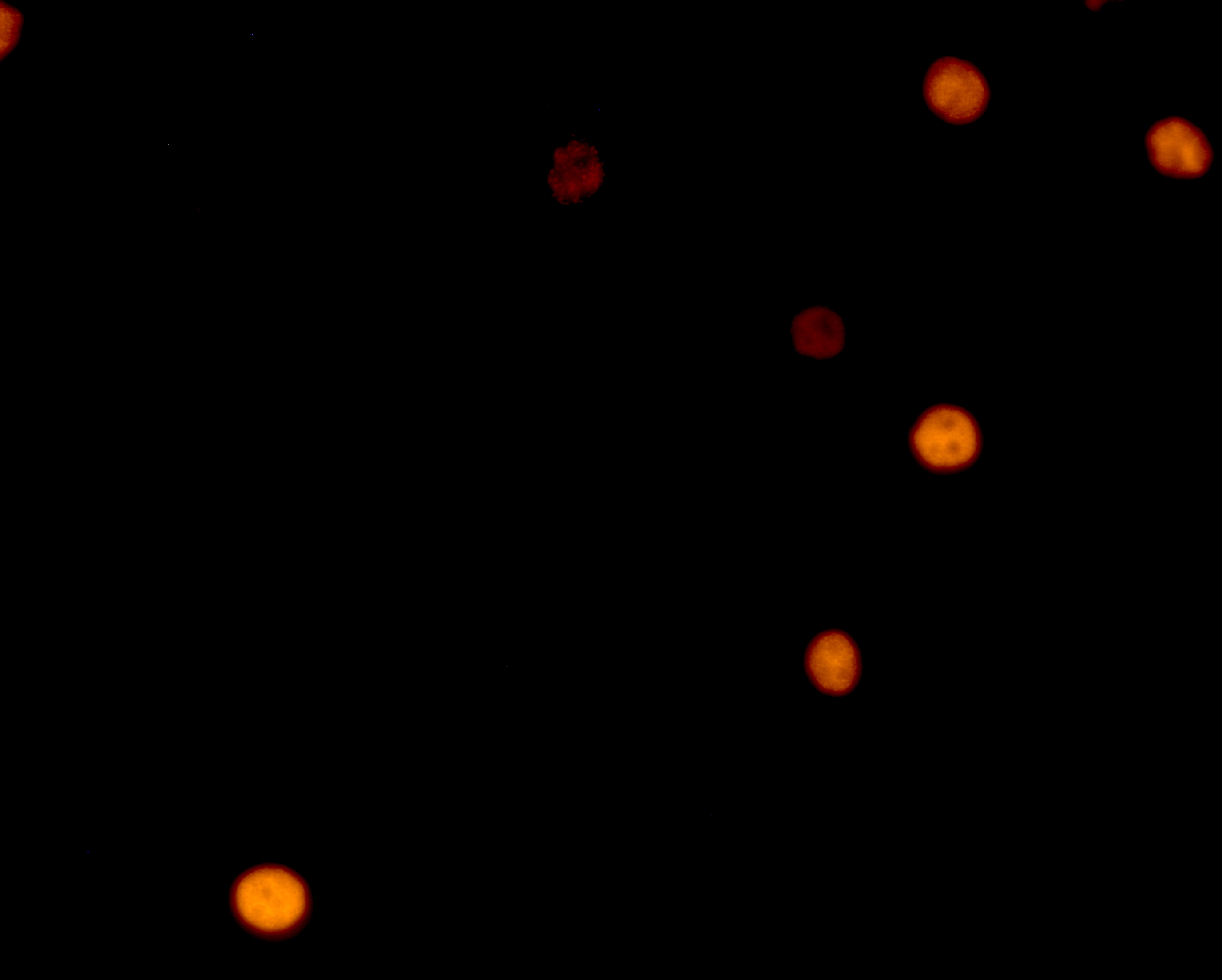

Supplement: Supplementary file 1 [file DataSheet3.ZIP › Figure 3/Figure3G sh-circ58493-2 Edu-1.jpg]

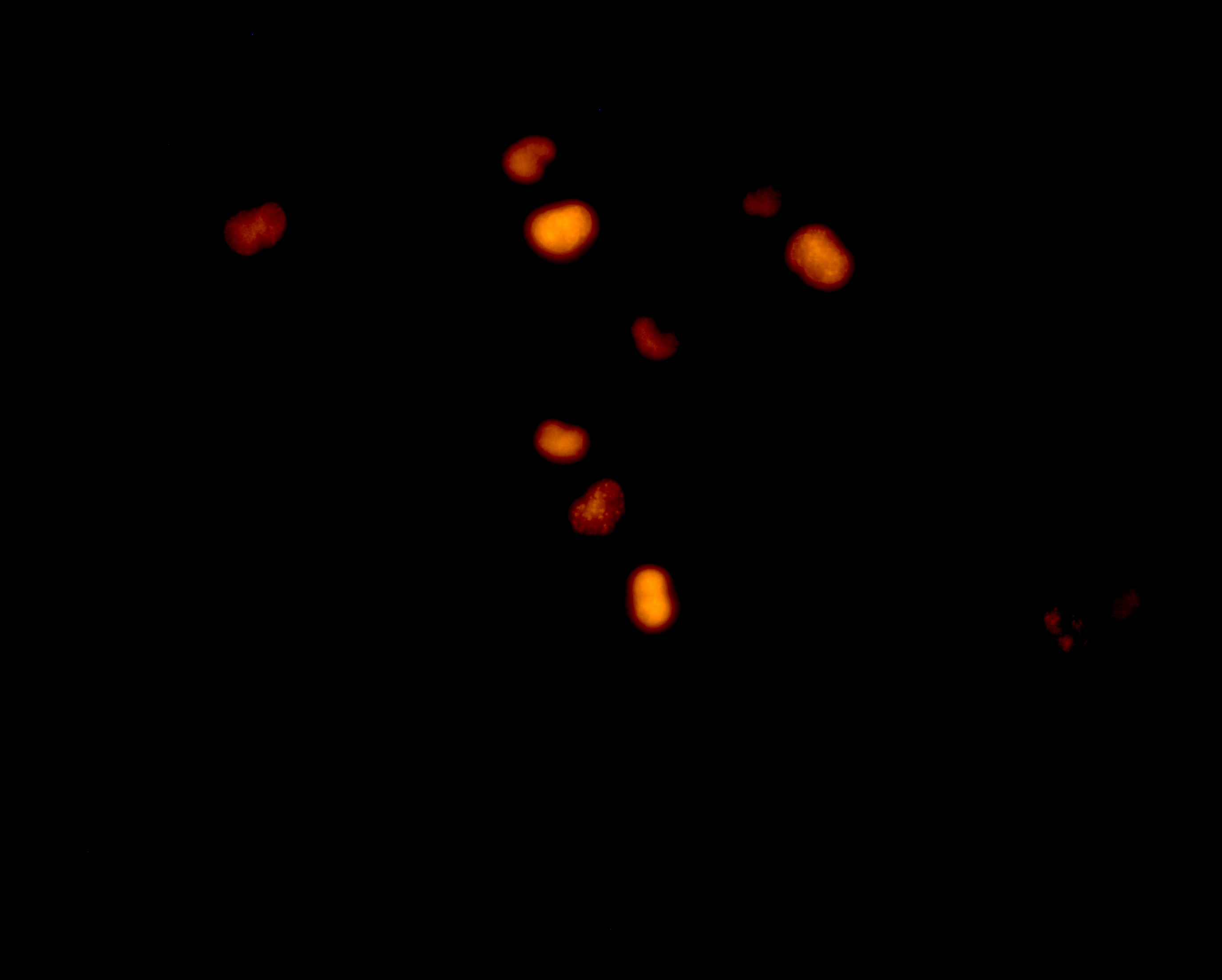

Supplement: Supplementary file 1 [file DataSheet3.ZIP › Figure 3/Figure3G sh-circ58493-2 Edu-2.jpg]

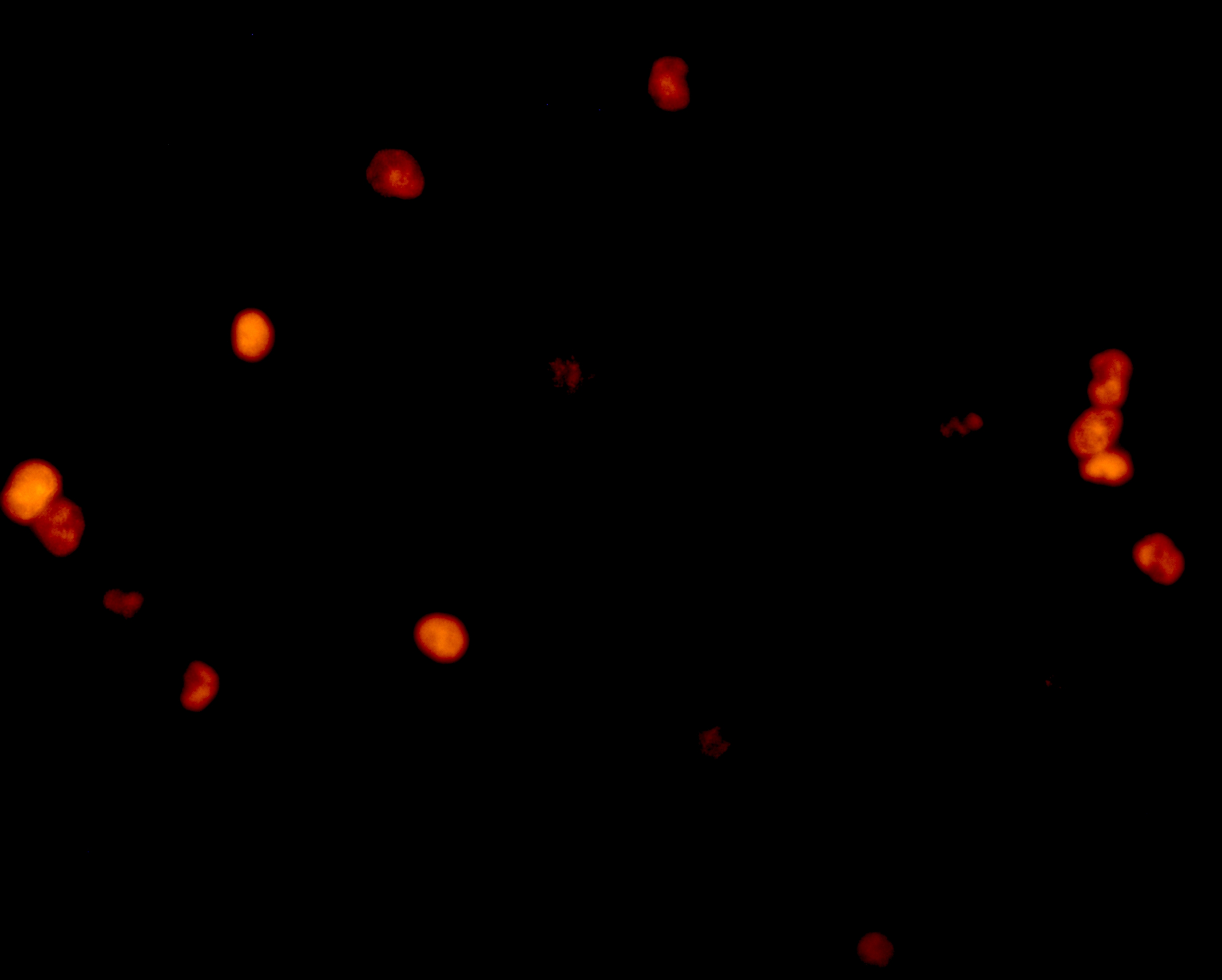

Supplement: Supplementary file 1 [file DataSheet3.ZIP › Figure 3/Figure3G sh-circ58493-2 Edu-3.jpg]

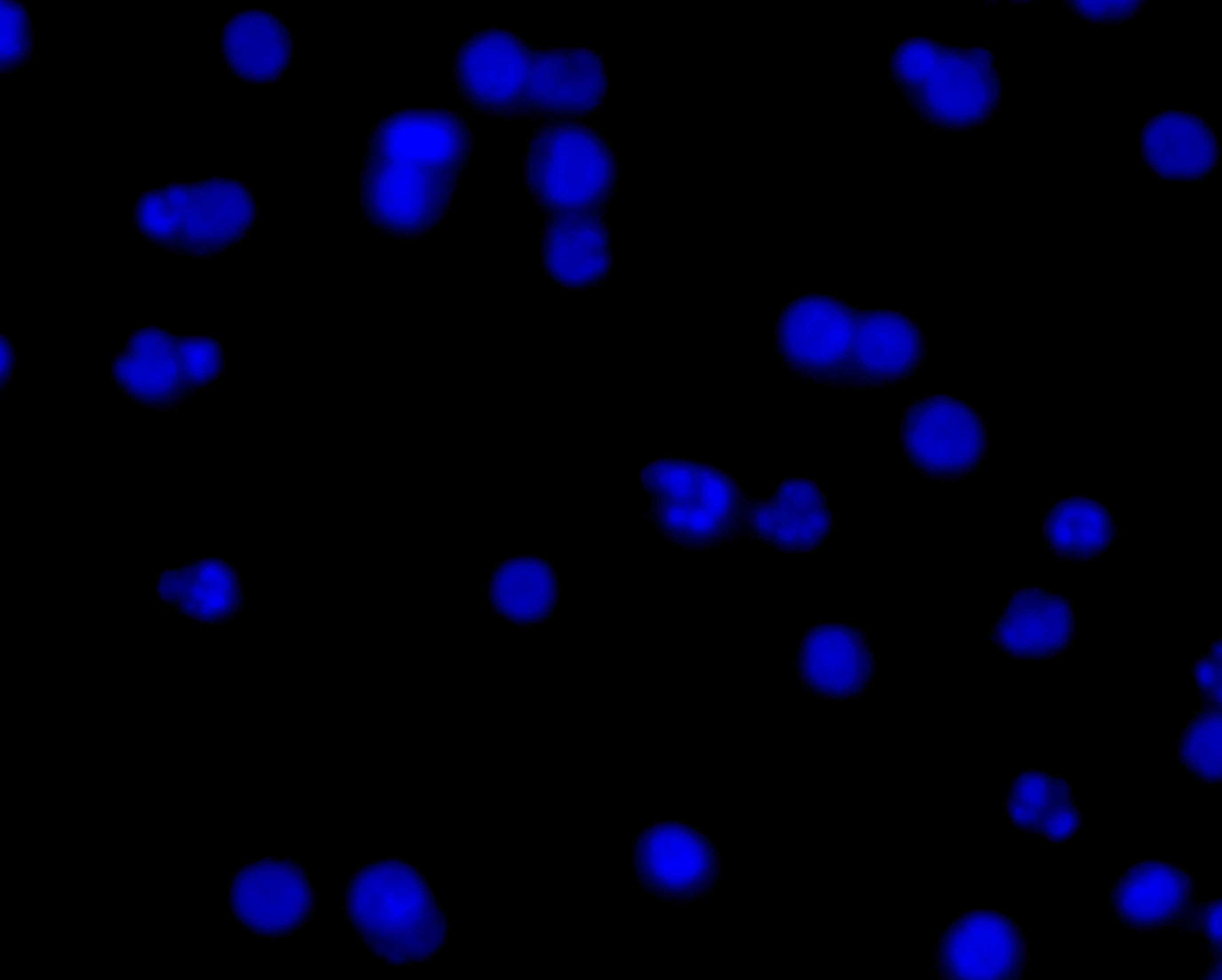

Supplement: Supplementary file 1 [file DataSheet3.ZIP › Figure 3/Figure3G sh-circ58493-2 Hochest-1.jpg]

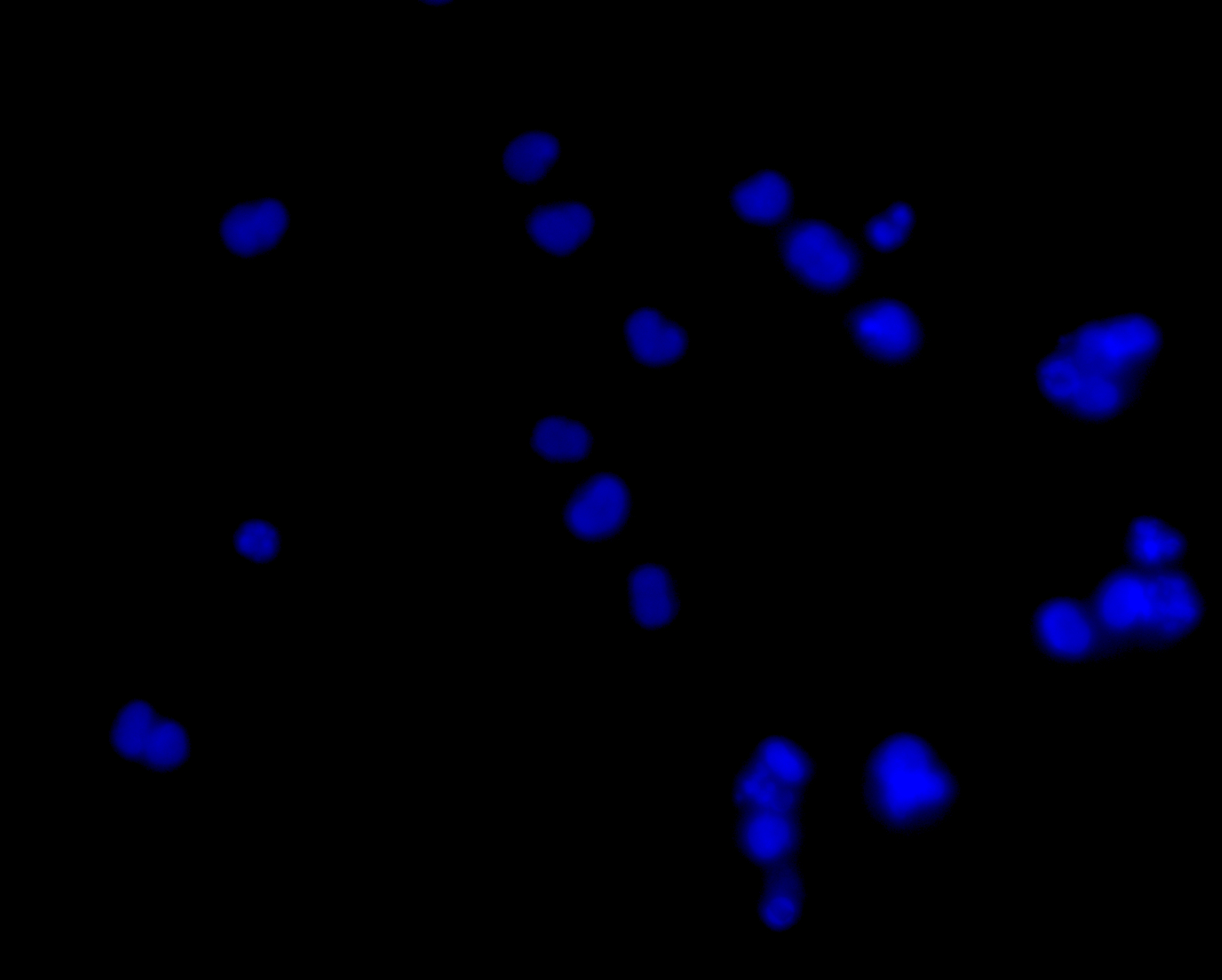

Supplement: Supplementary file 1 [file DataSheet3.ZIP › Figure 3/Figure3G sh-circ58493-2 Hochest-2.jpg]

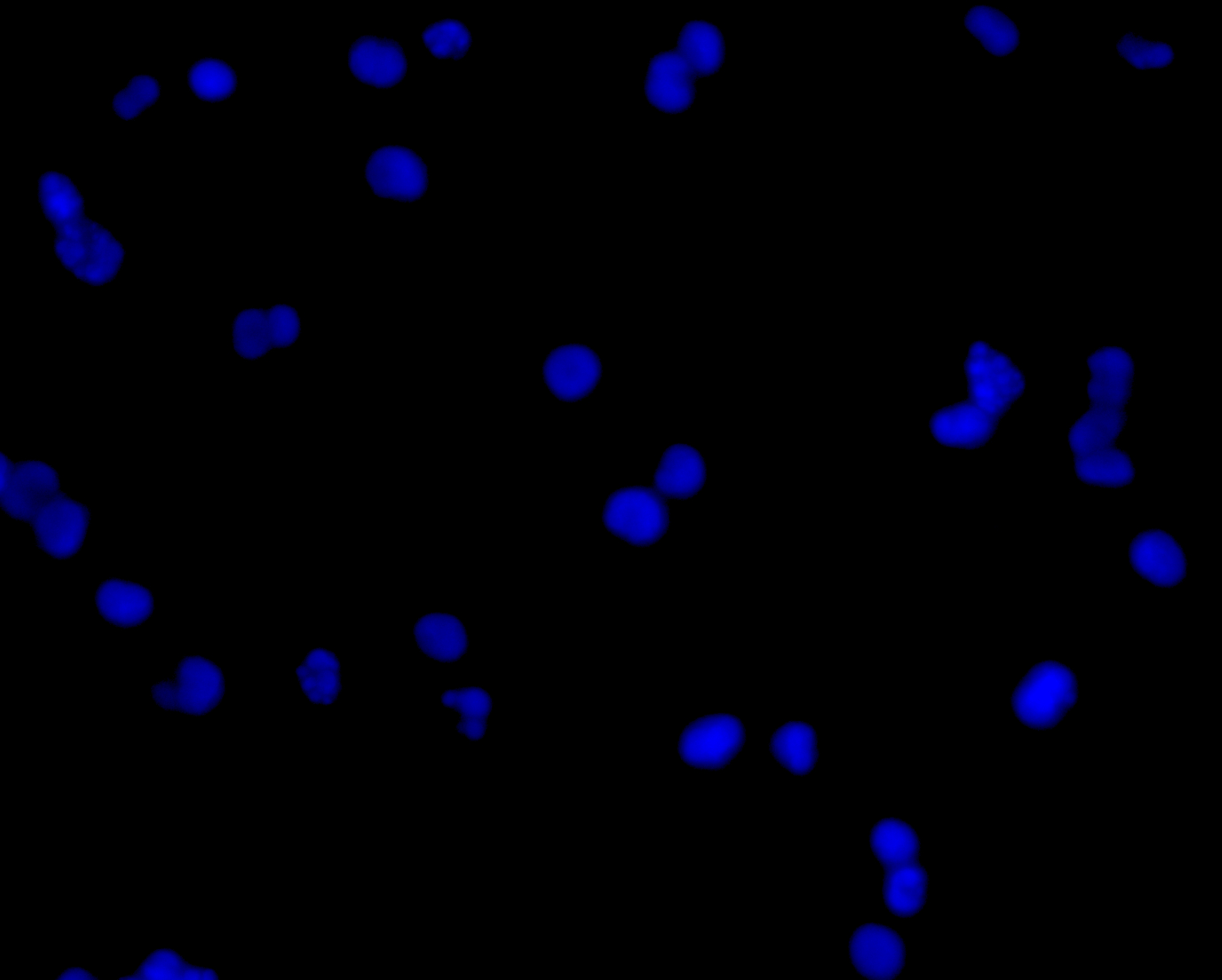

Supplement: Supplementary file 1 [file DataSheet3.ZIP › Figure 3/Figure3G sh-circ58493-2 Hochest-3.jpg]

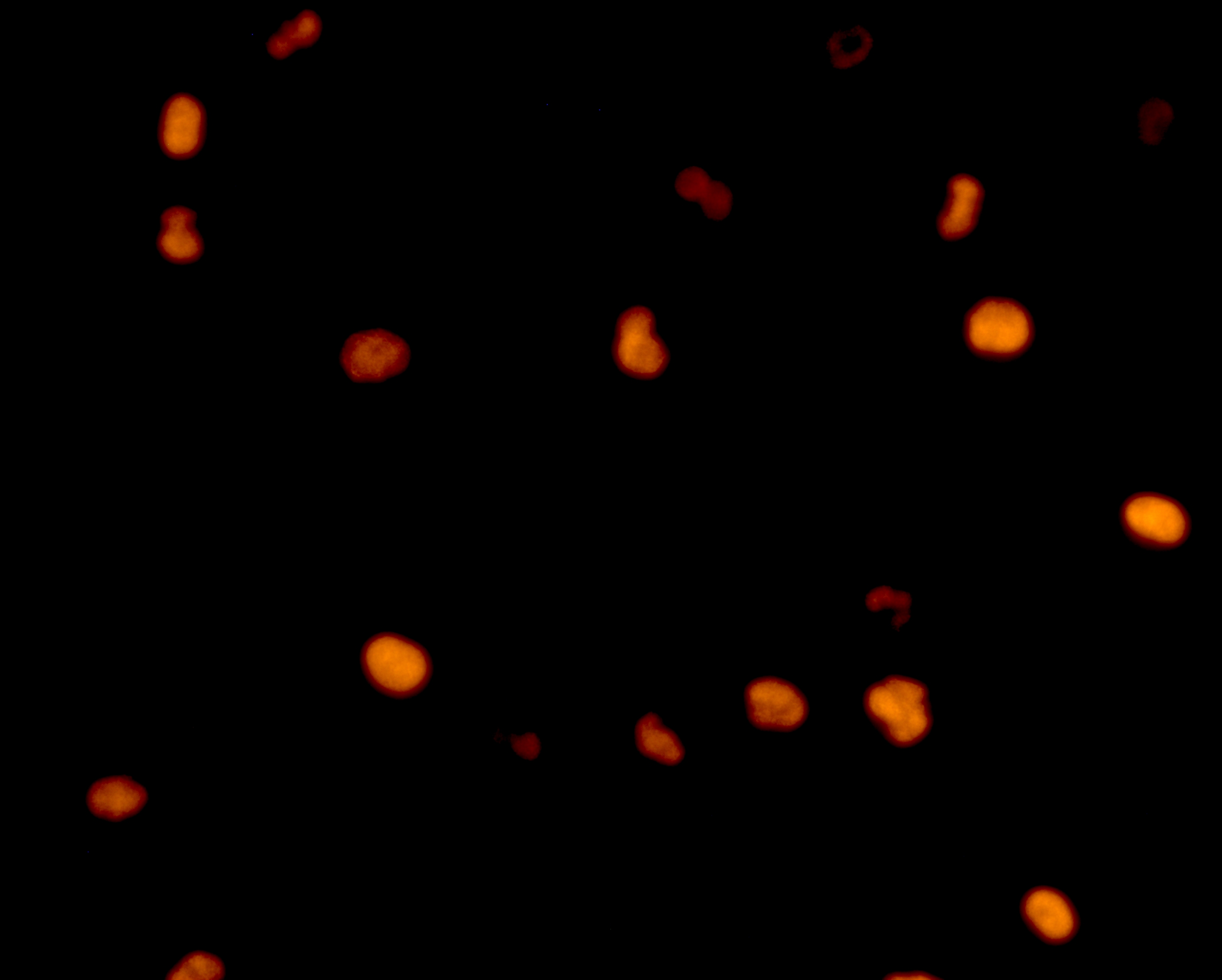

Supplement: Supplementary file 1 [file DataSheet3.ZIP › Figure 3/Figure3G-SNC- Edu-1.jpg]

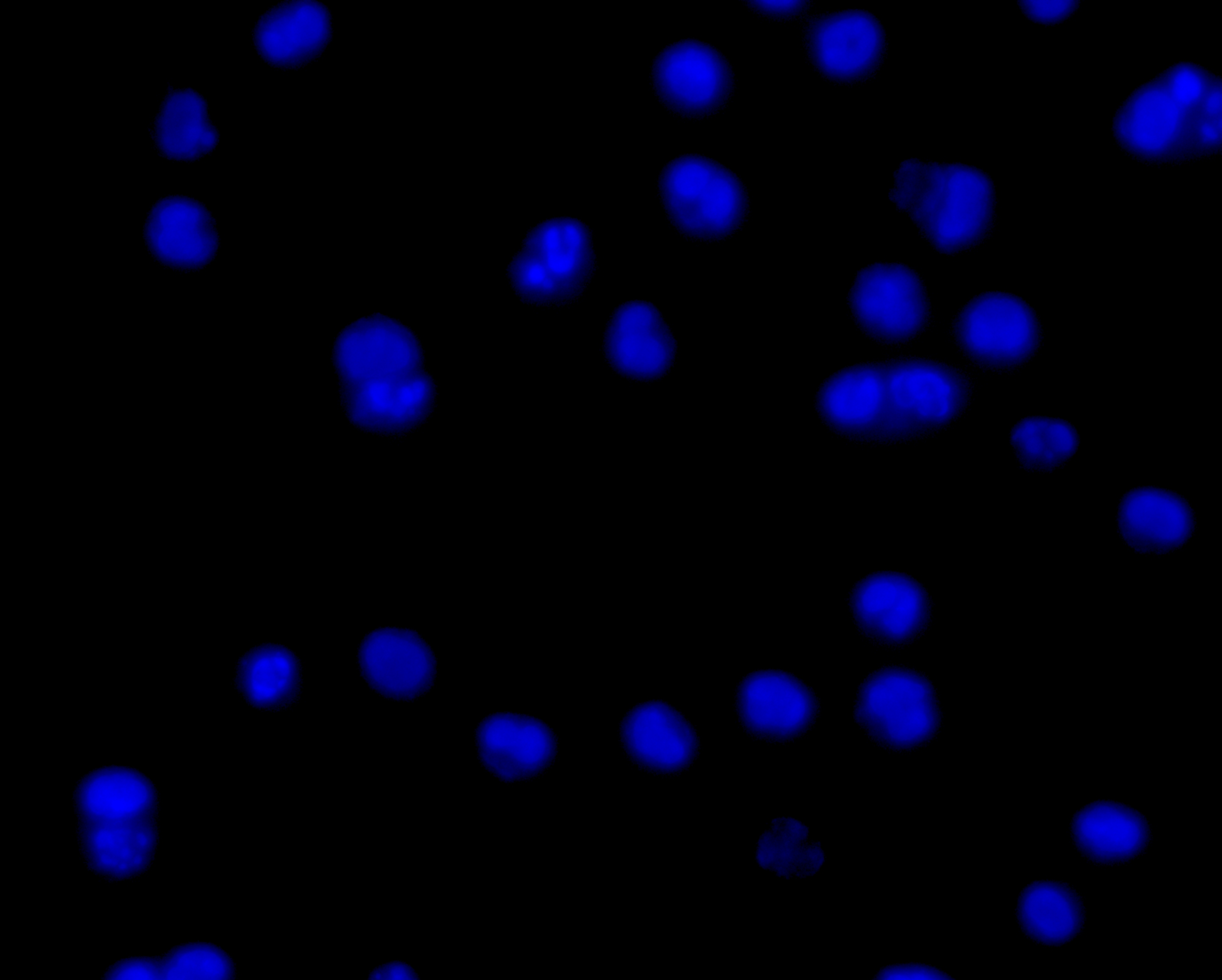

Supplement: Supplementary file 1 [file DataSheet3.ZIP › Figure 3/Figure3G-SNC- Hochest-1.jpg]

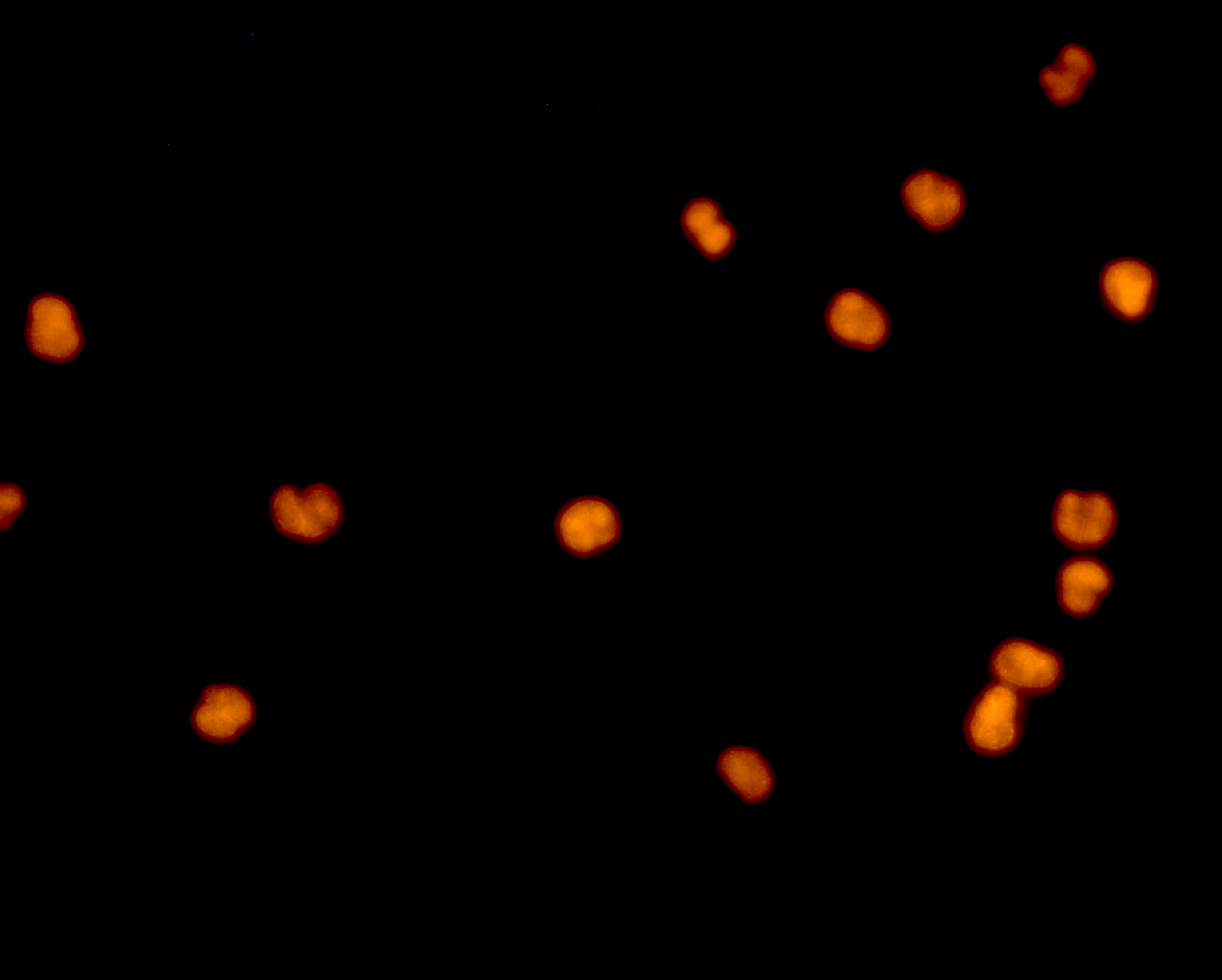

Supplement: Supplementary file 1 [file DataSheet3.ZIP › Figure 3/Figure3G-SNC-Edu-2.jpg]

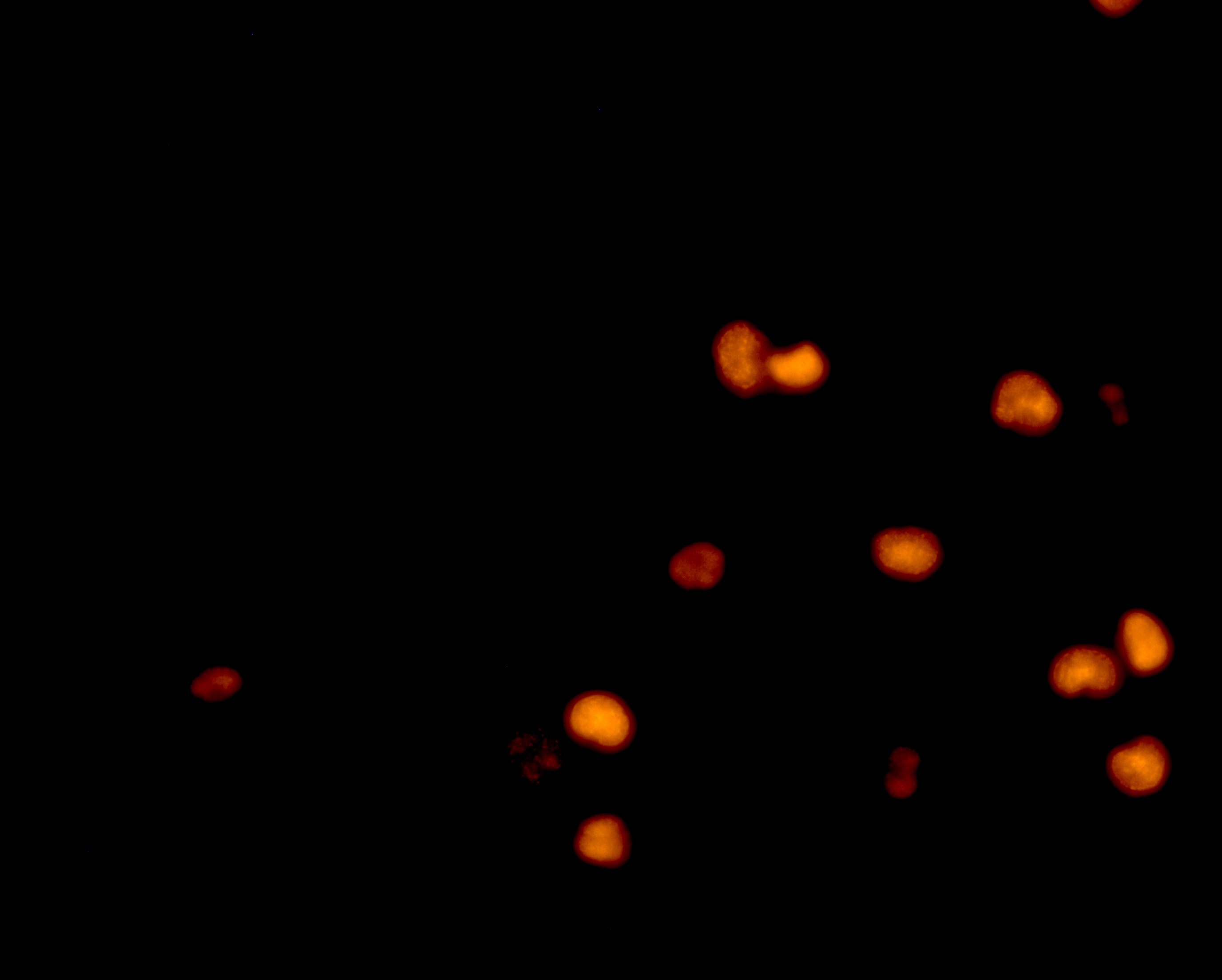

Supplement: Supplementary file 1 [file DataSheet3.ZIP › Figure 3/Figure3G-SNC-Edu-3.jpg]

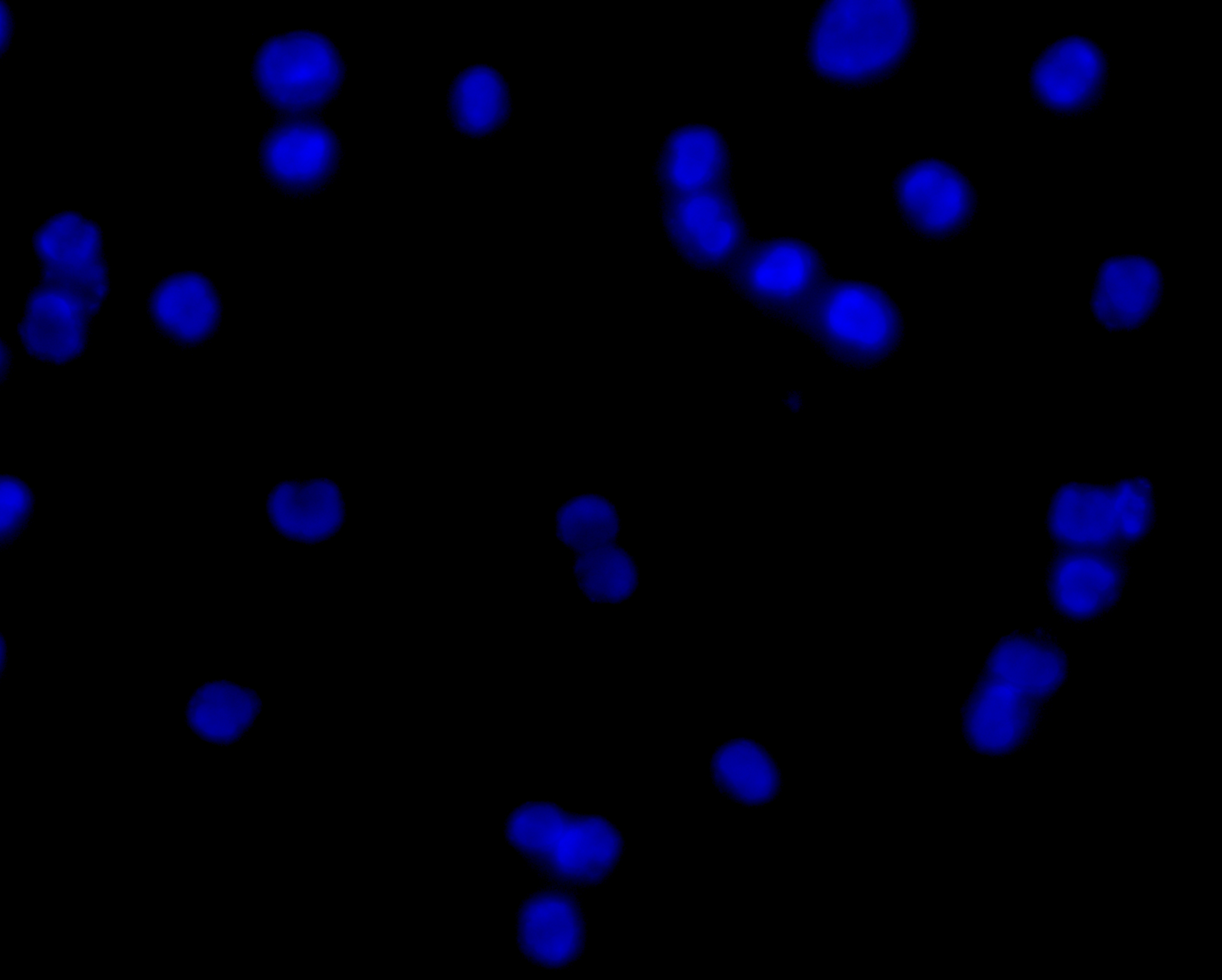

Supplement: Supplementary file 1 [file DataSheet3.ZIP › Figure 3/Figure3G-SNC-Hochest-2.jpg]

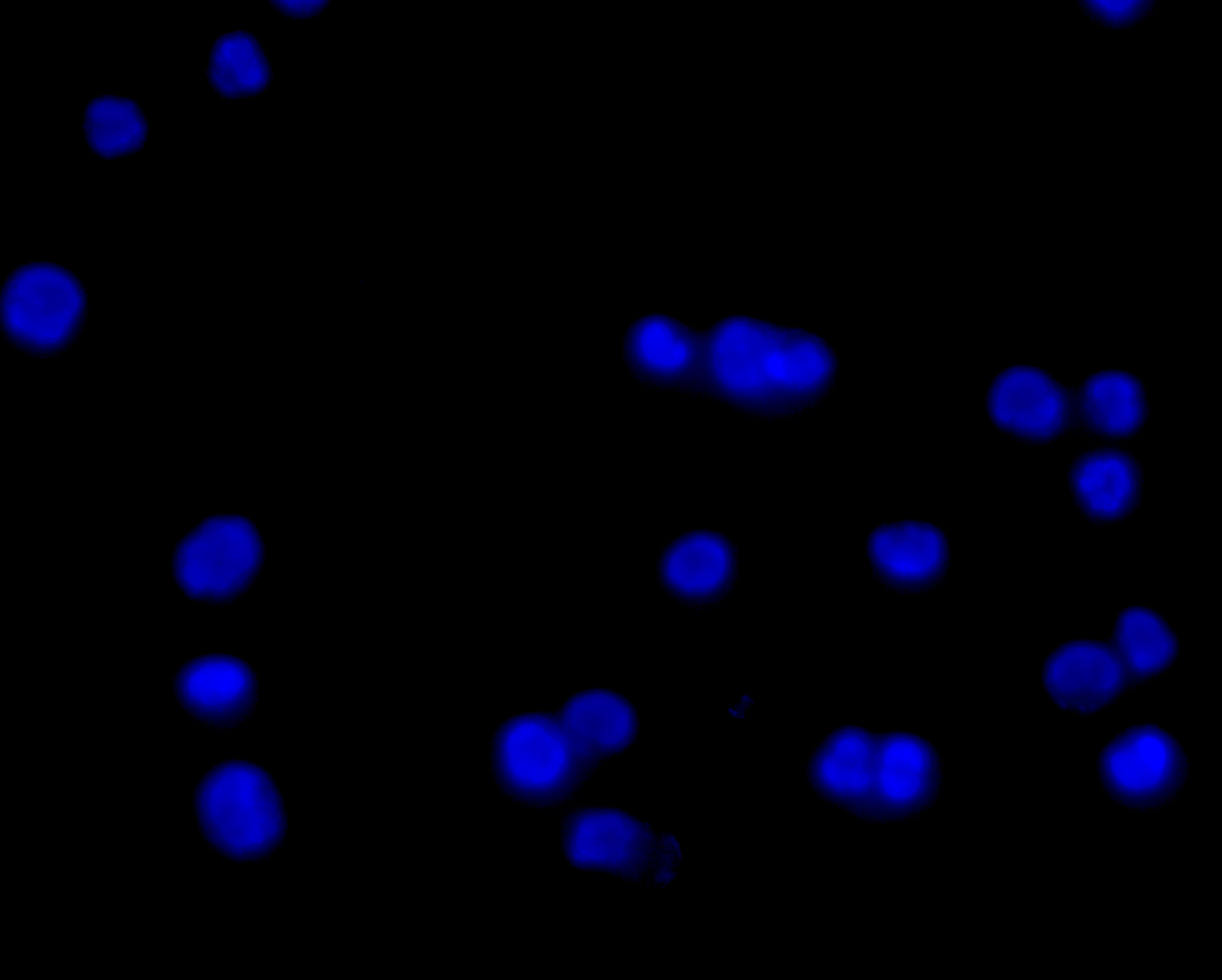

Supplement: Supplementary file 1 [file DataSheet3.ZIP › Figure 3/Figure3G-SNC-Hochest-3.jpg]

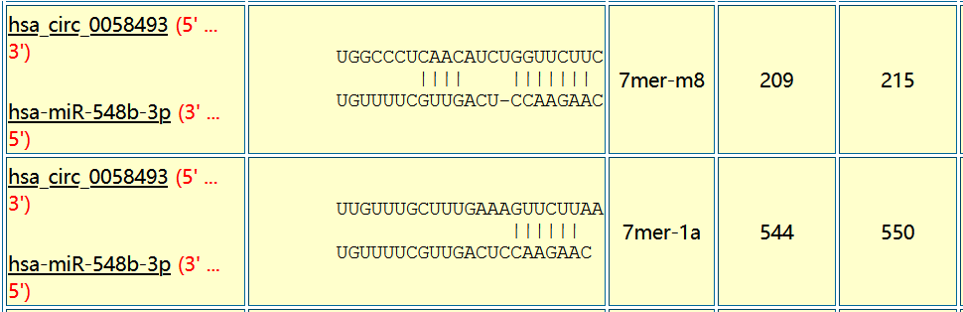

Supplement: Supplementary file 2 [file DataSheet4.ZIP › Figure 4/Figure 4C.png]

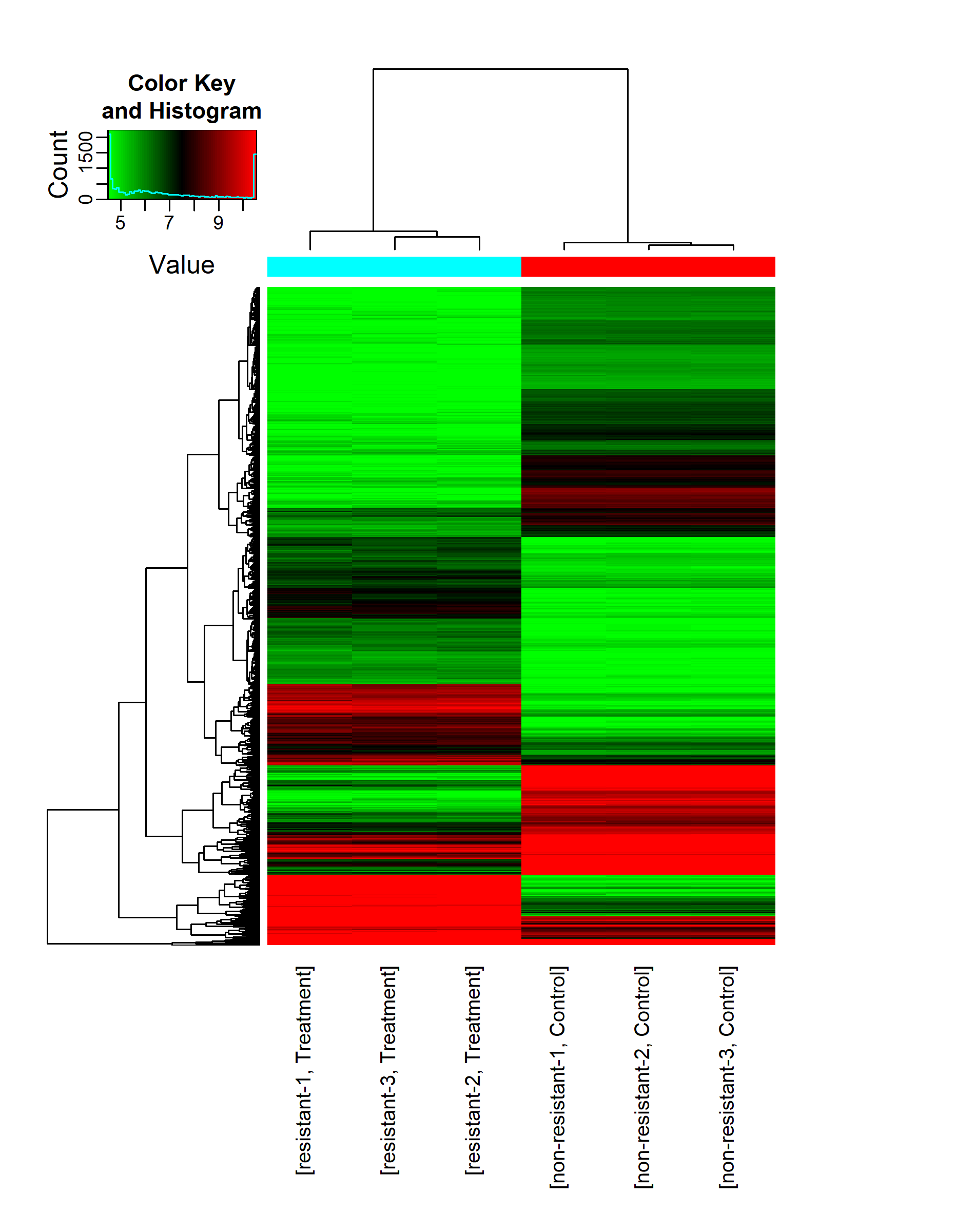

Supplement: Supplementary file 3 [file DataSheet1.ZIP › Figure 1/heatmap_of_Treatment_vs_Control.2.0.0.05.unpaired.tif]

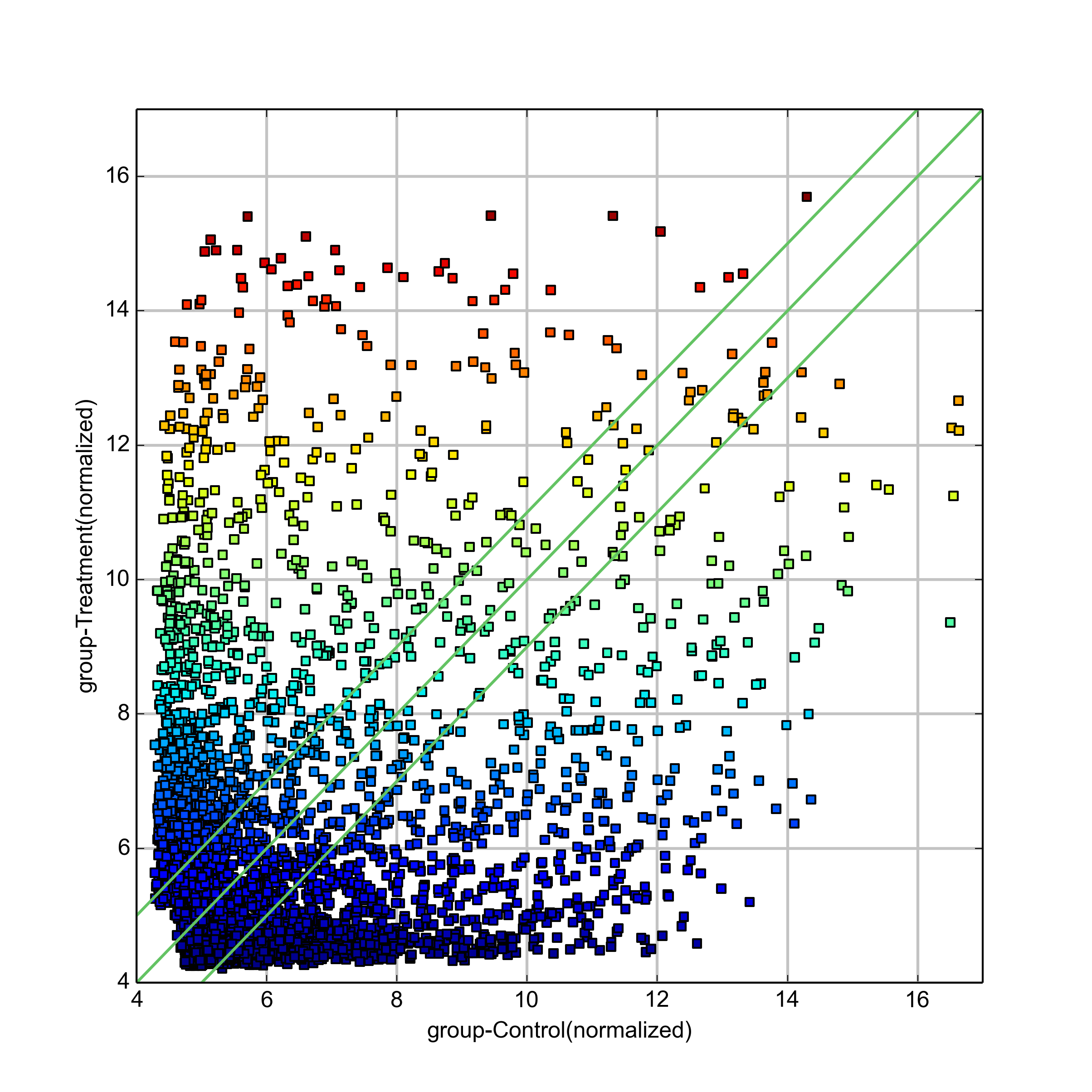

Supplement: Supplementary file 3 [file DataSheet1.ZIP › Figure 1/unpaired_Treatment_vs_Control.2.0.0.05.scatter.png]

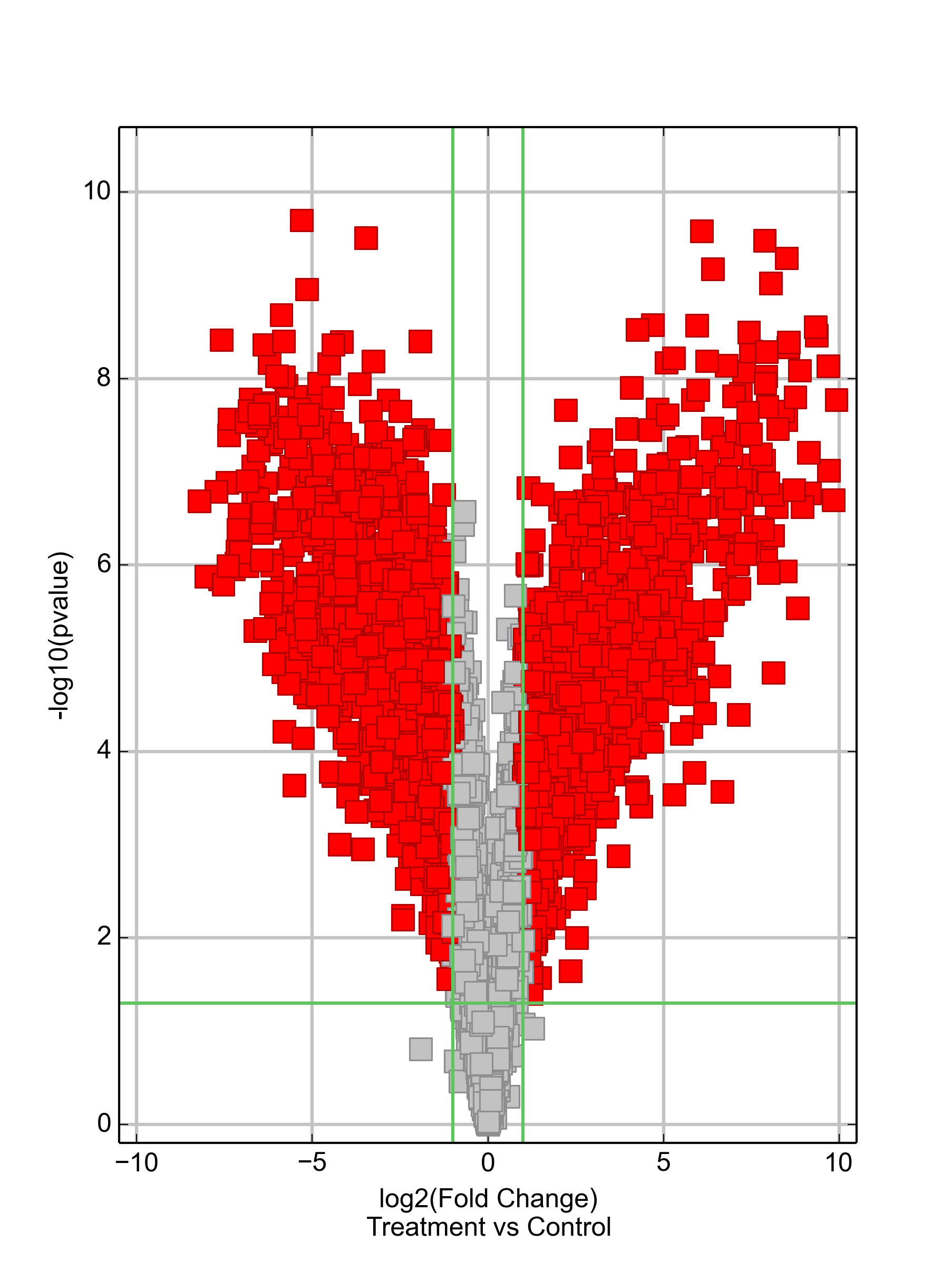

Supplement: Supplementary file 3 [file DataSheet1.ZIP › Figure 1/volcano_plot_unpaired_Treatment_vs_Control.png]

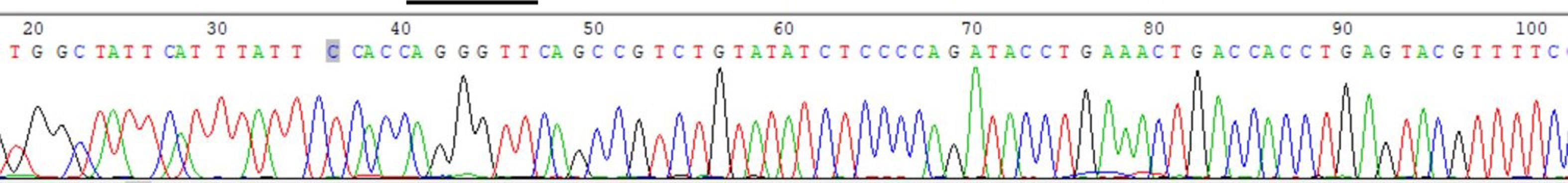

Supplement: Supplementary file 5 [file DataSheet2.ZIP › Figure 2/Figure 2E.JPG]

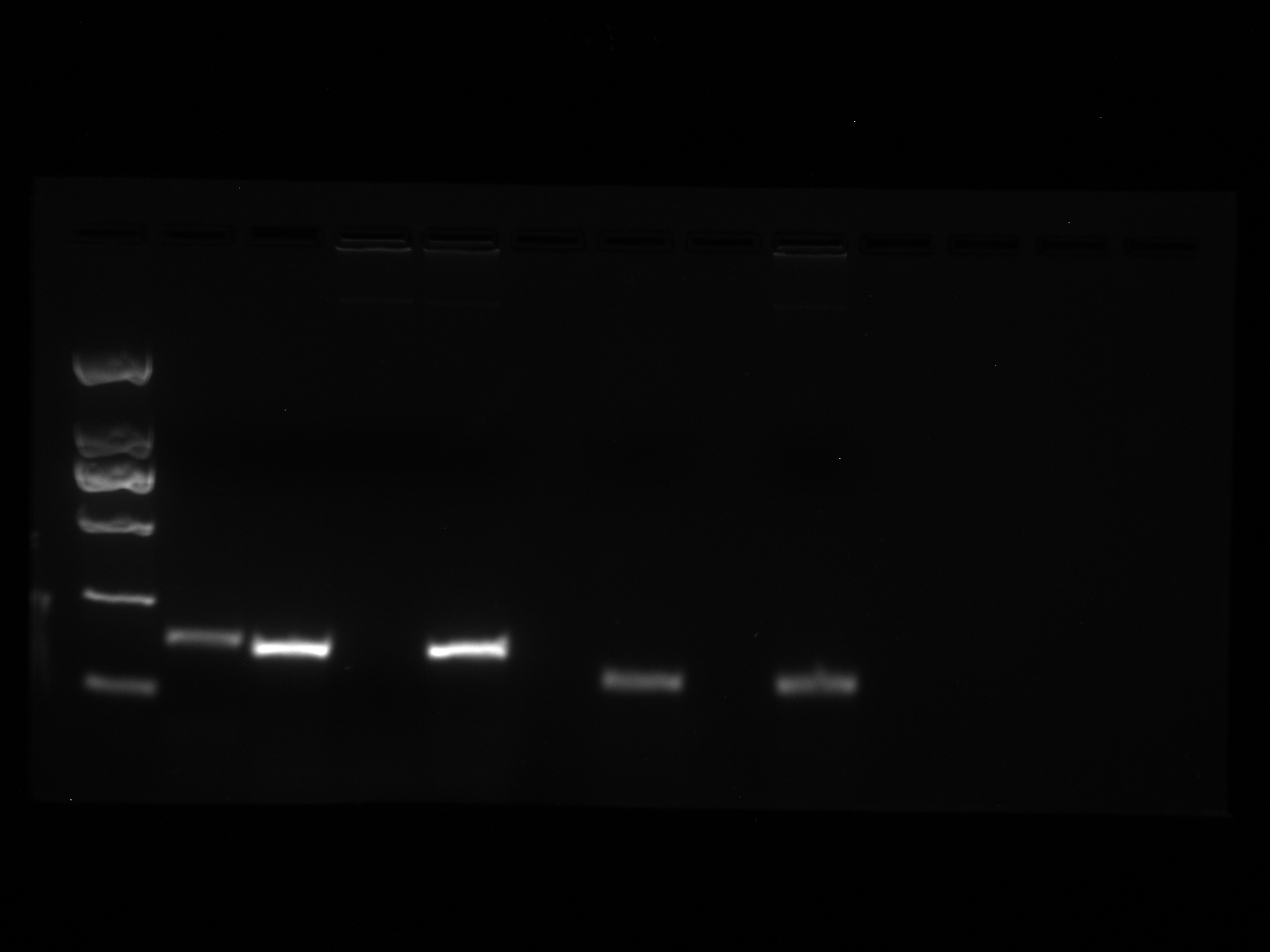

Supplement: Supplementary file 5 [file DataSheet2.ZIP › Figure 2/Figure 2F-1.tif]

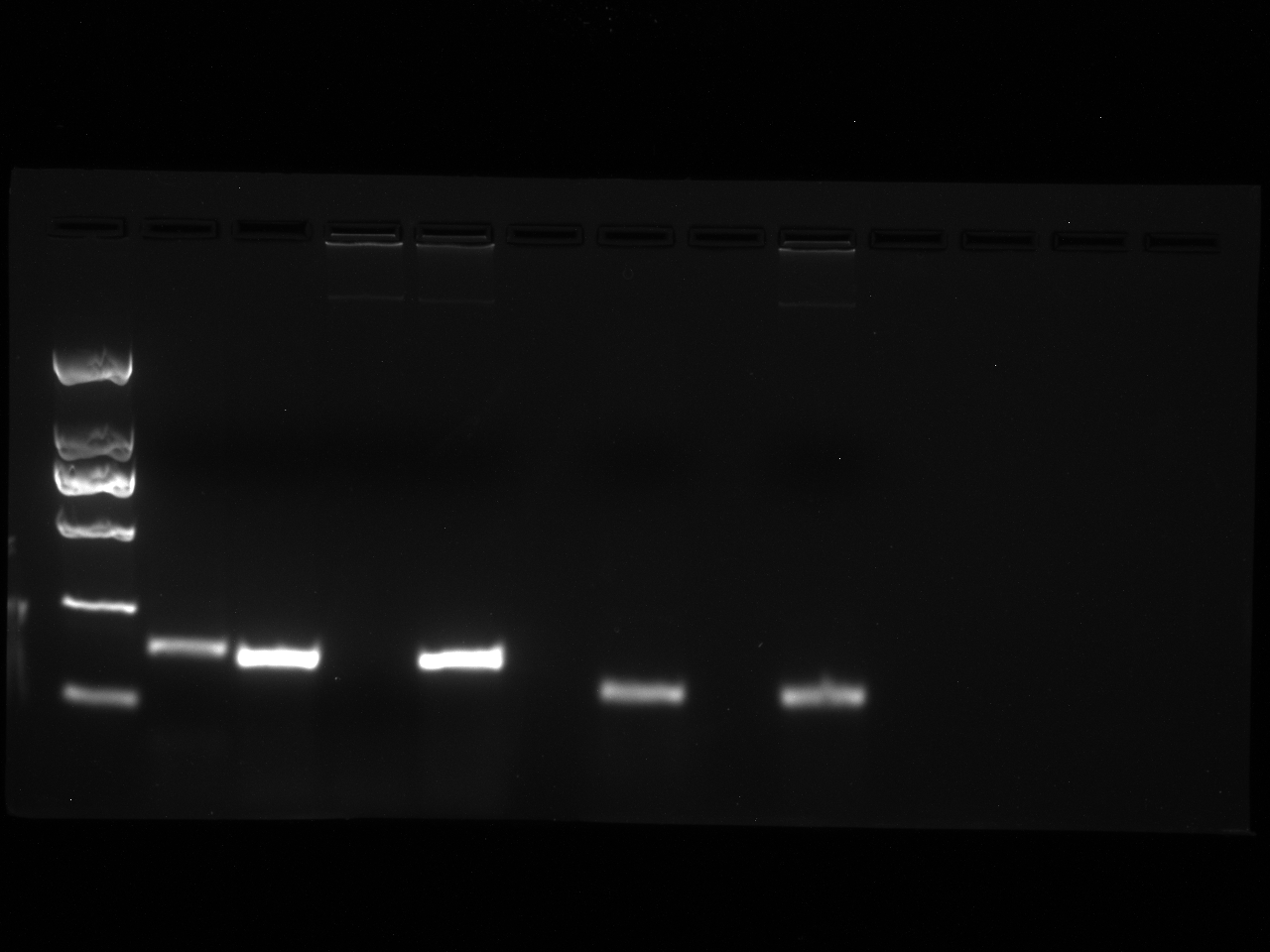

Supplement: Supplementary file 5 [file DataSheet2.ZIP › Figure 2/Figure 2F-2.tif]

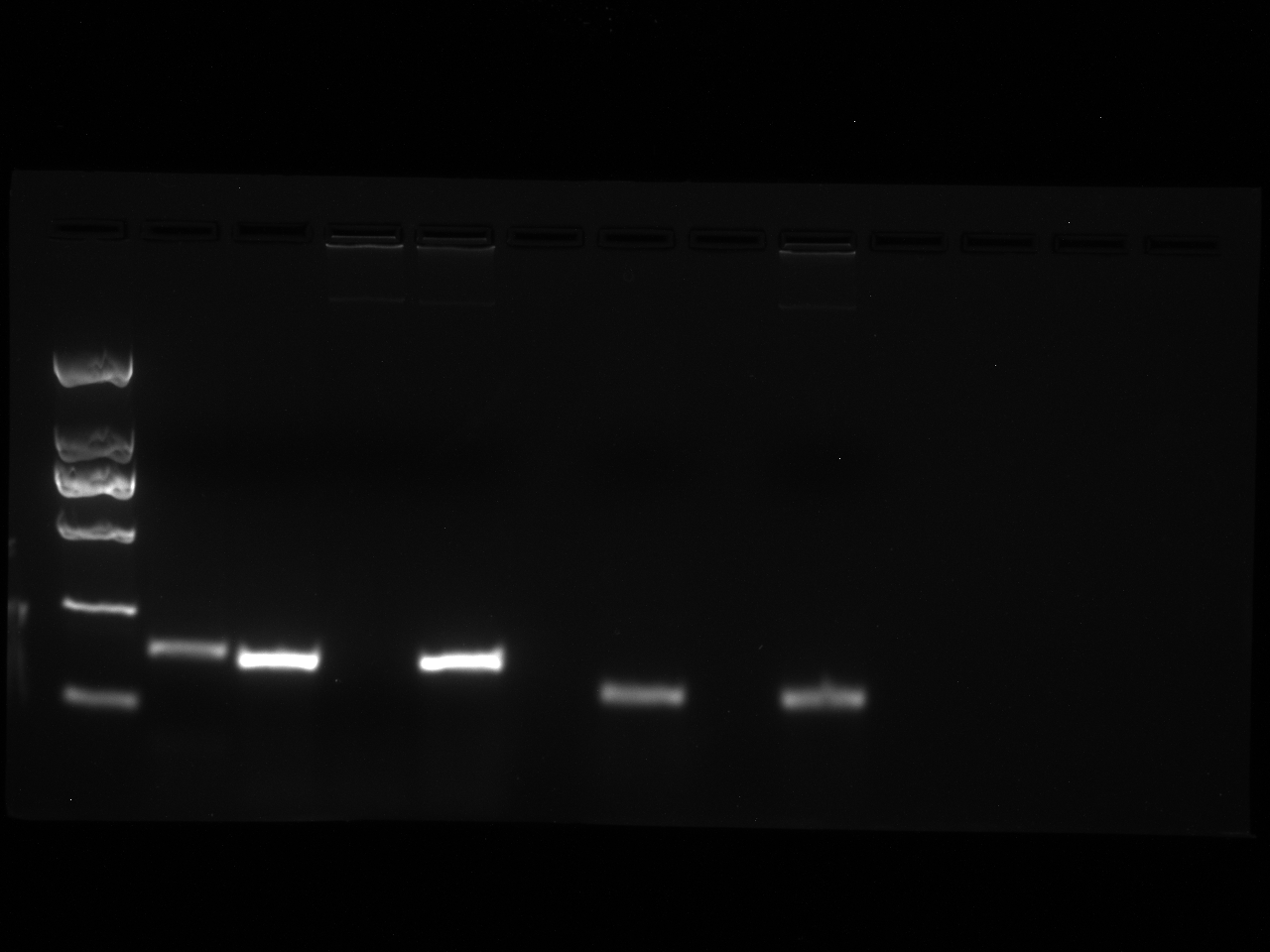

Supplement: Supplementary file 5 [file DataSheet2.ZIP › Figure 2/Figure 2F-3.tif]

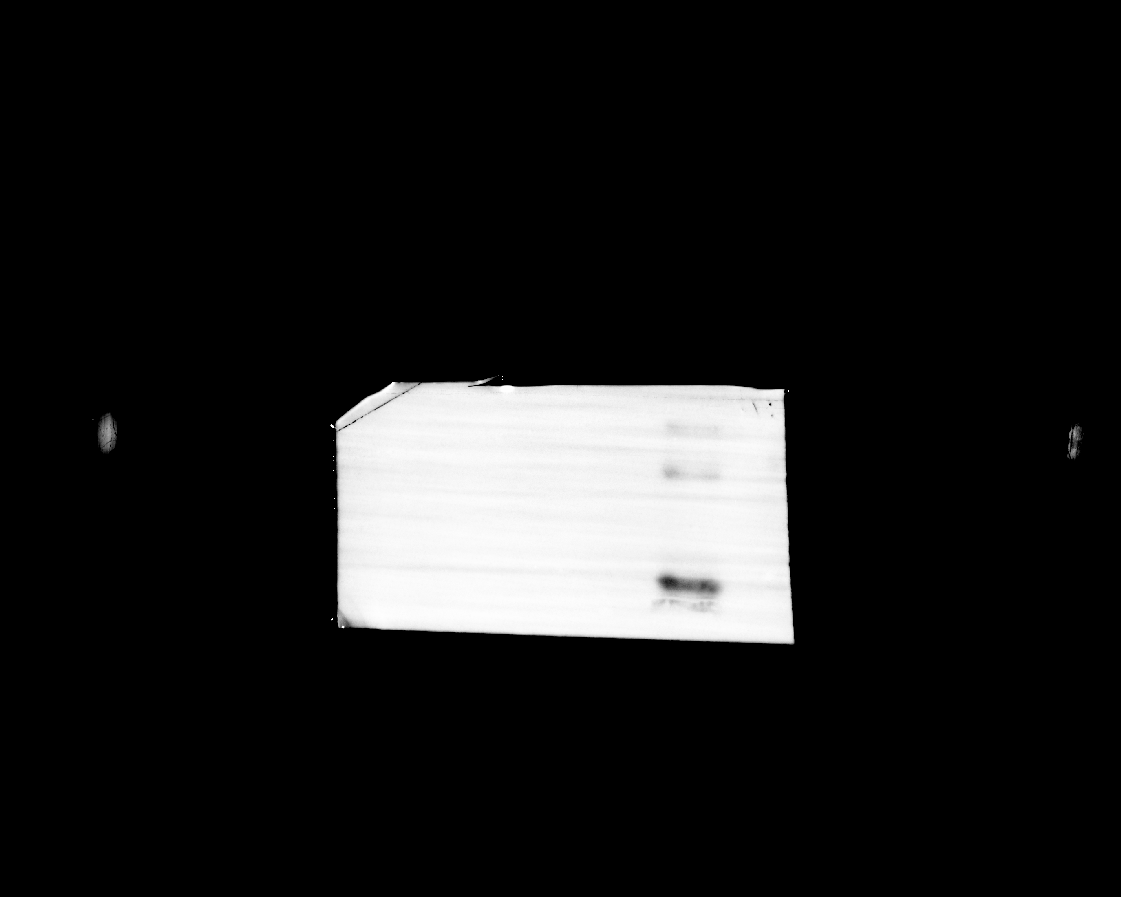

Supplement: Supplementary file 6 [file DataSheet5.ZIP › Figure 5/k562 and k562 exo cd63 marker.tif]

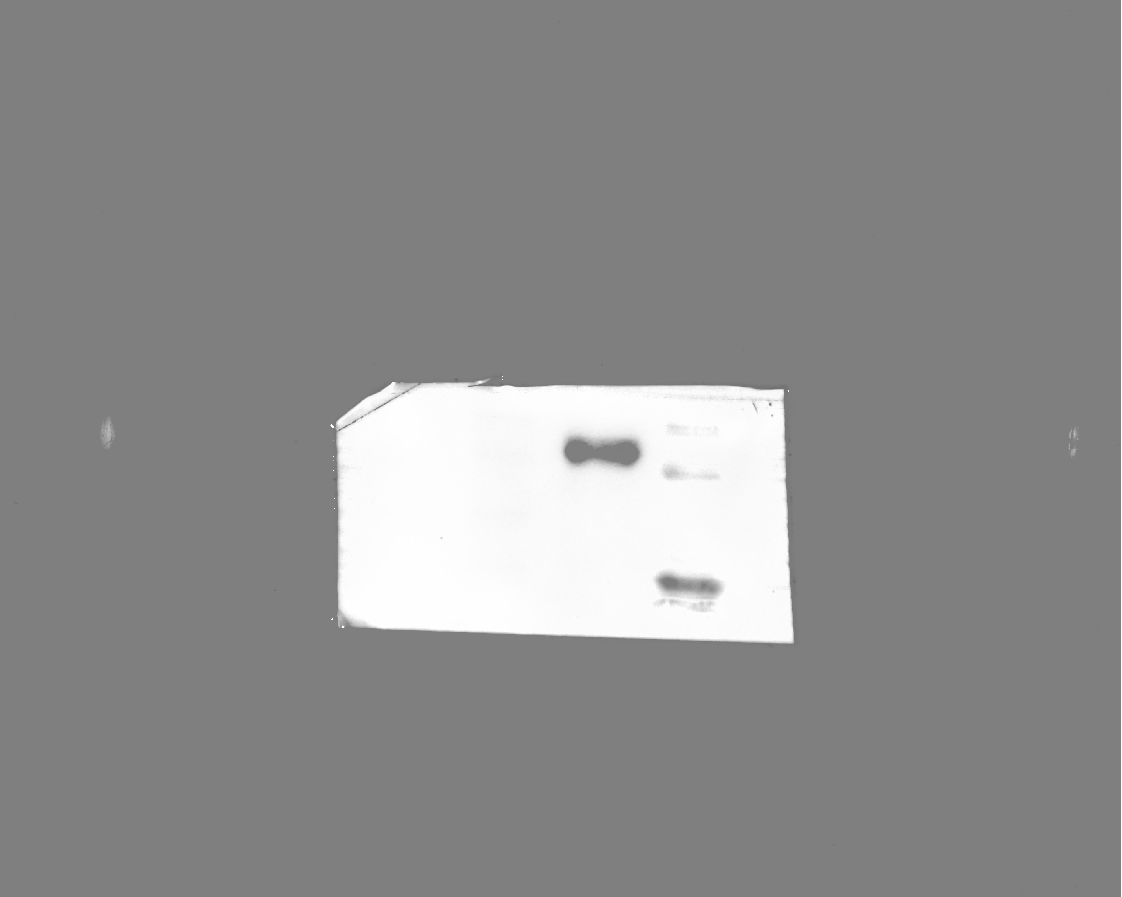

Supplement: Supplementary file 6 [file DataSheet5.ZIP › Figure 5/k562 and k562 exo cd63 merge.tif]

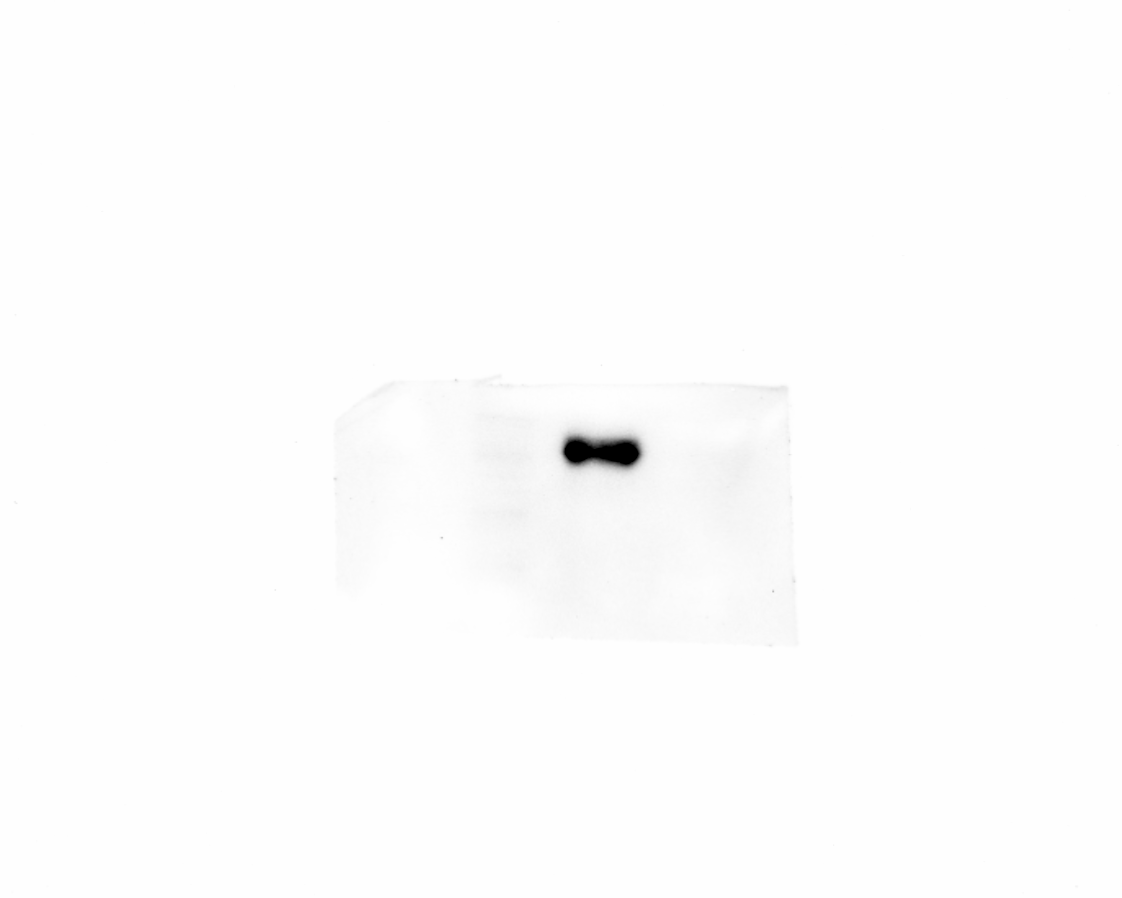

Supplement: Supplementary file 6 [file DataSheet5.ZIP › Figure 5/k562 and k562 exo cd63.tif]

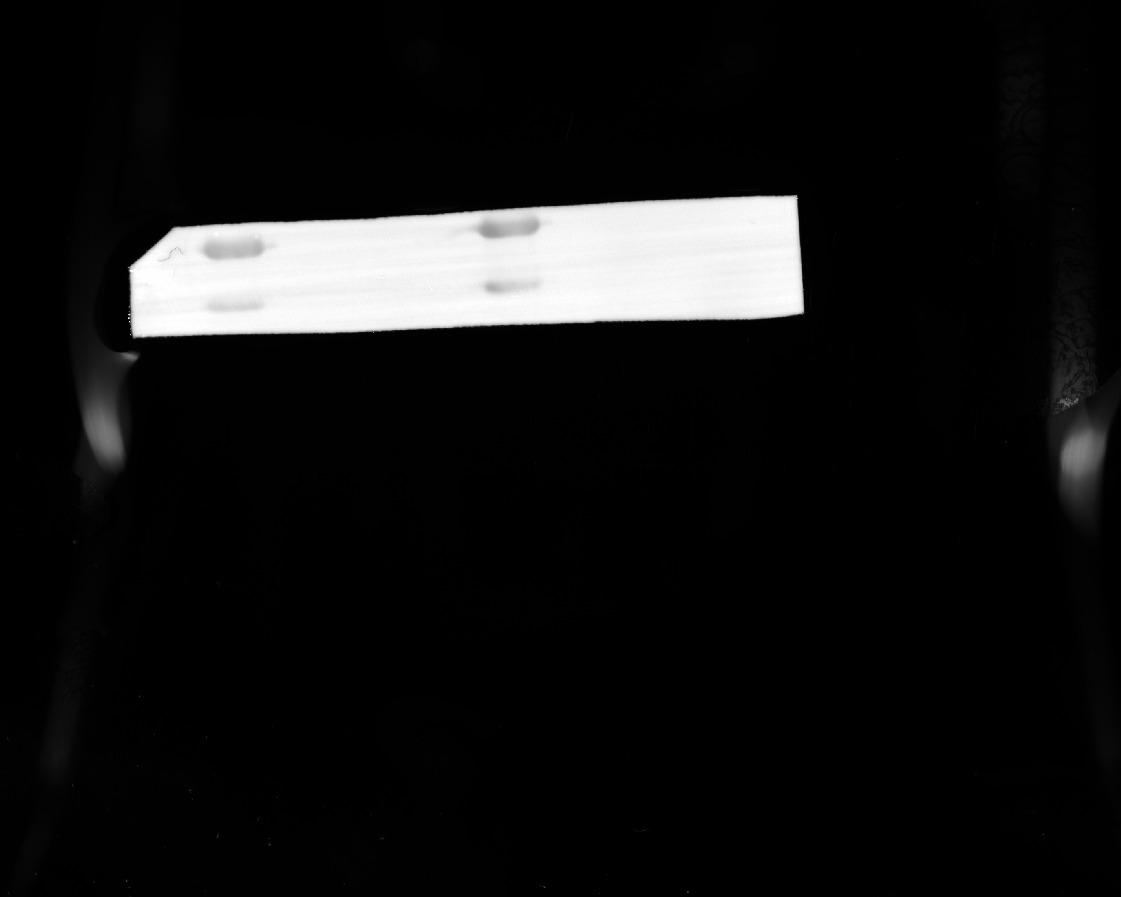

Supplement: Supplementary file 6 [file DataSheet5.ZIP › Figure 5/k562 and k562 exo tsg101 marker.tif]

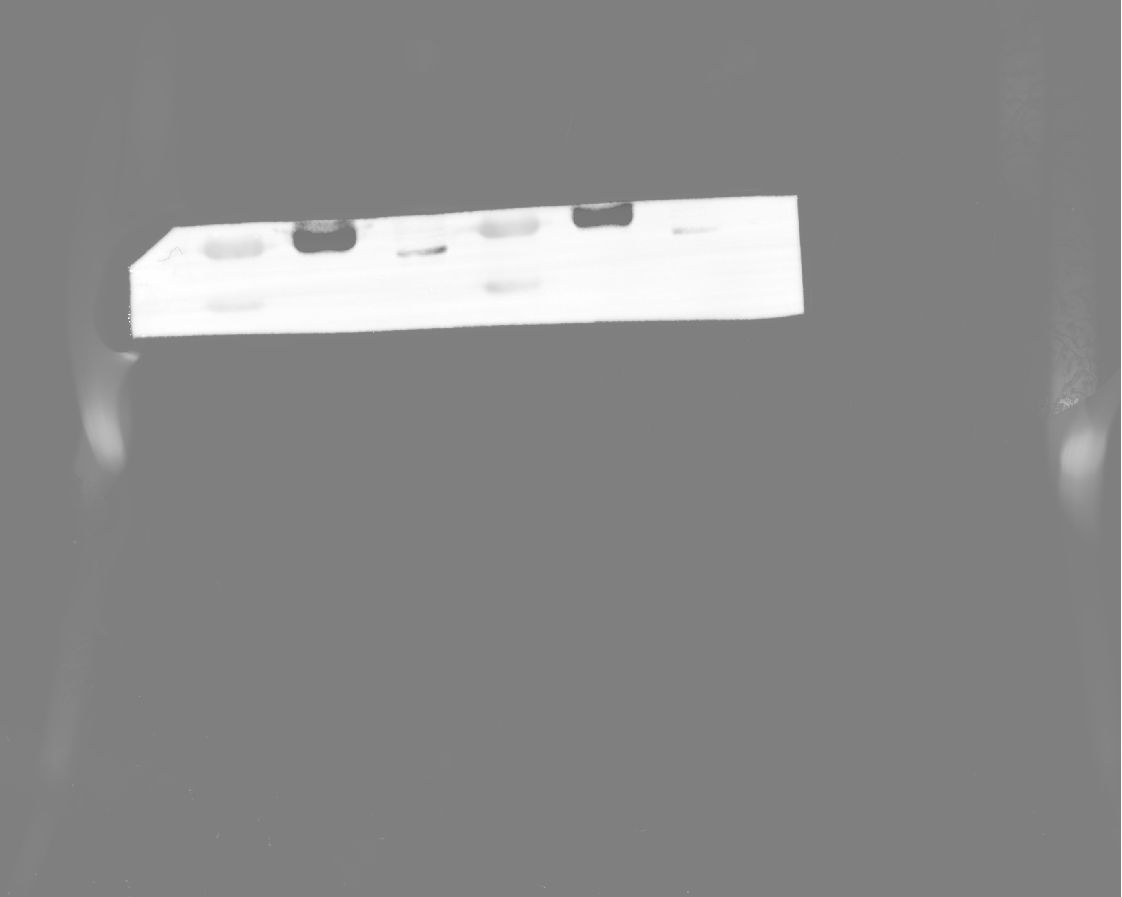

Supplement: Supplementary file 6 [file DataSheet5.ZIP › Figure 5/k562 and k562 exo tsg101 merge.tif]

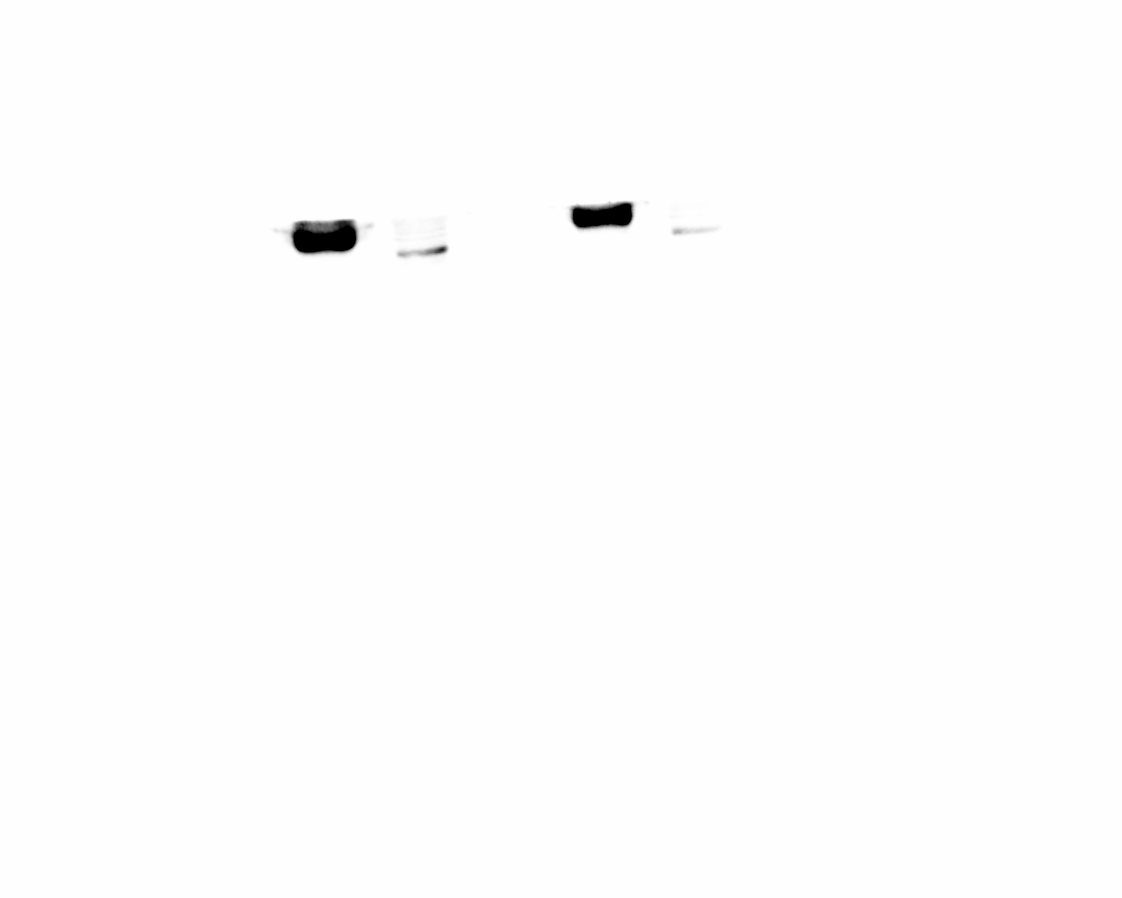

Supplement: Supplementary file 6 [file DataSheet5.ZIP › Figure 5/k562 and k562 exo tsg101.tif]

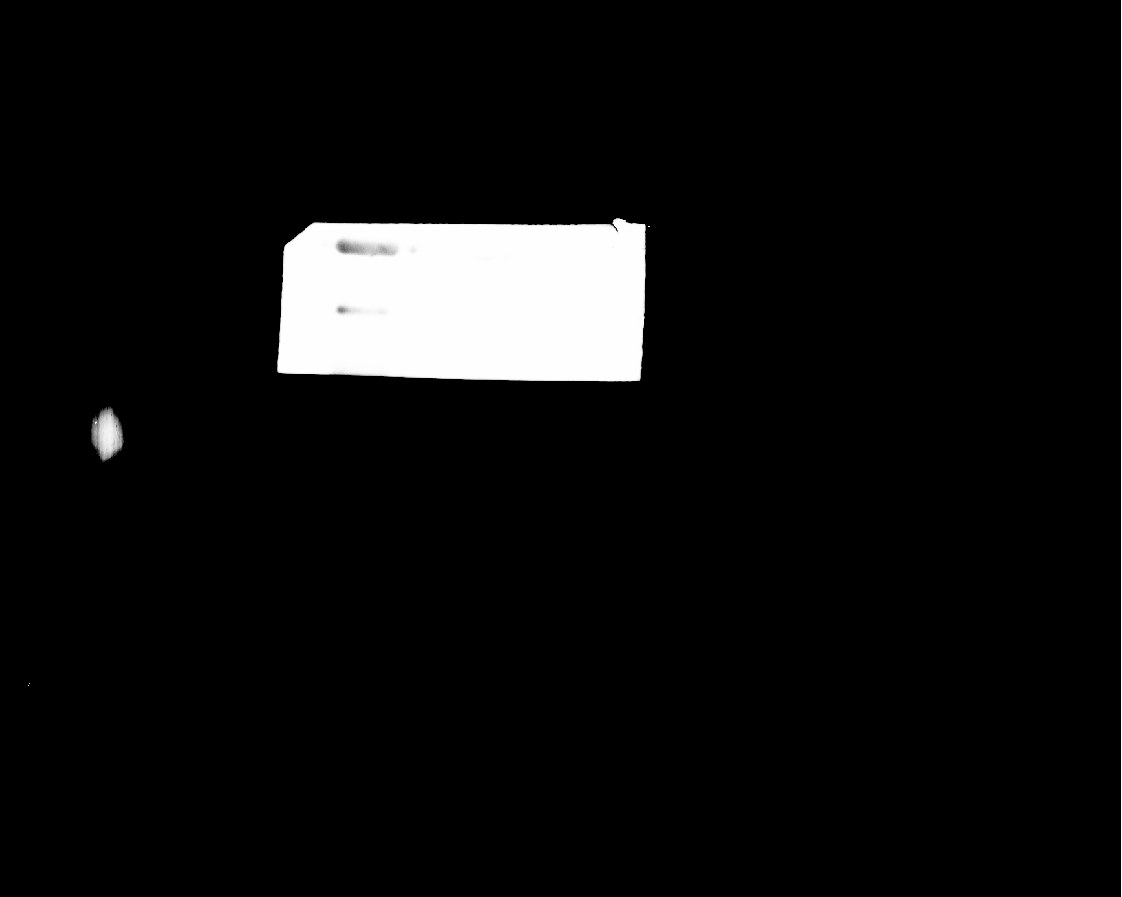

Supplement: Supplementary file 6 [file DataSheet5.ZIP › Figure 5/k562 and k562 exo tublin marker.tif]

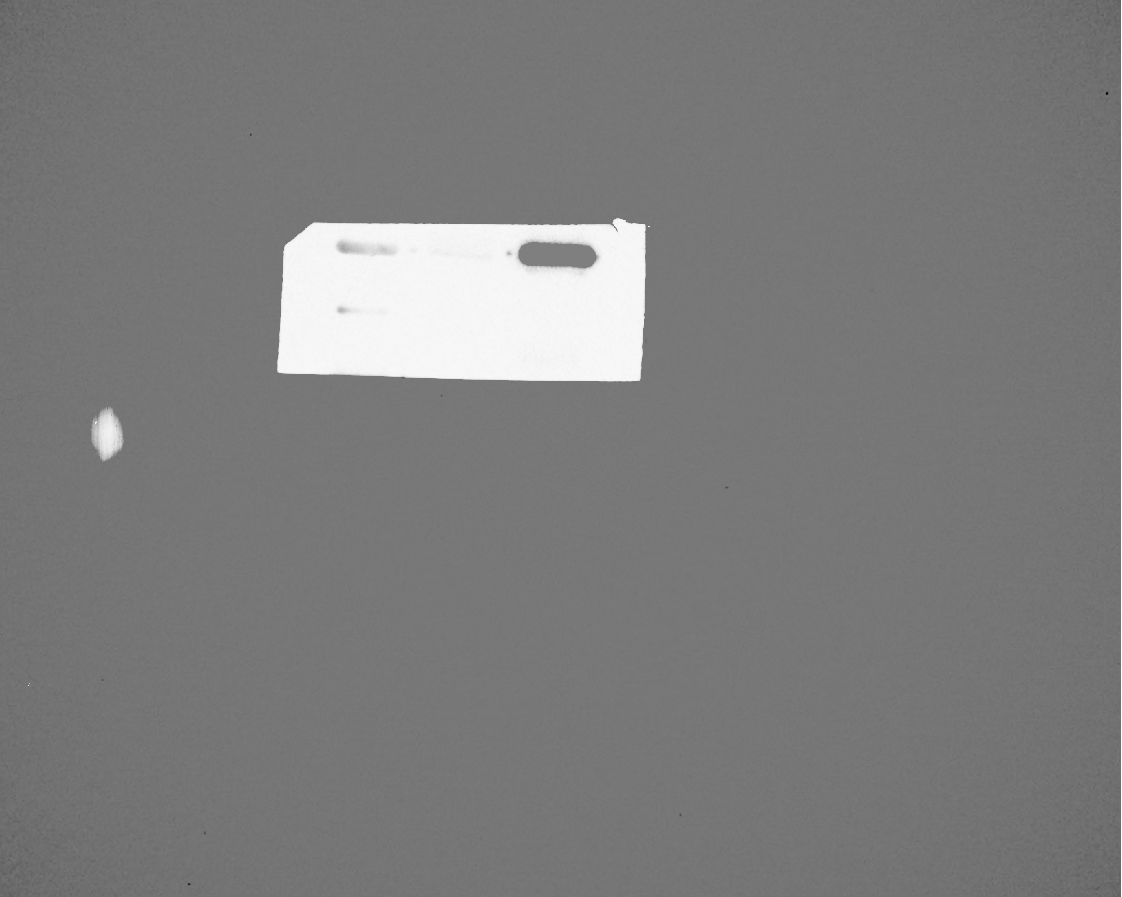

Supplement: Supplementary file 6 [file DataSheet5.ZIP › Figure 5/k562 and k562 exo tublin merge.tif]

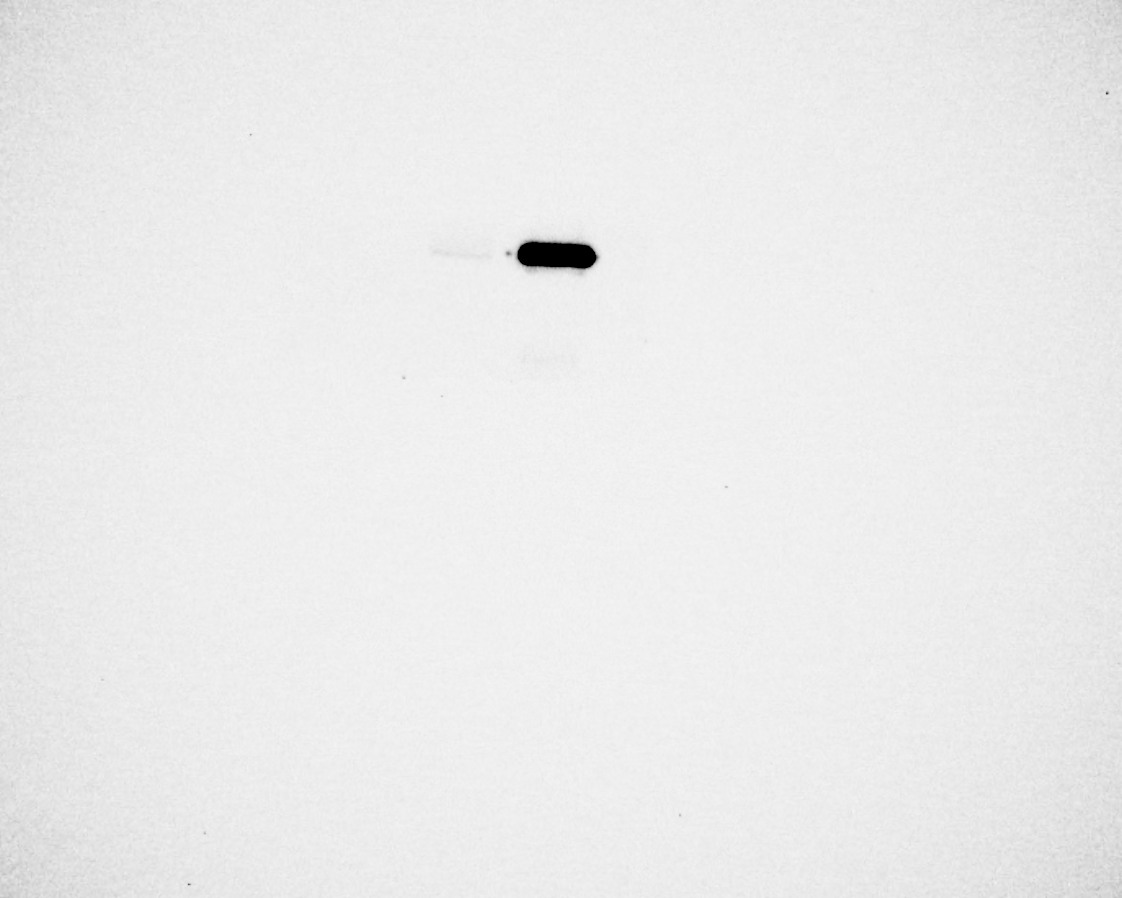

Supplement: Supplementary file 6 [file DataSheet5.ZIP › Figure 5/k562 and k562 exo tublin.tif]

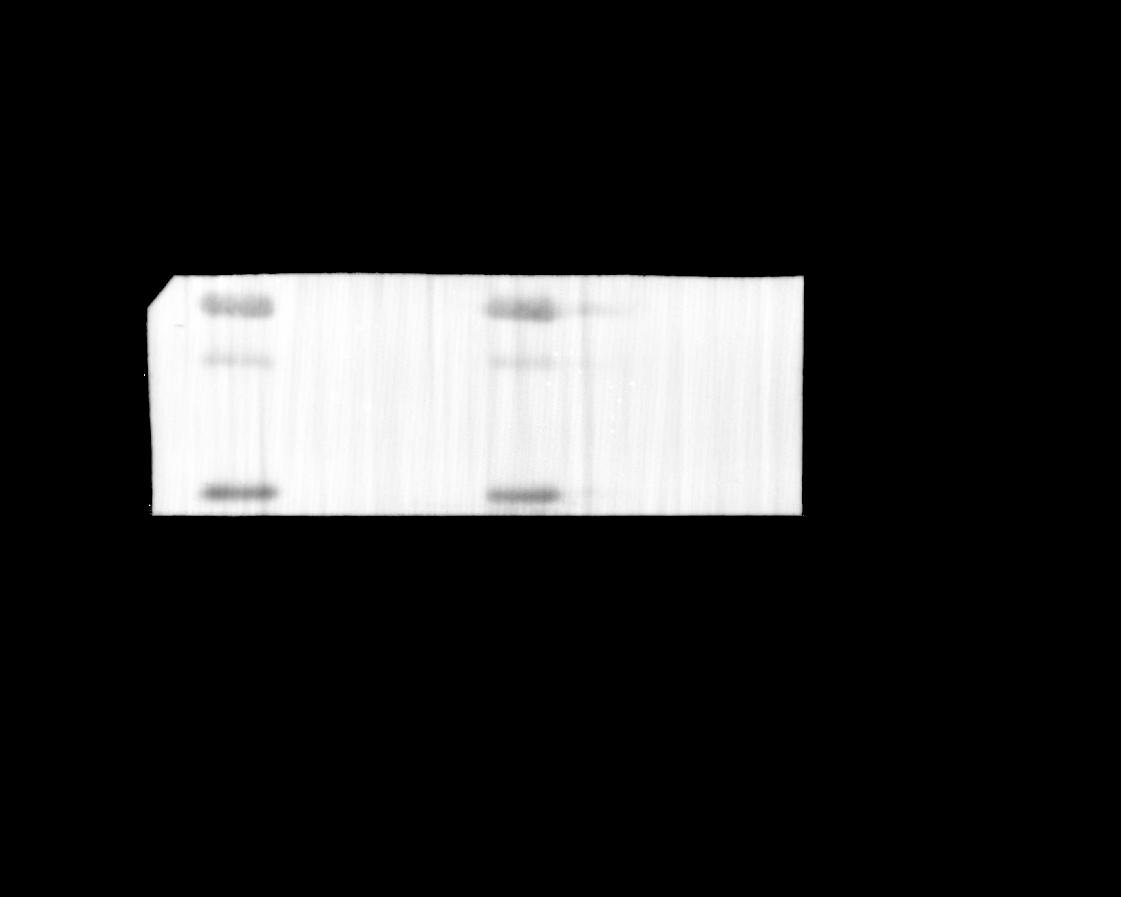

Supplement: Supplementary file 6 [file DataSheet5.ZIP › Figure 5/K562G01 and K562G01 exo CD63 marker.tif]

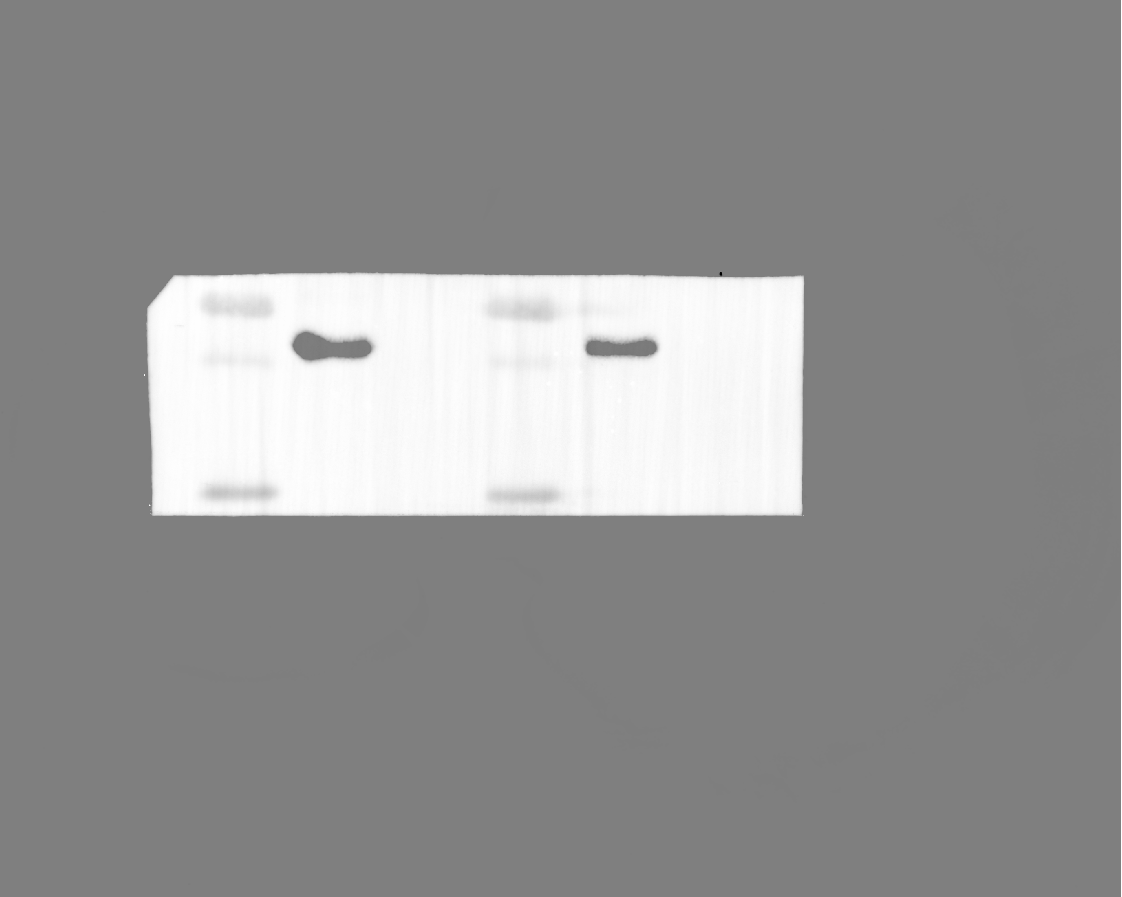

Supplement: Supplementary file 6 [file DataSheet5.ZIP › Figure 5/K562G01 and K562G01 exo CD63 merge.tif]

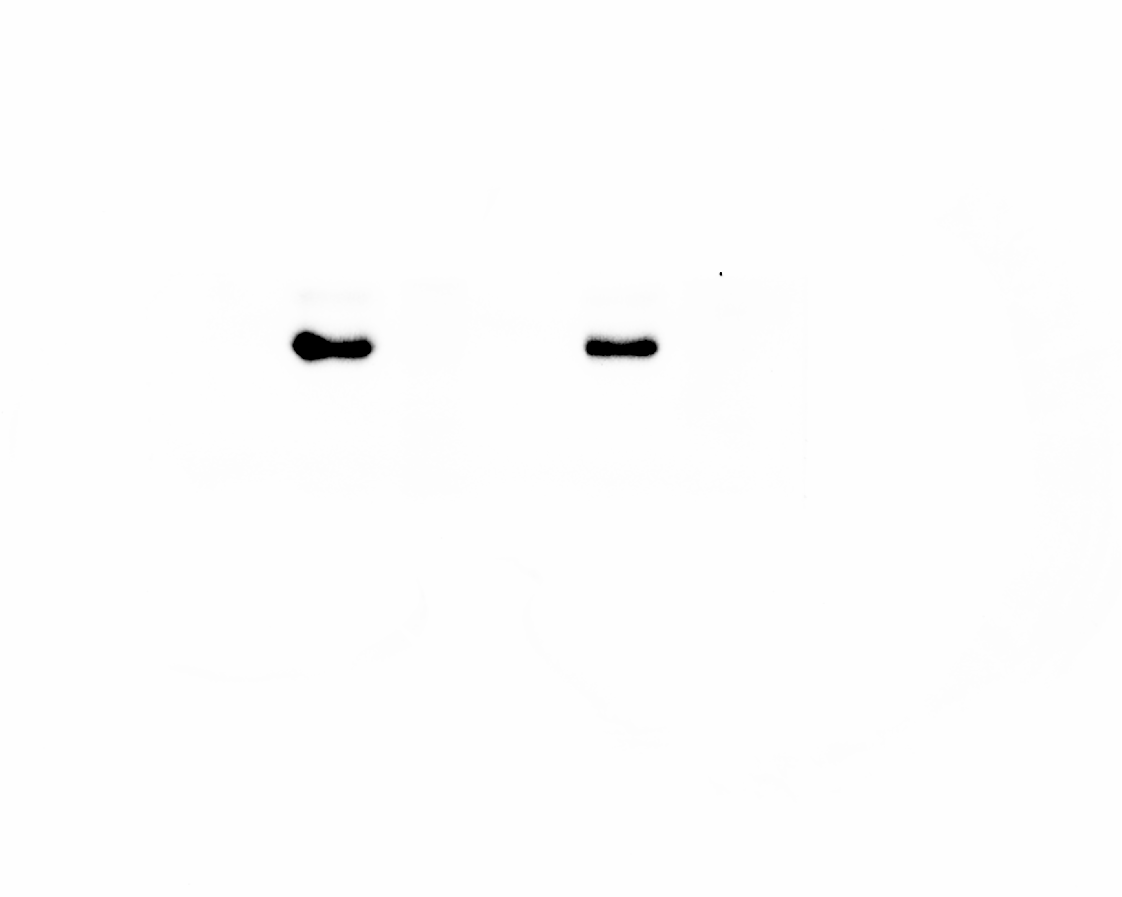

Supplement: Supplementary file 6 [file DataSheet5.ZIP › Figure 5/K562G01 and K562G01 exo CD63.tif]

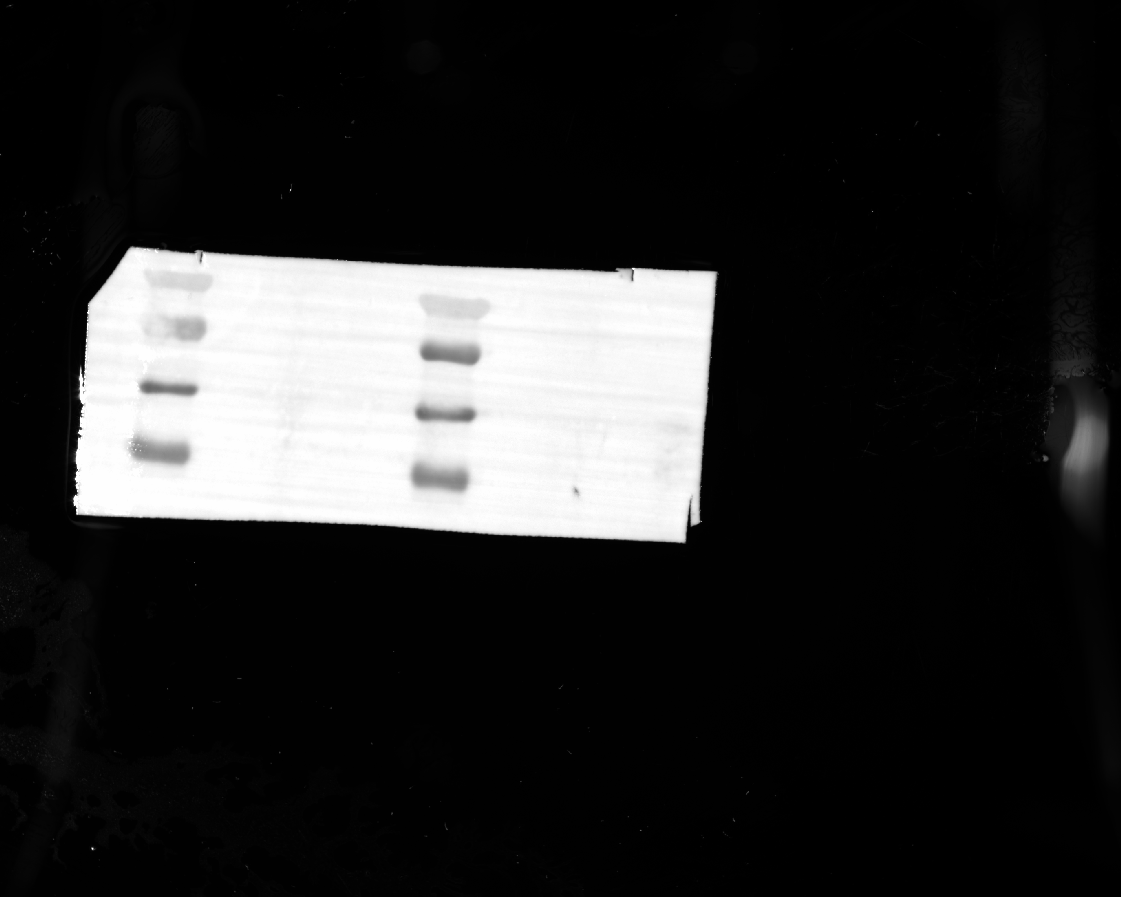

Supplement: Supplementary file 6 [file DataSheet5.ZIP › Figure 5/K562G01 and K562G01 exo tsg101 marker.tif]

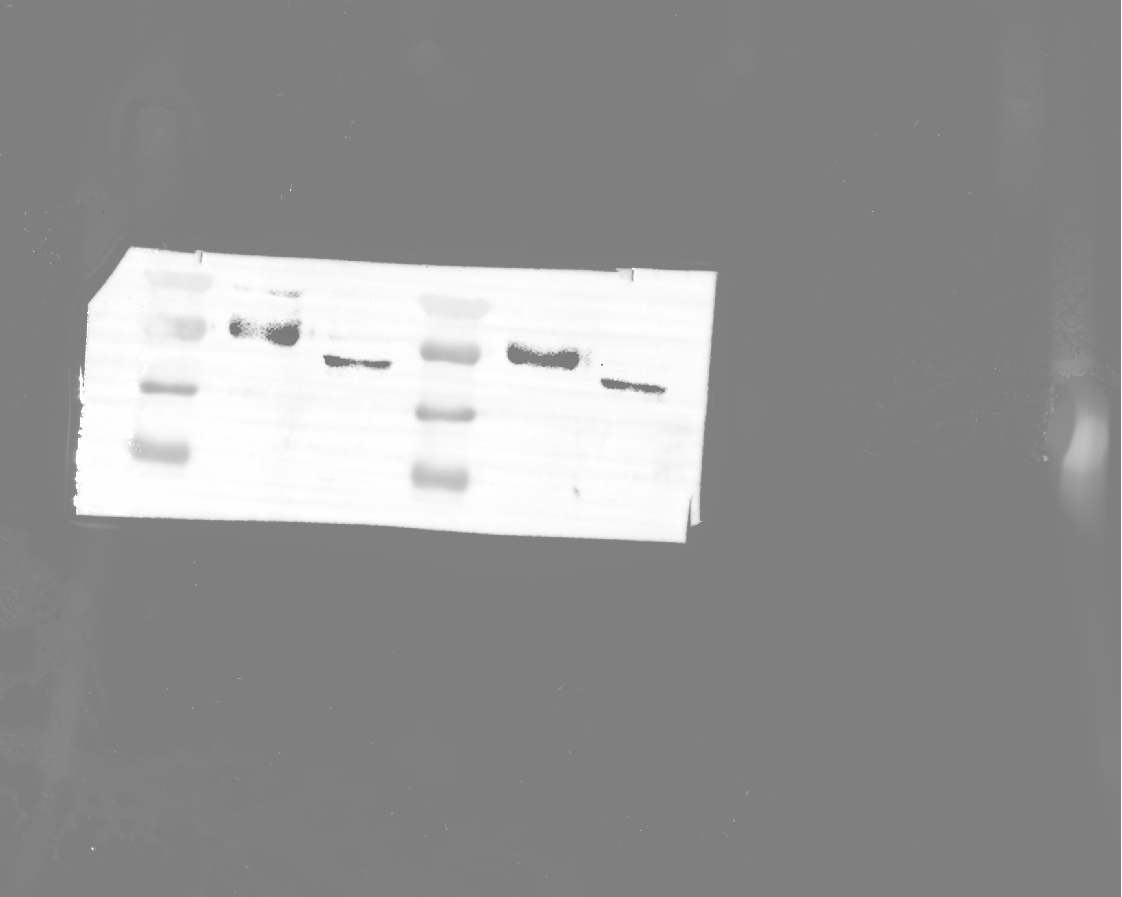

Supplement: Supplementary file 6 [file DataSheet5.ZIP › Figure 5/K562G01 and K562G01 exo tsg101 merge.tif]

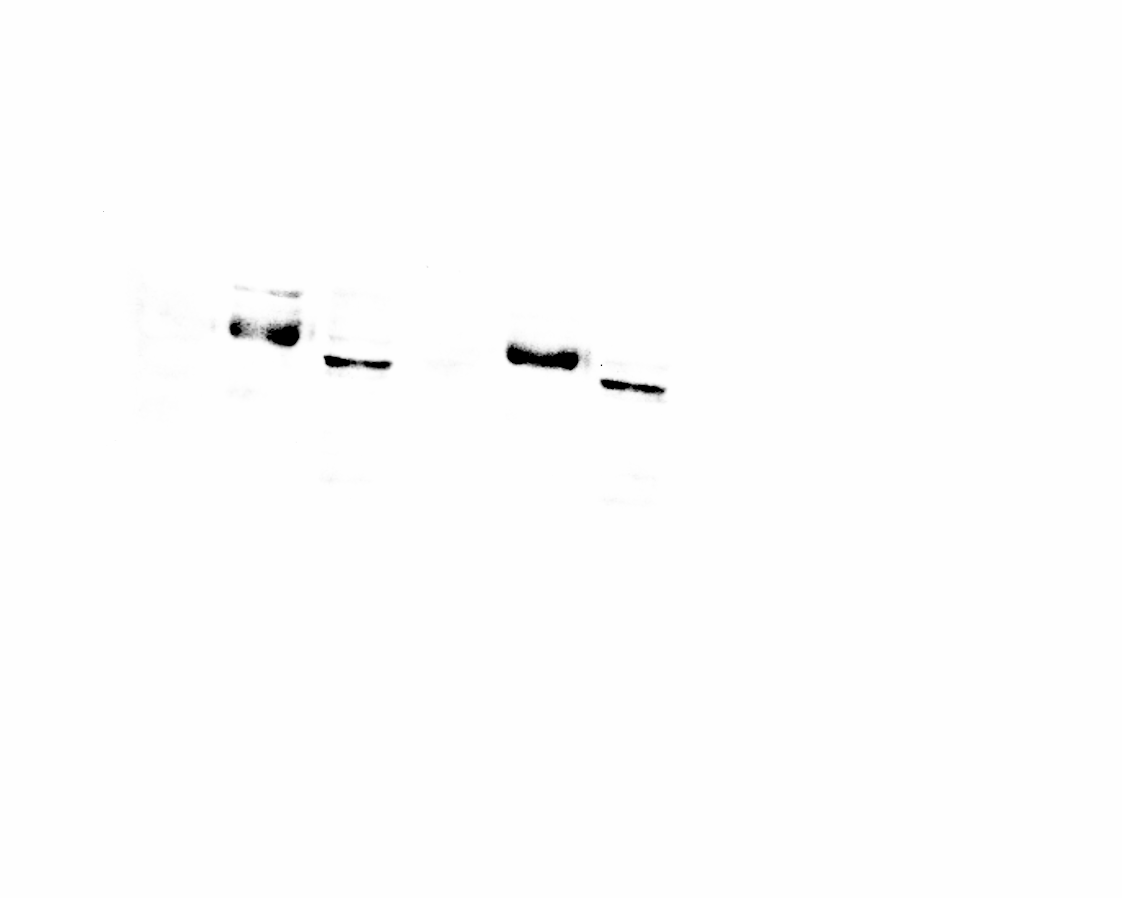

Supplement: Supplementary file 6 [file DataSheet5.ZIP › Figure 5/K562G01 and K562G01 exo tsg101.tif]

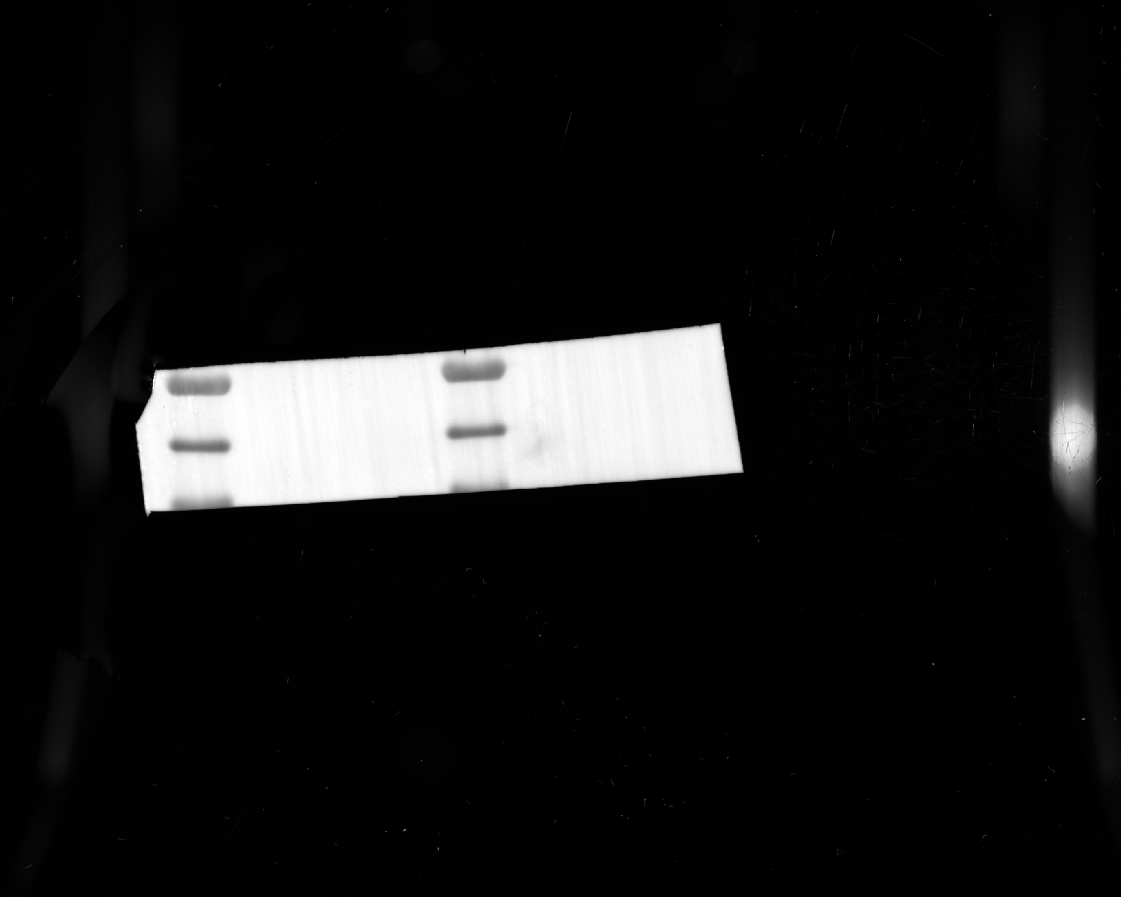

Supplement: Supplementary file 6 [file DataSheet5.ZIP › Figure 5/K562G01 and K562G01 exo tublin marker.tif]

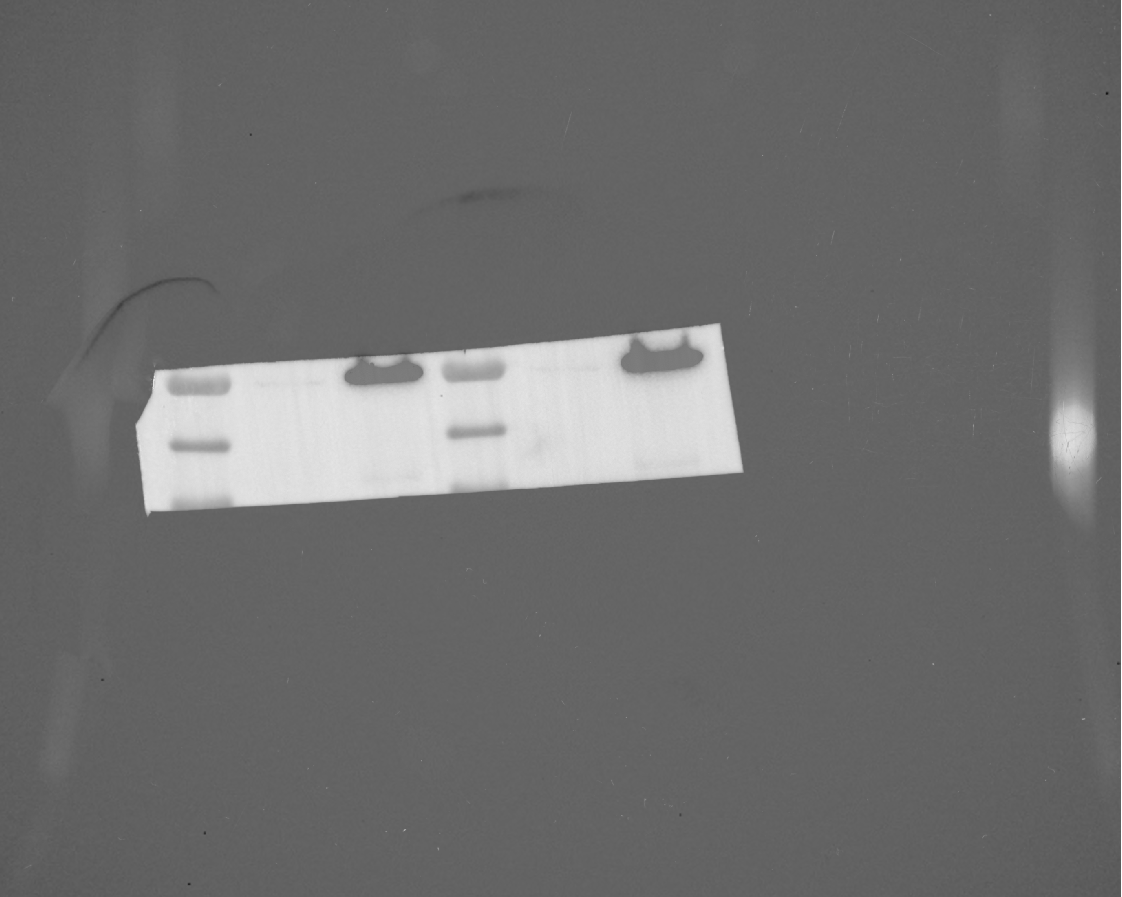

Supplement: Supplementary file 6 [file DataSheet5.ZIP › Figure 5/K562G01 and K562G01 exo tublin merge.tif]

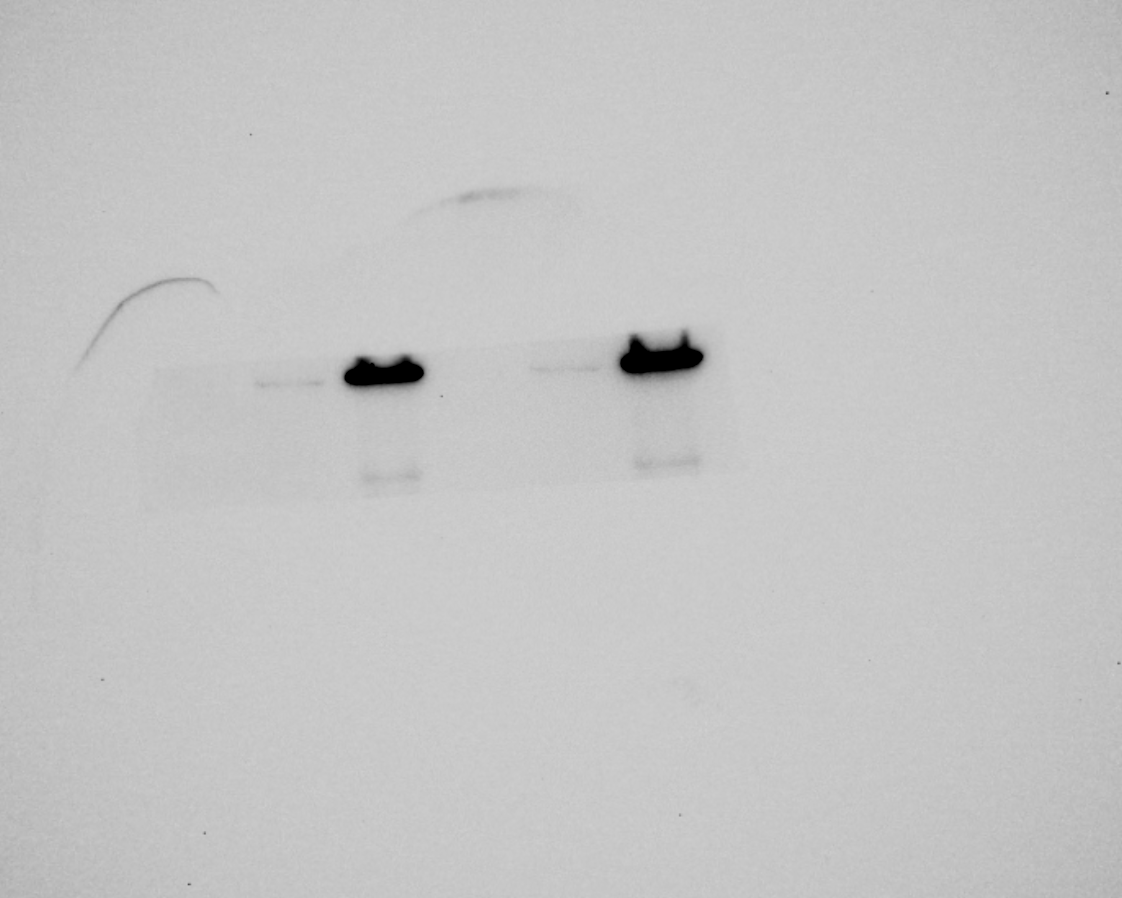

Supplement: Supplementary file 6 [file DataSheet5.ZIP › Figure 5/K562G01 and K562G01 exo tublin.tif]
