# Supplementary material for: CircRNA Microarray Profiling Reveals hsa_circ_0058493 as a Novel Biomarker for Imatinib-Resistant CML
Source: Front Pharmacol. 2021 Sep 13;12:728916. doi: 10.3389/fphar.2021.728916 (PMC8473700; doi:10.3389/fphar.2021.728916)
Supplement: Supplementary file 6 [file DataSheet5.ZIP › Figure 5/Figure 5C K562-exo NTA.pdf]

NANOSIGHT

K-exo 20210125 2021-01-25 11-13-02

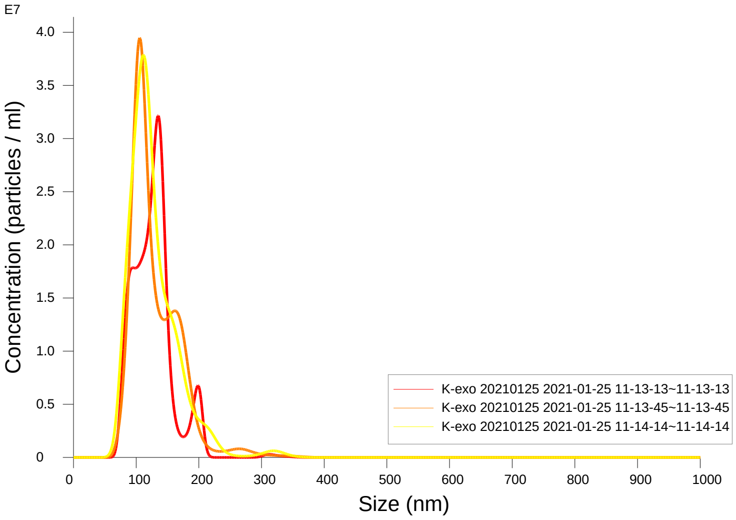

FTLA Concentration / Size graph for Experiment:
K-exo 20210125 2021-01-25 11-13-02

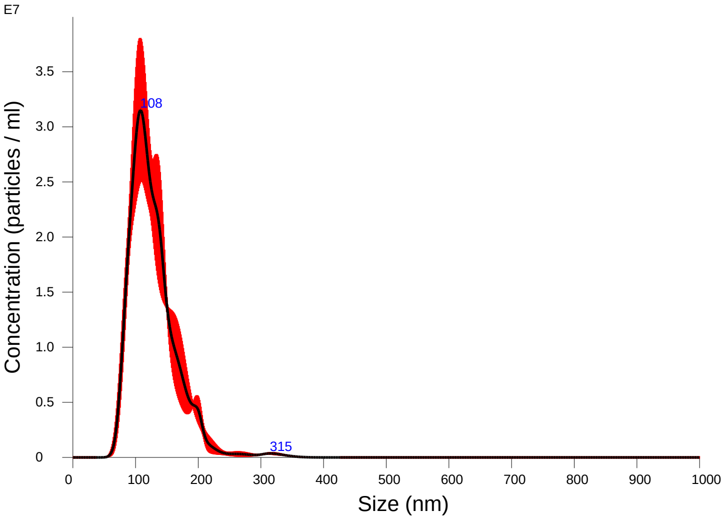

Averaged FTLA Concentration / Size for Experiment:
K-exo 20210125 2021-01-25 11-13-02
Error bars indicate + / -1 standard error of the mean

Included Files

K-exo 20210125 2021-01-25 11-13-13
K-exo 20210125 2021-01-25 11-13-45
K-exo 20210125 2021-01-25 11-14-14

Details

NTA Version:	NTA 3.4 - Sample Assistant Build 3.4.003-SA
Script Used:	SOP Standard Measurement 11-13-02AM 25J~
Time Captured:	11:13:02 25/01/2021
Operator:	ryan
Pre-treatment:	
Sample Name:	
Diluent:	
Remarks:	

Capture Settings

Camera Type:	sCMOS
Laser Type:	Blue488
Camera Level:	12
Slider Shutter:	1200
Slider Gain:	125
FPS	25.0
Number of Frames:	499
Temperature:	21.0 ^{o}C
Viscosity:	(Water) 1.0 cP
Dilution factor:	Dilution not recorded

Analysis Settings

Detect Threshold:	3
Blur Size:	Auto
Max Jump Distance:	Auto: 12.6 - 12.9 pix

Results

Stats: Merged Data

Mean:	128.1 nm
Mode:	107.9 nm
SD:	38.2 nm
D10:	89.0 nm
D50:	120.5 nm
D90:	176.0 nm

Stats: Mean +/- Standard Error

Mean:	128.1 +/- 0.9 nm
Mode:	117.4 +/- 9.0 nm
SD:	37.7 +/- 2.3 nm
D10:	89.0 +/- 1.3 nm
D50:	120.8 +/- 2.7 nm
D90:	172.9 +/- 4.4 nm
Concentration (Upgrade):	2.20e+09 +/- 1.94e+08 particles/ml
	245.1 +/- 24.4 particles/frame
	197.8 +/- 14.2 centres/frame

NANOSIGHT

K-exo 20210125 2021-01-25 11-13-02

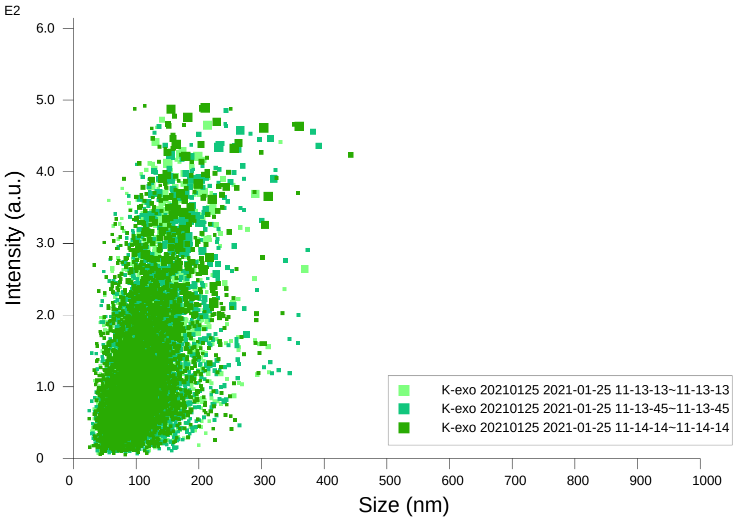

Intensity / Size graph for Experiment:
K-exo 20210125 2021-01-25 11-13-02

Script Used: (Full Text):

SOP Standard Measurement 11-13-02AM 25Jan2021.txt
