# Supplementary material for: CircRNA Microarray Profiling Reveals hsa_circ_0058493 as a Novel Biomarker for Imatinib-Resistant CML
Source: Front Pharmacol. 2021 Sep 13;12:728916. doi: 10.3389/fphar.2021.728916 (PMC8473700; doi:10.3389/fphar.2021.728916)
Supplement: Supplementary file 6 [file DataSheet5.ZIP › Figure 5/Figure 5C K562G01-exo NTA.pdf]

NANOSIGHT

KG-exo 2021-01-25 2021-01-25 11-22-45

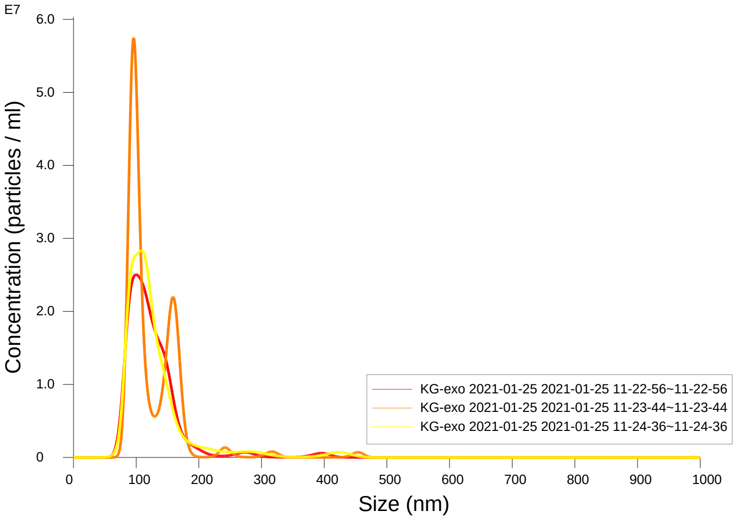

FTLA Concentration / Size graph for Experiment:
KG-exo 2021-01-25 2021-01-25 11-22-45

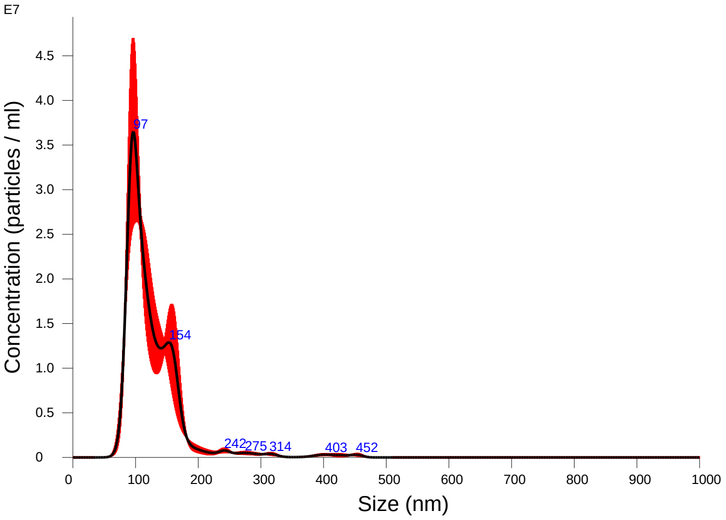

Averaged FTLA Concentration / Size for Experiment:
KG-exo 2021-01-25 2021-01-25 11-22-45
Error bars indicate + / -1 standard error of the mean

Included Files

KG-exo 2021-01-25 2021-01-25 11-22-56
KG-exo 2021-01-25 2021-01-25 11-23-44
KG-exo 2021-01-25 2021-01-25 11-24-36

Details

NTA Version:	NTA 3.4 - Sample Assistant Build 3.4.003-SA
Script Used:	SOP Standard Measurement 11-22-45AM 25J~
Time Captured:	11:22:45 25/01/2021
Operator:	ryan
Pre-treatment:	
Sample Name:	
Diluent:	
Remarks:	

Capture Settings

Camera Type:	sCMOS
Laser Type:	Blue488
Camera Level:	12
Slider Shutter:	1200
Slider Gain:	125
FPS	25.0
Number of Frames:	499
Temperature:	21.3 - 21.3 ^{o}C
Viscosity:	(Water) 0.968 - 0.970 cP
Dilution factor:	Dilution not recorded

Analysis Settings

Detect Threshold:	3
Blur Size:	Auto
Max Jump Distance:	Auto: 12.9 - 13.2 pix

Results

Stats: Merged Data

Mean:	126.3 nm
Mode:	96.4 nm
SD:	51.1 nm
D10:	87.6 nm
D50:	112.1 nm
D90:	166.8 nm

Stats: Mean +/- Standard Error

Mean:	126.3 +/- 1.7 nm
Mode:	101.4 +/- 3.7 nm
SD:	50.7 +/- 3.7 nm
D10:	87.4 +/- 0.6 nm
D50:	112.1 +/- 3.3 nm
D90:	168.2 +/- 4.0 nm
Concentration (Upgrade):	1.90e+09 +/- 1.00e+08 particles/ml
	206.2 +/- 8.7 particles/frame
	176.3 +/- 5.0 centres/frame

NANOSIGHT

KG-exo 2021-01-25 2021-01-25 11-22-45

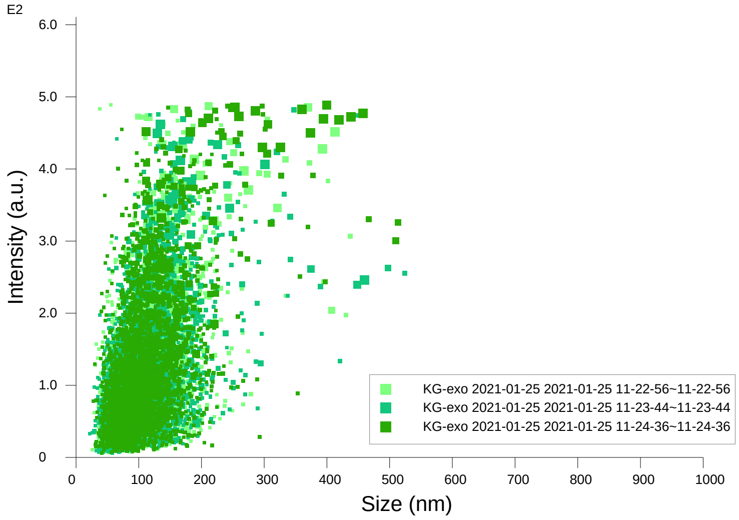

Intensity / Size graph for Experiment:
KG-exo 2021-01-25 2021-01-25 11-22-45

Script Used: (Full Text):

SOP Standard Measurement 11-22-45AM 25Jan2021.txt
